# Detecting Local Adaptation between North and South European Atlantic Salmon Populations

**DOI:** 10.3390/biology11060933

**Published:** 2022-06-19

**Authors:** María Gabián, Paloma Morán, María Saura, Antonio Carvajal-Rodríguez

**Affiliations:** 1 Centro de Investigación Mariña (CIM), Departamento de Bioquímica, Genética e Inmunología, Universidade de Vigo, 36310 Vigo, Spain; gabianmaria@gmail.com (M.G.); paloma@uvigo.es (P.M.); 2 Departamento de Mejora Genética Animal, Instituto Nacional de Investigación y Tecnología Agraria y Alimentaria (INIA-CSIC), 28040 Madrid, Spain; saura.maria@inia.csic.es

**Keywords:** Atlantic salmon, divergent selection, local adaptation, NADP-dependent malic enzyme-2, SNP array

## Abstract

**Simple Summary:**

Atlantic salmon is a diadromous species that has the particularity that it returns to reproduce to its river of birth. This means that, in most cases, each river can be considered a population and that these populations can be adapted to the characteristics of the river. The Atlantic Salmon in the rivers of the Iberian Peninsula has been in decline since the 80’s of the last century. At that time, to alleviate the decline in the number of individuals, the rivers were stocked with salmon from Scotland and other northern European rivers, but the result was not positive. In this work, we studied eight salmon populations using a 220,000 high-density SNP array (Affymetrix) specific to Atlantic salmon. Our results revealed potential evidence of local adaptation for North Spanish and Scottish populations, which may be the cause of the failure of restocking programs. In addition, by performing a review of previous studies, we demonstrated that, as suggested, NADP-dependent malic enzyme-2 is a candidate for local adaptation in salmon from the Iberian Peninsula.

**Abstract:**

Pollution and other anthropogenic effects have driven a decrease in Atlantic salmon (*Salmo salar*) in the Iberian Peninsula. The restocking effort carried out in the 1980s, with salmon from northern latitudes with the aim of mitigating the decline of native populations, failed, probably due to the deficiency in adaptation of foreign salmon from northern Europe to the warm waters of the Iberian Peninsula. This result would imply that the Iberian populations of Atlantic salmon have experienced local adaptation in their past evolutionary history, as has been described for other populations of this species and other salmonids. Local adaptation can occur by divergent selections between environments, favoring the fixation of alleles that increase the fitness of a population in the environment it inhabits relative to other alleles favored in another population. In this work, we compared the genomes of different populations from the Iberian Peninsula (Atlantic and Cantabric basins) and Scotland in order to provide tentative evidence of candidate SNPs responsible for the adaptive differences between populations, which may explain the failures of restocking carried out during the 1980s. For this purpose, the samples were genotyped with a 220,000 high-density SNP array (Affymetrix) specific to Atlantic salmon. Our results revealed potential evidence of local adaptation for North Spanish and Scottish populations. As expected, most differences concerned the comparison of the Iberian Peninsula with Scotland, although there were also differences between Atlantic and Cantabric populations. A high proportion of the genes identified are related to development and cellular metabolism, DNA transcription and anatomical structure. A particular SNP was identified within the NADP-dependent malic enzyme-2 (_m_MEP-2*), previously reported by independent studies as a candidate for local adaptation in salmon from the Iberian Peninsula. Interestingly, the corresponding SNP within the _m_MEP-2* region was consistent with a genomic pattern of divergent selection.

## 1. Introduction

Wild populations of Atlantic salmon (*Salmo salar*) from the Iberian Peninsula suffered a drastic decrease in the last three decades of the 20th century, mainly due to anthropogenic actions such as pollution [1], the construction of physical barriers that reduced the habitat, and the increase in overfishing [2,3].

To reduce this drastic decline, efforts were focused on restocking from 1981 to 1994, not only with eggs from the wild but also purchased from fish farms, mainly from rivers located in Scotland and Norway. Restocking was done by planting eggs into the river or by releasing fry previously hatched in local fish hatcheries (a practice known as supportive breeding (see [4] and references therein). Nevertheless, there was no increase in the effective population size, suggesting that the introduction of foreign genomes may have contributed even more to the decline of some populations [5,6].

The restocking fail was probably due to a deficiency in the adaptation of salmon from northern Europe to the warm waters of the Iberian Peninsula since this area constitutes the southern limit of distribution of this species in Europe [7]. In fact, local adaptation has been described in previous studies for other populations of this species and salmonids in general [8,9,10,11]. Therefore, it seems that the Iberian populations of Atlantic salmon may have also experienced local adaptation at some point in their evolutionary history.

To investigate the causes of the deficient adaptation of the northern European populations to the rivers of the Iberian Peninsula, several translocation experiments were carried out from 1992 to 1993, comparing salmon from River Ulla (northwestern Spain) and River Shin (Scotland), the former being one of the rivers used in the restocking [12]. The results of these experiments, carried out in a hatchery and in an experimental channel located in northwest Spain, indicated that while salmon from River Shin reached a higher condition factor than salmon from River Ulla, mortality was significantly higher in salmon from River Shin during the early stages of development. These findings suggest that salmon from Scotland are not well adapted to the conditions of the northern Iberian Peninsula and may explain the failure of restocking with foreign salmon [13], which contrasts with the success of the supportive breeding program carried out with local populations since 1998 [14].

The fact that salmon populations located in different areas are genetically different is related to an innate preference to return to their river of origin, a behavior known as homing [15]. In line with this, a recent work by Jeffery et al. [16] studied the occurrence of regional differentiation in Atlantic salmon populations from both sides of the Atlantic using a panel of 96 SNPs. The authors were able to assign salmon populations to their areas of origin with high precision. Although the main cause of this strong differentiation is the reproductive behavior of the species, as gene flow is highly restricted by the tendency to return to the river of birth, other differentiating factors, such as local adaptation, may be involved [17,18]. Local adaptation can occur by divergent selection across environments, which favors the fixation of alleles that increase the fitness of a population in the environment it inhabits relative to other alleles favored in another population [19,20,21,22,23]. Consequently, the number of homozygous individuals for favorable alleles in each environment increases because of positive selection [24,25], thus contributing to population differentiation.

For example, in Atlantic salmon, the NADP-dependent malic enzyme malic (_m_MEP-2*) has been identified as a candidate for local adaptation, particularly for populations in the Iberian Peninsula, as its allelic frequencies vary at macro- and microgeographic scales in relation to freshwater temperatures and the at-sea age of returning adults [13,26,27].

The combined effect of natural selection and interpopulation isolation can produce a fingerprint in genomes. This effect of selection on the genomes of different populations of the same species can be detected by following at least two different methodologies [28,29], namely:

(1) Detection of atypically high values of differentiation (F_ST_ outliers) associated with particular markers/loci.
(2) Detection of unusual haplotypic patterns associated with a selective carryover effect, such as increased homozygosity in certain regions.

Regardless of the method, genome-wide fingerprinting simply provides indirect evidence of the possible selective effect, so it is necessary to annotate candidate genomic regions and address the consequent study of candidate genes potentially involved in the selective process. Identification of these selective signatures may reveal genomic regions of biological and commercial interest. In addition, understanding local adaptation could be important for interpreting how salmonid populations respond to habitat alterations, climate change and fisheries.

Therefore, the aim of this work is to compare the genomes of different populations from the Iberian Peninsula and Scotland to identify SNPs and candidate regions associated with high values of interpopulation differentiation and unusual haplotype patterns. The processes in which these genes are involved may explain the adaptive differences between populations and the failures of the restocking carried out during the 1980s.

## 2. Materials and Methods

**Figure 1:**
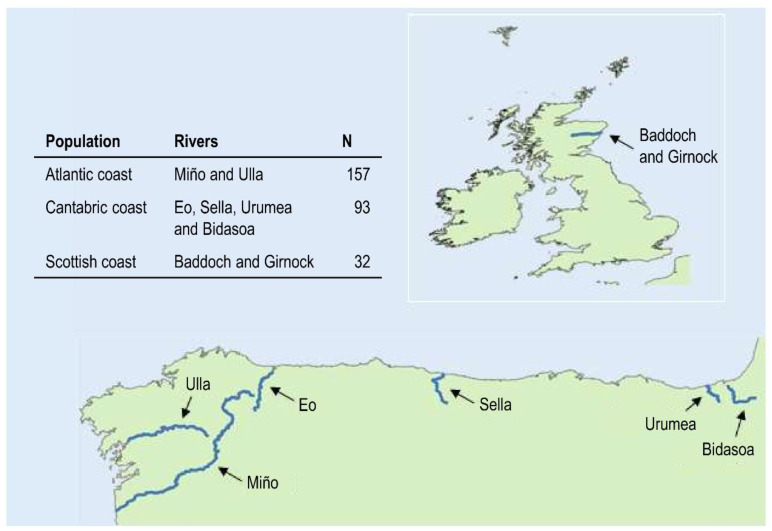
Origin and number of samples analysed: 250 individuals from six rivers in the Iberian Peninsula (Miño, Ulla, Eo, Sella, Urumea and Bidasoa), covering the entire distribution of salmon in its southern boundary, and 32 individuals from two Scottish rivers (Baddock and Girnock), tributaries of the Dee River.

A total of 282 returning adult individuals (165 females and 117 males) sampled between 2008 and 2009 were considered. To cover the entire distribution range of salmon in the Iberian Peninsula, 250 individuals from six Spanish rivers (Miño, Ulla, Eo, Sella, Urumea and Bidasoa) were analyzed. This included the Atlantic coast (77 females and 80 males) and the Cantabric coast (70 females and 23 males). The vast majority of the salmon analyzed came from recreational fishing. A few salmon came from sampling conducted by Atlantic salmon program management. For both the salmon caught by the rod (and dead) and those caught at the capture stations, the adipose fin is cut off and stored in ethanol by the management staff responsible for the salmon program. Additionally, 32 individuals from Scotland (18 females and 14 males) coming from two Scottish tributaries (Baddoch and Girnnock) of the Dee River (see Figure 1) were available for analysis. To identify the population structure, the samples were grouped using principal component analysis (PCA) with Adegenet [30] based on the Bayesian Information Criterion (BIC).

### 2.1. Genotyping Quality Analyses

To purify the genomic DNA from ethanol-preserved adipose fins, an NZY Tissue gDNA Isolation Kit (NZYtech) was used. Quantification and purity were assessed using a Nanodrop-1000 spectrophotometer. DNA samples were adjusted to a final concentration of 100 ng/μL and frozen until use. Morphological sex was confirmed by the presence/absence of the SDY intron gene (~200 bp) successfully amplified in males and absent in females, by using the primers SDY E1S1 and SDY E2AS4 [31,32]. Samples were genotyped using a 220 K Affymetrix genotyping array (Thermo Scientific) according to the manufacturer’s recommendations [33]. Genotypes from samples showing a dish quality control (DQC) < 0.82 or call rate < 0.97 were discarded. Only those SNPs classified as Poly High Resolution, with a call rate > 0.97, were used in our analysis. Unmapped SNPs and those with a minor allele frequency (MAF) < 0.01 were also removed (Table 1).

**Figure 2:**
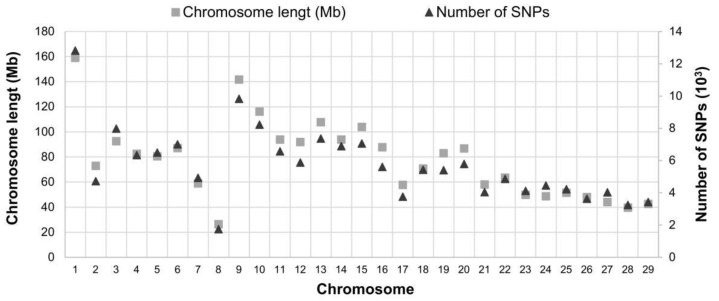
SNP distribution after quality control. A total of 165,774 SNPs remained allocated across the genome and were proportionally distributed according to the size of each chromosome.

After applying these filters, a total of 165,774 SNPs remained allocated across the genome and were proportionally distributed according to the size of each chromosome (Figure 2).

### 2.2. Selection Signatures and Gene Functional Annotation

To evaluate the occurrence of selection, we applied two methods based on the identification of FST outliers and three haplotype-based methods.

The first family of methods is one of the most common ways to detect local adaptation in non-model organisms. These methods rely on allelic frequencies, assuming that loci under selection will provide unusually high F_ST_ values [34,35,36]. The outlier-based methods were assessed using BayeScan v.2.1 [37] and the EOS test implemented in HacDivSel v.1.4 software [21]. BayeScan uses a Bayesian likelihood method to estimate the posterior probability of loci experiencing selection. We used default settings, but to protect from false positives, we increased prior odds for the neutral model to 5000 and considered as significant only SNPs detected with Bayes factor between 32 and 100 (log_10_ = 1.5–2.0), as these indicate a very strong or decisive signal of divergent selection [36,38]. On the other hand, EOS implements a two-step conservative heuristic strategy for detecting extreme F_ST_ outliers [21]. We performed the EOS analysis using the default parameters. Input files were converted to the correct format using PGDSpider v.2.1.1.2 [39].

Nevertheless, outliers’ strategy has some drawbacks and challenges that require the methods to be used with caution; for example, because the markers are considered independent, there is a loss of power when their number is large. In addition, the assumed null model can be wrong, leading to false positives if the population suffered demographic or historical events [38,40,41,42,43].

To overcome these drawbacks, we also applied haplotype-based methods for detecting diversifying selection and performed three different methods: (1) a cross-population extended haplotype homozygosity model (XP-EHH) [44], as implemented in selscan v. 1.1.0 [45], (2) the nvdF_ST_ test [21], and (3) a test for multiloci F_ST_ variance as implemented in SmileFinder [46]. Haplotypes were inferred from genotypes with fastPHASE software [47].

Although all three methods require haplotypes, the methodology for searching for selective patterns is different.

(1) XP-EHH. The cross-population extended haplotype homozygosity model is based on the inspection of patterns of linkage disequilibrium decay around selected loci and detects selection based on an excess of specific haplotypes in one of the populations. This method requires a linkage map. Since no map was available for our 220 K array, we used the physical distance.
(2) nvdF_ST_. The nvdF_ST_ statistic combines two measures: a normalized variance difference (nvd) and an F_ST_ index. The nvd measure divides the haplotypes into two sets for each candidate SNP: one set carrying the major allele for the SNP and the other set carrying the minor allele. Only SNPs shared by both populations were considered. A variance of mutational distances is computed within each set and a normalized difference between variances defines the statistic that will increase under selection. The F_ST_ measure takes advantage of the fact that if selection acts on a SNP pointed by a high nvd value, then the F_ST_ at that site will be higher when compared to the overall F_ST_ assuming equilibrium in the presence of migration. A resampling method is used to compute the *p*-value under the hypothesis of panmixia and the final candidate SNPs are those with highest nvd values that additionally reject panmixia [21]. We used different windows sizes (1000, 500, 250, 125 and 62) for computing nvd and considered as potential candidates the 1% of the SNPs with highest nvd, which were also significant for the F_ST_ test under any window size.
(3) SmileFinder. This method uses a resampling-based strategy to infer the significance of multiloci F_ST_ variance using sliding windows of haplotypes of increasing size. In this case, the highest values of variance indicate the presence of selection.

The gene content within genomic regions identified by candidate SNPs was explored using SalmoBase [48] and gene function annotated with the DAVID tool [49], using as reference and prioritizing by phylogenetic proximity, other salmonids, *Danio rerio, Xenopus laevis*, *Mus musculus* and *Homo sapiens.*

## 3. Results

### 3.1. Population Structure

**Figure 3:**
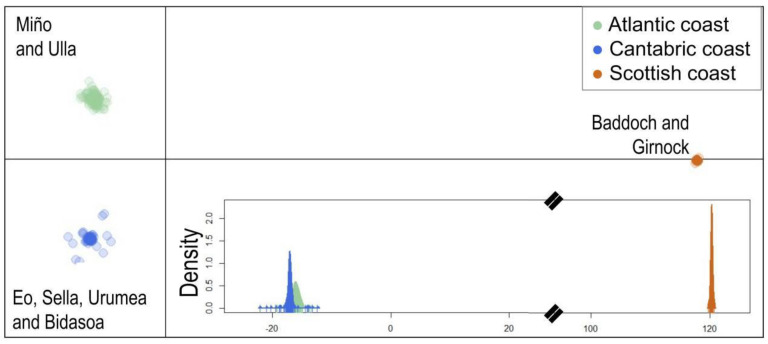
Samples were clustered through principal component analysis with Adegenet (Jombart 2008) based on the Bayesian Information Criterion (BIC). Three pools of data were clustered, listed as Atlantic (Miño and Ulla), Cantabric (Eo, Sella, Urumea and Bidasoa) and Scotland (Baddoch and Girnnock), with 157, 93 and 32 individuals, respectively.

Genotyped samples were classified into three groups after clustering with PCA according to samples from Scotland (Baddoch and Girnnock) and both slopes of the Iberian Peninsula: rivers from the Atlantic coast (Miño and Ulla) and from the Cantabric coast (Eo, Sella, Urumea and Bidasoa) (Figure 3). From now on, these three pools of data will be listed as Atlantic, Cantabric and Scotland, with 157, 93 and 32 individuals, respectively. Although 9% of individuals from Atlantic rivers were bound with Cantabric rivers, they were considered Atlantic data. This finding is probably related to the fact that during the 80s and until 1992, supportive breeding was performed in Galicia with an initial stock originating from different Galician rivers, including River Eo. However, since 1992, supportive breeding has been exclusively performed with individuals from the same river. 

### 3.2. Selection Signatures: Outlier Methods

Bayescan and EOS methods were applied for the comparison of population pairs. For Bayescan, we required a posterior probability of at least 0.9 (logBF ≥ 1.5) as evidence for local adaptation, but only the comparison between Atlantic and Scotland showed evidence of positive selection and only for two significant unannotated SNPs (ctg718000187612_7079_SGT and ctg7180001695888_3098_SCT) in chromosome *Ssa22* in a region of around 20 Mb. These same two SNPs were also detected by EOS, which found 142 significant SNPs in the comparison Cantabric–Scotland and 412 in Atlantic–Scotland. There were no significant SNPs in the comparison Atlantic versus Cantabric (Table 2).

**Figure 4:**
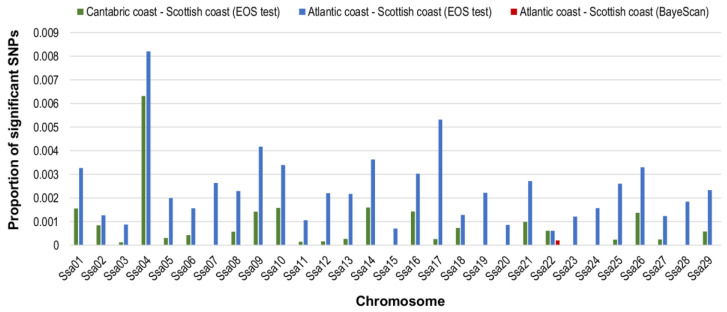
Proportion of significant SNPs of outliers, methods: BayeScan (log BF = 1.5) and EOS-HacDivSel (Atl-Can: Atlantic–Cantabric; Can-Scot: Cantabric–Scotland; Atl-Scot: Atlantic–Scotland).

The largest number of significant SNPs was found in chromosome *Ssa04*, for the comparison of Atlantic versus Scotland (Figure 4). In addition, additional regions were detected by EOS in both comparisons with Scotland. Notably, the 24.8–24.9 Mb region in *Ssa09* has previously been identified by several studies as carrying a strong diversifying selection signal among European but also North American Atlantic salmon populations [10,33,50,51].

### 3.3. Selection Signatures: Haplotype Methods

**Figure 5:**
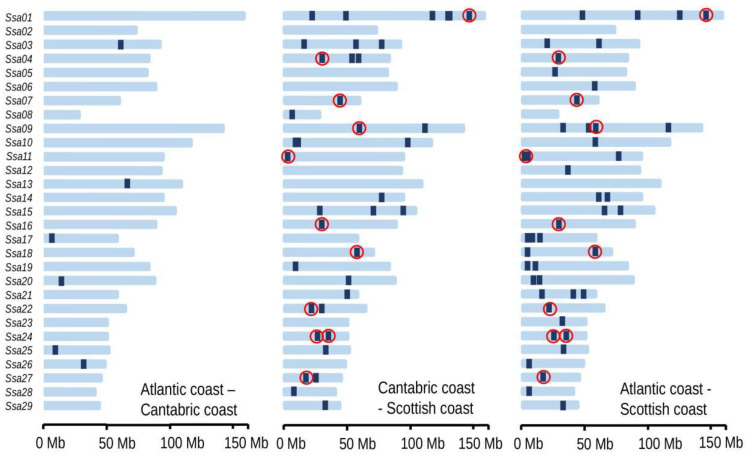
Per chromosome regions with significant SNPs obtained from at least two haplotype-based methods. Regions surrounded by a red circle indicate SNPs detected in both comparisons with Scotland.

The haplotype-based methods identified many more candidate SNPs, even for the Atlantic–Cantabric comparison (Table 2). Since the methods assayed imply different strategies to infer selection and it is a recommended practice to combine various approaches [52], we focused on those SNPs detected for at least two of the haplotype-based methods (Figure 5 and Table 3), whether they were also detected by the outlier-based methods. The complete list of these SNPs and the methods for which they were significant are given in Supplementary Tables S1 and S2.

Chromosomes *Ssa09*, *Ssa11* and *Ssa24* had the highest abundance of selection signatures detected by at least two haplotype-based methods, with 90 (Table S1 identifiers H136–H225), 95 (H241–H335) and 119 (H474–H592) SNPs, respectively (Table S1). There were 16 candidate SNPs for local adaptation between the Atlantic and Cantabric rivers, 506 for Atlantic–Scotland and 170 for Cantabric–Scotland (Table 3). Regarding the comparison with Scotland, 62 out of the 170 SNPs detected in Cantabric–Scotland were also detected in the Atlantic–Scotland pair. These common SNPs were located in different chromosomes: *Ssa01*, *04*, *07*, *09*, *11*, *16*, *18*, *22*, *24* and *27* (red circles in Figure 5).

Regarding the aforementioned region, 24.8–24.9 Mb of divergent selection in *Ssa09* was also identified in both comparisons with Scotland by the nvd_FST_ method, although not by selscan or SmileFinder. On the other hand, chromosome *Ssa22* had 13 significant SNPs detected for the Atlantic–Scotland comparison by both XP-EHH and nvdF_ST_ methods in a close region (1–2 Mb downstream), where BayeScan and EOS found two significant (non-annotated) SNPs (Figure 4).

Notably, 64 SNPs were significantly detected under all three methods for the Atlantic–Scotland comparison and 19 of these were for the Cantabric–Scotland one.

### 3.4. Gene Functional Annotation

The complete list of significant SNPs in one or two population comparisons and for two or three of the haplotype-based methods can be found in the supplementary Tables S1 and S2 and the genes containing these SNPs in Table S3.

### 3.5. Atlantic–Cantabric Comparison

As expected, there were fewer significant SNPs detected by the two haplotype methods in the Atlantic–Cantabric comparison than in either of the two pairwise comparisons with Scotland. These SNPs were located in chromosomes *Ssa03* (identifiers H040–H043 in Table S1), *Ssa13* (identifiers H339–H340 in Table S1), *Ssa17* (identifiers H408–H412 in Table S1), *Ssa20* (identifier H439 in Table S1), *Ssa25* (identifiers H593–H594 in Table S1) and *Ssa26* (identifiers H600–H601 in Table S1).

Notably, SNP H601 within gene Cyld, which was detected by nvd and SmileFinder (Table S2), has recently been associated with body weight in rainbow trout [53].

### 3.6. Comparison with Scotland: SNPs Significant for All Three Haplotype-Based Methods

There were 64 significant SNPs for all three haplotype-based methods. The GO (Gene Ontology) categories of the genes containing those SNPs correspond mainly to developmental and cellular processes. Among the annotated genes (Table 4), we found the *Protocadherin fat4* that pertains to the cadherin gene family and has been associated with amoebic gill disease resistance [54]; the glyoxalase 1 gene, glo1, which is important in many physiological processes and diseases and seems to increase its expression in response to stress [55]; MAM domain containing glycosylphosphatidylinositol anchor 1, *mdga1,* which is related to brain development [56], and has been previously identified as selective in the divergence between Norway populations of Atlantic salmon [57]; *WD repeat domain 43, wdr43* is related with lipid metabolism [58] and transcriptional response to contaminant exposure [59] and Zinc finger AN1-type domain 3, *zfand3* which is involved in sex determination and male germ cell maturation in the teleost Nile tilapia [60].

When the SNPs detected only by two haplotype-based methods were also considered, most of the genes identified were related to metabolic or cellular developmental processes, DNA transcription and anatomical structure. In *Ssa03*, the gene *tpr* is related to response to temperature changes in mice [61] affected by two SNPs in the comparison Cantabric–Scotland. Another polymorphism is close to *pgk*, a locus related to growth in the turbot in *Ssa04* [62]. Other genes involved with growth in Atlantic salmon are close to significant SNPs: *agrn* and *pomt1* in *Ssa15* (1,164,514 bp) and *Ssa20* (31,286 bp) [63]. In addition, other SNPs are near to *e2f4* in *Ssa10* and *fra10ac1* in *Ssa28* and are related to growth and late maturation in salmon [64].

### 3.7. Malic Enzyme

As the NADP-dependent malic enzyme-2 (_m_MEP-2*) has been reported to be a candidate for local adaptation in salmon from the Iberian Peninsula [27], there is a priori independent information about local adaptation effects related to that locus (LOC106586750), which is located at 39,878,908–39,909,791 bp in *Ss25* [65]. In this region, there is just one SNP (ctg7180001794010_7928_SCT) at 39,897,822 bp in chromosome *Ssa25* (id 3579 in Table S4). This malic SNP is surrounded by two other SNPs located at −11 kb and +15.6 kb (ids 3578 and 3580, Table S4). However, in chromosome *Ssa25,* there were only 6 SNPs detected as significant by at least two haplotype-based methods and these SNPs are located at 7,175,102 bp and 32,983,986 bp (Table S1), thus leaving out the region coding for _m_MEP-2*.

In a previous study using the same microarray and populations (from Spain), genomic linkage disequilibrium highly decreased for SNPs separated 1 Mb apart (reaching half of the maximum value between SNPs separated at 0.5 Mb [66]. In our case, if we consider a window size of 51 SNPs centered in the _m_MEP-2*gene, SNPs located upstream and downstream of the region correspond to distances of −0.23 Mb (SNP id 3554) at the left and +0.64 Mb (SNP id 3604) at the right. These distances are minimal because, contrary to the SNPs presented in Table S4—HacDivSel does not consider non-shared SNPs, which could increase the distance between the adjacent SNPs in a window of the given size.

Similarly, if we use a window of size 125 SNPs, we will have 62 SNPs on each side, implying a distance of at least 0.61 Mb at the left and 1.5 Mb at the right side from the malic SNP (ctg7180001794010_7928_SCT).

Therefore, we expect that if the malic SNP reflects a selective pattern affecting the variance of haplotype allelic classes, it could be detected under a window size of less than 50 SNPs or even 125 SNPs, since linkage disequilibrium is still appreciable up to 1 Mb away.

Moreover, considering a candidate SNP that has been previously identified, we can perform the test only for this SNP so that multiple testing corrections are not required. Unfortunately, because the distribution of the nvd statistic is not known, we still face the problem that the statistical significance of a SNP that we assigned to the 1% of candidates with the highest nvd is meaningless if we have only one candidate. Fortunately, we can still do a F_ST_ test and look for a more direct way of estimating statistical significance by comparing haplotype variances.

Therefore, we modified our program HacDivSel to introduce user-defined candidate SNPs and performed the analysis in a window of SNPs centered at the gene coding _m_MEP-2*. For each population, we compared the homogeneity of variances between the partitions having one or other malic allele, i.e., C or T at ctg7180001794010_7928_SCT SNP. These are the same variances compared by nvd, and we can use a robust test of variances (e.g., the composite test as defined in Ramsey and Ramsey 2007) to assess whether the variance for the major allele partition is significantly lower, which would indicate the presence of a positive selection effect in the population. We considered divergent selection when the comparison of variances was significant in at least one population and when both populations had a significantly high F_ST_ value (see Appendix A for details of the variance comparison). With this criterion, we analysed the SNP within the _m_MEP-2* region using windows of 25, 51 and 125 SNPs to study the possible pattern of divergent selection. The results are presented in Table 5.

The malic _m_MEP-2* SNP studied in this new method with the new version of HacDivSel software was clearly detected as a candidate for local adaptation in the three comparisons under the windows sizes assayed within 1 Mb. The window size of 125 SNPs was non-significant in the three comparisons, which probably means that the linkage disequilibrium decreased enough to make it difficult to detect the selection pattern under the given sample sizes.

Furthermore, the neighbors of _m_MEP-2* are only a few kb apart, which suggests that SNPs in the vicinity should still present a selective pattern, even in the Atlantic–Cantabric comparison (Table 6). In contrast, the SNPs in the extremes of the windows of size 50 and 125 SNPs should not have a selective pattern, at least not one caused by the _m_MEP-2* effect, with the exception of SNP ID 3704 (Table 6).

In addition, we estimated the false-positive rate (FPR) of the new method using simulated data. We note that, at least with the tested window sizes (from 25 to 400 SNPs), the method appears conservative, with an FPR below 5% (see Table A1 in Appendix A), supporting the reliability of our results.

## 4. Discussion

In this work, we performed a genome-wide scan using a high-density SNP array with the aim of investigating different patterns of selective variation in the Atlantic salmon genome to identify candidate loci that may be involved in the local adaptation of Atlantic salmon in the northern Iberian Peninsula, which is the southern limit of the distribution of this species.

We used different frequency-based (F_ST_ outliers) and haplotype-based selection detection methods and observed little overlap in the regions detected by the two types of strategies. The lack of overlap between frequency-based and haplotype-based methods has already been reported in other studies [67,68]. Regarding the use of outliers, most salmonid phenotypic traits are polygenic, such as growth, body size and fat content, among others [9], for which the effect of selection may involve a subtle change in allele frequency at several loci. This context makes it difficult to detect the selection effect using outlier-based methods that rely on a strong change in allele frequency at a few independent loci [21]. Therefore, as expected, haplotype-based methods have more power to detect recent signatures of selection [69]. We combined the results of three haplotype-based methods to identify 630 SNPs, 55% of which matched 116 genes in 25 out of 29 chromosomes (Tables S1–S3). The chromosomes containing more candidate genes were *Ssa24* (29 genes), *Ssa09* (13 genes), *Ssa11* (9 genes), *Ssa01* and *Ssa17* (8 genes) and *Ssa10* and *Ssa03* (7 and 6 genes, respectively). Genes potentially more affected by divergent selection were those resulting from the comparison between the peninsular slopes and Scotland, as expected, since the intensity of local adaptation appears to be correlated with the geographical distance between salmonid populations [9,57]. Our results support the idea that Atlantic salmon populations show significant genetic differences that may be the result of a combination of diversifying natural selection and the effect of gene flow and drift.

Even with the conservative threshold we used for outlier methods, coupled with the requirement of a positive signal in at least two haplotype-based methods, we detected quite a few signals and genes that could be involved in local adaptation processes. Similar to other genome-wide studies looking for local adaptation, several of the candidate genes we have found are distributed along different chromosomal regions and are related to cellular growth and lipid metabolic processes, among others.

In addition to the coincident SNPs for at least two haplotype-based methods, we found a well-known *Ssa09* haploblock associated with a strong signature of divergent selection among Atlantic salmon populations [10]. This region, located at 24.8–24.9 Mb, presented a strong signal in the comparisons between Scotland and the Atlantic and Cantabric populations for both the EOS and the nvdF_ST_ methods. The same region has been previously reported as a candidate for selection by several authors and contains the protein phosphatase 1a (*ppm1a*) gene and the SIX homeobox (*six6*), the latter related to circadian timing processes [10]. For the comparison between Atlantic and Scotland, nvdF_ST_ also detected SNPs within the region located at 77–78 Mb on *Ssa13*, corresponding to the SLC25A14 gene, which encodes a brain mitochondrial transporter protein and has also been found to diverge between European and North American populations [70]. Similarly, we found divergence in regions containing immune-related genes already identified in the literature as divergent in comparisons between northern and southern Norwegian populations [57].

### Malic Enzyme

The gene encoding the malic enzyme deserves special mention. As previously explained, this gene located at *Ss25* was specifically explored for candidate SNPs, since early electrophoretic studies on genetic variation in Atlantic salmon referred specifically to this gene [71]. Variation at _m_MEP-2* alleles (isozymes 100 and 125) presents strong correlations with environmental temperature, both within and among rivers, and associations with phenotypic performance [71]. Indeed, there is a latitudinal cline in which the frequency of the *125 allele of _m_MEP-2* is higher in northern European Atlantic salmon populations than in southern European populations. Moreover, in the Atlantic populations of the Iberian Peninsula, the frequency of the *125 allele is almost zero [72]. In fact, the failure of the restocking carried out in the Iberian Peninsula with salmon from Northern Europe was proven by the low frequency of the *125 allele in the Iberian populations after restocking [13]. 

However, with the genomic selection detection methods initially assayed, we could not detect any significant SNP within the region coding for _m_MEP-2*. However, this result was expected. First, the allele frequencies of the malic SNP were 0.02229 in the Atlantic sample, 0.134 in the Cantabric sample and 0.4219 in the Scotland sample, which implies that the frequencies of the three samples are in the same half of the frequency range. Second, since we are analyzing several thousand SNPs, multitest correction implies a loss of statistical power. Thus, it is expected that other more powerful signals will be more easily detected. Fortunately, after modification of the HacDivSel method to consider some candidates a priori, the malic _m_MEP-2* SNP, was clearly detected as a candidate for local adaptation.

Although the maladaptation of northern European salmon demonstrated by the garden experiment described by García de Leániz et al. [12] is not exclusively due to the malic enzyme, the role of this gene could be relevant, as suggested by the results obtained in the present work.

## 5. Conclusions

In this study, we provided additional evidence of local adaptation for north Spanish and Scottish populations of Atlantic salmon using genome-wide information. Defining the spatiotemporal scales over which adaptation operates is important from the point of view of management and conservation. Ignoring the existence of locally adapted populations may pose a greater risk to the conservation management of threatened populations [8]. We modified the program HacDivSel to introduce user-defined candidate SNPs and performed the analysis using a window of SNPs centered at a SNP within the NADP-dependent malic enzyme-2 (_m_MEP-2*), previously reported by independent studies as a candidate for local adaptation in salmon from the Iberian Peninsula. Interestingly, the corresponding SNP within the _m_MEP-2* region was consistent with a genomic pattern of divergent selection.

In summary, our results suggest that Spanish and Scottish populations may differ at the functional gene level due to local adaptation, which would explain the failure to restock Spanish rivers with Norway and Scottish individuals.

## Figures and Tables

**Table 1:** Quality control was performed on a 220,000 customized array.

Quality Filter	N° Removed SNP
Not mapped	1112
Low quality	47,432
MAF < 0.01	5679
Erratic genotypes	3
**Total analysed SNPs**	165,774

**Table 2:** Summary of significant SNPs in each comparison between studied populations after the application of different selection detection methods (Atl-Can: Atlantic–Cantabric; Can-Scot: Cantabric–Scotland; Atl-Scot: Atlantic–Scotland).

Statistic	Software	Number of Significant SNPs		
		**Atl-Can**	**Can-Scot**	**Atl-Scot**
F_ST_ outliers detection	HacDivSel (EOS test)	0	142	412
	BayeScan (logBF = 1.5)	0	0	2
Haplotype based methods	HacDivSel (nvdFST)	748	1504	2607
	SmileFinder	631	1346	2786
	selscan (XP-EHH)	201	1863	2880

**Table 3:** Number of significant SNPs in at least two haplotype-based methods for each comparison. Comparisons: Atl-Can: Atlantic–Cantabric; Atl-Scot: Atlantic–Scotland; Can-Scot: Cantabric–Scotland. Methods: X: XP-EHH; N: nvdFST; S: SmileFinder. Total: total number of different SNPs detected.

Comparison	Methods				
	**X-N**	**X-S**	**N-S**	**X-N-S**	**Total**
Atl-Can	0	4	14	0	16
Atl-Scot	210	275	147	64	506
Can-Scot	59	31	82	19	170

**Table 4:** Genes that include (or are located at less than 1000 kb) significant SNPs for the Atlantic–Scotland comparison detected by all three haplotype-based methods. SNPs were annotated using the online tool SalmoBase (https://salmobase.org/, accessed on 20 May 2021). The #SNPs refer to the number of all SNPs detected by all three methods in that chromosome, not only to those included in genes. The latter are indicated by SNP IDs. In parentheses, the Mb position in the corresponding chromosome.

Chromosome	# SNPs	SNP IDs	Gene (Mb)
*Ssa09*	23	H181–186, H188	fat4 (59)
*Ssa11*	37	H256 *, H259, H261–H264, H269, H275 *, H281 *, (H297–300) *, H304–310 *, H313, H324–325	wdr43 (1), trmt61b (1), atts-glupro (1), glo1 (1), zfand3 (1), mdga1 (1)
*Ssa24*	2	H479 *, H488 *	ppp6c (25), golga1 (25), rpl35 (25), ofml2a (25)
*Ssa27*	2	H615–616	-

*: was significant in both comparisons Atlantic–Scotland and Cantabric–Scotland.

**Table 5:** Homogeneity variance test for divergent selection from the new version of the HacDivSel program. Var test *p* value: The *p*-value of the within-population variance homogeneity *F* test. The within-population variance homogeneity test implies two comparisons, one for each population, and two *p* values are obtained. The minimum *p*-value obtained is given in the table.

Comparison	WINDOW SIZE	Var Test *p*-Value	F_ST_ *p*-Value	Divergence Significance Test
Atl-Can	25	4 × 10^−8^	0.014	*
	51	0.003	0.004	*
	125	0.164	0.002	ns
Can-Scot	25	0.011	0.038	*
	51	0.005	0.030	*
	125	0.653	0.024	ns
Atl-Scot	25	8 × 10^−10^	0	*
	51	0.021	0	*
	125	1	0	ns

*: Significant variance and F_ST_ *p*-values. ns: non-significant.

**Table 6:** Homogeneity variance test for divergent selection for the Atlantic–Cantabric comparison. ID: SNP ID in Table S4 (e.g., ID 3579 corresponds to SNP ctg7180001794010_7928_SCT in the region with the gene symbol LOC106586750 corresponding to _m_MEP-2*). Var test *p* value: minimum *p*-value of the two variance homogeneity *F* tests (one for each population).

ID	Window Size	Var Test *p*-Value	F_ST_ *p*-Value	Divergence Significance Test
3454	25	1	1	ns
3529	25	1	1	ns
3578	25	0.026	3 × 10^−9^	*
3579	25	4 × 10^−8^	0.002	*
3580	25	0.002	0.013	*
3629	25	1	1	ns
3704	25	2 × 10^−8^	0.008	*

*: Significant variance and F_ST_ *p*-values. ns: non-significant.

## Data Availability

Data is contained within the article and supplementary material.

## Supplementary Materials

The following supporting information can be downloaded at: https://www.mdpi.com/article/10.3390/biology11060933/s1. Gabianetal_SuppM_SNPs_array.xlsx—Subsequent to the quality control, a total of 165,774 SNPs remained from an 220 K Affymetrix genotyping array (Thermo Scientific) [33], customized from the 930 K XHD Ssal array (dbSNP accession numbers ss1867919552-ss1868858426). Gabianetal_SuppM_significant_SNPs.xlsx—Due to the low statistical power of the outlier tests, gene annotation was made from significant SNPs in at least two haplotype-based methods. Gabianetal_SuppM_gene_annotation.xlsx—Gene annotation was made from significant SNPs in at least two haplotype-based methods using the online tools SalmoBase [48] and [49]. Gabianetal_Appendix_MalicSelectionDetection.pdf. Supplementary Tables S1–S4.pdf. Table S1. Significant SNPs for at least two haplotype-based methods. ID: identifier. Chrom: chromosome. Atlantic and Cantabric—Scotland: indicates that both Atlantic–Scotland and Cantabric–Scotland comparisons were significant for the SNP. Table S2. Methods corresponding to the significant SNPs in Table S1 (significant for at least two haplotype-based methods). An asterisk indicates that the corresponding method detected the SNP as significant (SF: SmileFinder; sel: selscan). Table S3. Candidate genes, corresponding significant SNPs and gene ontology (GO). Table S4. SNPs in chromosome *Ssa25*.

## Appendix A

### Appendix A.1. Malic Enzyme

As explained in the main text, the NADP-dependent malic enzyme-2 (_m_MEP-2*) has been reported by independent studies as a candidate for local adaptation in salmon from the Iberian Peninsula [13,26,27]. There is *a priori* independent information about local adaptation effects related to that locus (LOC106586750), which is located at 39,878,908–39,909,791 bp in *Ss25* (Davidson et al., 2010). In our samples and after the quality controls, there is in this region just one SNP (ctg7180001794010_7928_SCT) at 39,897,822 bp in chromosome *Ssa25* (id 3579 in Table S4). This malic SNP is surrounded by two other SNPs that are −11 kb and +15.6 kb (ids 3578 and 3580, Table S4). However, after the analysis of chromosome *Ssa25* with 2 outlier and 3 haplotype-based methods (see main text), there were only 6 SNPs detected as significant by at least two haplotype-based methods. These SNPs were between positions 7,175,102 bp and 32,983,986 bp (Table S1), thus leaving out the region of _m_MEP-2*.

This is not so surprising; first, the sample allele frequencies of ctg7180001794010_7928_SCT were 0.02229 in the Atlantic sample, 0.134 in the Cantabric and 0.4219 in the Scotland sample, which implies the frequencies in the three samples are in the same half of the frequency range. In addition, since we are analyzing several thousand SNPs and all methods correct for multiple comparisons in some way, which implies a loss of statistical power, it is expected that other more powerful signals will be more easily detected.

### Appendix A.2. HAC Variance Method

The HAC variance method was developed by [73] and adapted in [21] for the two-population divergence scenario (*nvd* statistic). As with many other methods that explore the genome looking for characteristic selection footprints, there is a trade-off between power and false-positive control when applying a sliding window across the genome looking for candidate sites. However, if we had a priori information about a candidate locus, we could simply test that site, which would give us more statistical power since we would not have to correct for excess false positives caused by multiple testing. However, we still face the problem that the distribution of the nvd statistic is not known, and we cannot deduce as significant the 1% of candidates with the highest nvd if we have only one candidate. Therefore, we must look for a more direct way to compare haplotype variances and the simplest and most intuitive way to do this would be to test the homogeneity of variances between the two partitions.

Therefore, the HacDivSel program was modified to enter user-defined candidate SNPs and perform the analysis in a candidate-centered SNP window. For each population, we compared the homogeneity of variances between the partitions having one or the other candidate allele. These are the same variances compared by *nvd* and we can use a robust test of variances (see below) to assess whether the variance of the major allele partition is significantly lower, which could indicate the presence of a positive selection footprint in the population.

### Appendix A.3. Divergent Selection Detection Variance Test

#### Appendix A.3.1. Major Allele Reference Haplotype and Sample Partitions P_1_ and P_2_

Let us recall the concept of the major-allele-reference haplotype (MARH henceforth denoted by R) as an haplotype of length *L* + 1 constructed with the alleles at highest frequency (major alleles) for each of the *L* + 1 loci in a sample of *n* haplotypes. Now consider that we have *a priori* information about the selective effect of locus with SNP at position *x* (1 ≤ *x* ≤ *L* + 1). Let us call this the candidate position. We make a partition of the data in two groups, P_1_ including the haplotypes having the reference allele at the candidate position (i.e., having the same allele at position *x* as R) and P_2_ including the remaining haplotypes, i.e., having the non-reference allele at position *x*. Since the candidate allele is fixed in each partition, the haplotype length can henceforth be considered *L*.

We want to test the influence of the candidate allele on the surrounding positions. We expect that if the candidate position is selective, the distribution in P_1_ would be different from the distribution in P_2_ because the candidate major frequency allele (which defines P_1_) would produce a sweep effect of the surrounding ones. This sweep effect should translate into clustering of major alleles around the candidate allele. We do not expect a similar effect in P_2_. Thus, we want to test how the alleles are distributed around the candidate in P_1_ and compare them with P_2_. We can test this in several populations.

#### Appendix A.3.2. Haplotype Allelic Class (HAC)

We defined the mutational distance between any haplotype and R as the number of sites (SNPs) carrying an allele different from the one in R. Each group of haplotypes with the same mutational distance will constitute a haplotype allelic class (HAC). Therefore, let us note that as *h*_i_ the number of minor alleles each haplotype *i* carries, then every haplotype having the same *h* number belongs to the same HAC class. Thus, *h* is the sum of positions that have different alleles than in the reference haplotype. So, in a given haplotype for each position *k* ∈{1, *L*} we count the outcome *X_k_* = *I*(*s_k_* ≠ *r_k_*) were *s_k_* is the allele in the haplotype, *r_k_* is the allele in the reference and *I*(A) is the indicator variable taking value 1 if A is true and 0 otherwise. Therefore, the *h* value of a haplotype of length *L* is:

(A1) $$h=\sum_{k=1}^{L}X_{k}\mathrm{where}\;X_{k}=I\left(s_{k}\neq r_{k}\right)$$

Recall we have two partitions and for each partition the candidate position *x* is not included since we know that in P_1_ every haplotype *i* has by definition *X_ix_* = 0 and similarly *X_ix_* = 1 in P_2_. We are interested in studying the distribution of *X_ik_* in the remaining *L* positions. From the original sample of *n* haplotypes, each partition (P_1_ and P_2_) has *n*_1_ and *n*_2_ = *n* − *n*_1_ haplotypes of length *L*, respectively.

#### Appendix A.3.3. HAC Variance

For comparing the distribution of alleles around a candidate site in both samples P_1_ and P_2_ of haplotypes, we can perform a variance homogeneity test because if the candidate site is selective, we expect that a sweep effect would be translated into lower HAC variance *v*_1_ in P_1_ than variance *v*_2_ in P_2_ (*v*_1_ < *v*_2_). On the contrary, if the site is neutral, we expect similar variances or even *v*_1_ > *v*_2_ if the number of haplotypes in P_1_ is higher than in P_2_ [73]. Therefore, we want to test the neutral null hypothesis *v*_1_ ≥ *v*_2_ versus the alternative *v*_1_ < *v*_2_.

The unbiased variance of HAC values in a sample of *n* haplotypes is

(A2) $$v=\sum_{i=1}^{n}\frac{{\left(h_{i}-m\right)}^{2}}{\left(n-1\right)}=\frac{1}{n-1}\sum_{i=1}^{n}\left(h_{i}^{2}-m^{2}\right)$$

where *h*_i_ is the hac value for the haplotype *i* and $m=\sum_{i=1}^{n}\frac{h_{i}}{n}$ is the sample mean.

#### Appendix A.3.4. Skewness and Kurtosis

Because the distribution of HAC clasess is unknown, we should use statistics that are robust to deviations from normality, so we must identify the kind of deviation from normality, as different statistics perform better under different deviations. Therefore, we will measure both skewness and kurtosis in the haplotype samples.

There are various formalizations of the skewness and kurtosis concepts [74]. All these measures are based on the sample *r*th moment for a sample of size *n*

(A3) $$m_{r}=\frac{\sum_{i=1}^{n}{\left(x_{i}-\overset{´}{x}\right)}^{r}}{n}$$

Let

$$g_{1}=\frac{m_{3}}{m_{2}{}^{3/2}}\;\mathrm{and}\;g_{2}=\frac{m_{4}}{m_{2}{}^{2}}-3$$

then the sample skewness measures G_1_ and *b*_1_ are

$$G_{1}=\frac{\sqrt{n\left(n-1\right)}}{n-2}g_{1}\;\mathrm{and}\;b_{1}=\frac{m_{3}}{s³}={\left(\frac{n-1}{n}\right)}^{3/2}g_{1}$$

where *s* = *v*^1/2^ and the sample kurtosis G_2_ and *b*_2_ are

$$G_{2}=\frac{n-1}{\left(n-2\right)\left(n-3\right)}\left\{\left(n+1\right)g_{2}+6\right\}\;\mathrm{and}\;b_{2}=\frac{m_{4}}{s⁴}-3={\left(\frac{n-1}{n}\right)}^{2}\frac{m_{4}}{m_{2}{}^{2}}-3$$

G_1_ and G_2_ formulations for skewness and kurtosis respectively, are recommended for small samples from non-normal distributions as they have smaller mean-squared error than other measures [74]. By other side, *b*_1_ and *b*_2_ measures work well for normally distributed data while *g*_1_ and *g*_2_ measures are the basis of the D’Agostino normality test [75]. The variances of these measures are related, and normally distributed transformations are available to perform skewness and kurtosis tests [75].

#### Appendix A.3.5. Homogeneity Variance Tests

There are a number of procedures for testing homogeneity of variances in two samples; however, for some of them, Type I error control is extremely sensitive to kurtosis [76]. Two robust tests of variance are BF [77] and OB [78]. Based on both, a composite method has been proposed that applies OB when a sample is detected as platykurtic and BF otherwise [76].

BF and OB tests were based on the one-way ANOVA statistic.

(A4) $$W\left(z\right)=\frac{\sum_{i=1}^{K}n_{i}{\left(\overset{´}{z_{i}}-\overset{´}{\overset{´}{z}}\right)}^{2}/\left(K-1\right)}{\sum_{i}^{K}\sum_{j=1}^{n_{i}}{\left(z_{ij}-\overset{´}{z_{i}}\right)}^{2}/\sum_{i}\left(n_{i}-1\right)}$$

where

$$\overset{´}{z_{i}}=\sum_{j=1}^{n_{i}}z_{ij}/n_{i}\;\mathrm{and}\;\overset{´}{\overset{´}{z}}=\frac{\sum_{i=1}^{K}\sum_{j=1}^{n_{i}}z_{ij}}{\sum_{i=1}^{K}n_{i}}$$

Brown-Forsythe (BF) test

Depending on the definition of the *z* values, we have different statistics, for example if zij=|xij−xi|,*W*(z) is the Levene test with *K* − 1 (*K* is the number of groups, 2 in our case) and $\underset{i}{\sum }\left(n_{i}-1\right)$ degrees of freedom. Alternatively, if we define *zij* = |*xij* − ~*xi*| where ~*xi* is the sample median; then we have the BF test [77].

O’Brien (OB) test

Define $y_{ij}={\left(x_{ij}-\overset{´}{x_{i}}\right)}^{2}$ and

$$r_{ij}=\frac{\left(n_{i}-3/2\right)n_{i}y_{ij}-v_{i}\left(n_{i}-1\right)/2}{\left(n_{i}-1\right)\left(n_{i}-2\right)}$$

where *v_i_* is the sample variance of group *i*. Given the transformation *r_ij_* note that for each group, the mean of the *r* transformed values is equal to the sample variance of that group [78],

$$\overset{´}{r_{i}}=\sum_{j=1}^{n_{i}}r_{ij}/n_{i}=\sum_{j=1}^{n_{i}}\left(x_{ij}-\overset{´}{x_{i}}\right)2/\left(n_{i}-1\right)=v_{i}$$

The OB test takes *z_ij_* = *r_ij_* and then applies *W*(*r*) from Equation (A4)

$$W\left(r\right)=\frac{\sum_{i=1}^{K}n_{i}{\left(v_{i}-\overset{´}{\overset{´}{r}}\right)}^{2}/\left(K-1\right)}{\sum_{i}^{K}\sum_{j=1}^{n_{i}}{\left(r_{ij}-v_{i}\right)}^{2}/\sum_{i}\left(n_{i}-1\right)}$$

Given BF or OB, the corresponding *W* statistic is compared with the *F* critical value with *K* − 1 and $\underset{i}{\sum }\left(n_{i}-1\right)$ degrees of freedom in the numerator and denominator, respectively.

### Appendix A.4. Algorithm for Divergent Selection Variance Test

The procedure for testing divergent selections in a set of populations is as follows.

1. Decide a candidate SNP *x* and the number of markers *L (*window size).
2. For each population, *i* define its major-allele-reference haplotype *R_i_*, and perform sample partitions P_*i*1_ and P_*i*2_ with the haplotypes carrying the major or minor allele of *x*, respectively.
3. Compute sample variances *v*_*i*1_ for the P_*i*1_ partition of population *i* and compute *v*_2_ as the sample variance of the accumulated HAC values of the P_2_ partitions for all populations. The reason for accumulating HAC values over the different populations for the second partition is that the sample size is smaller in these partitions and the allele distribution is expected to be random (no sweep) around the candidate site.
4. If *v*_*i*1_ ≥ *v*_2_ we conclude that there is no selection at the candidate site and if there are more populations, return to the second step to analyze the next population. Otherwise, if *v*_*i*1_ < *v*_2_, we continue with step 5.
5. For each partition, test skewness and kurtosis, i.e., test the null of no skewness (*g*_1_ = 0) and if kurtosis is not platykurtic (*g*_2_ ≥ 0, D’Agostino test).
6. If data for both partitions are normally distributed, an *F* test is performed. If data is marginally normal (one partition is normal and the other has a *p* value above α/2) then a Levene test is performed. Otherwise, we use the composite test BF-OB that applies OB if any of the partitions is platykurtic and BF otherwise. If homogeneity of variances is rejected, we conclude that candidate site *x* is selective in population *i*. For the computation of skewness and kurtosis using the D’Agostino test, a sample size of at least 8 is required. If the sample size is below this value, the homogeneity test will be the OB, which is more robust under a low sample size.
7. Repeat the above steps 1–6 for the next population. Divergent selection between populations *i* and *j* at site *x* can be concluded if *x* was selective in at least one population and the *F_ST_* index that compares the *F_ST_* for site *x* with the overall *F_ST_* is significant. The significance of the *F_ST_* index was computed by resampling, as in [21].

### Appendix A.5. Simulations

To study the control of type I error in the method, we use publicly available data from simulations in [21]. Specifically, the files indicated in Table A1 correspond to two populations of 1000 facultative hermaphrodites simulated during 10,000 generations. The mating was random within each population. The between-population migration was *N*m = 10. Each individual consisted of a diploid chromosome of length 106 biallelic sites. Two different population mutation rates per genome were assayed (θ = 4 *N*µ = {12, 60}) and four different population recombination rates (ρ = 4 *Nr* = {0, 4, 12, 60}). Natural selection was not simulated, so all scenarios were neutral. At the end of each run, 50 haplotypes were sampled from each population.

#### Analysis of the Simulated Data

We assumed as a candidate a SNP located in the center of the SNPs available in the genome. Different window sizes were used, depending on the number of segregating sites. In general, the minimum window size was 25, and the maximum was the total number of segregating sites. The results indicated that at least for a significance level of 0.05, the test was conservative, and the false positive rate was below 0.05 (Table A1).

**Table A1:** False positive rate (FPR) for the divergent selection detection variance test using simulated neutral evolution data available from Carvajal-Rodríguez, 2017 [21]. The genome size was 1 Mb. Population size *N* = 1000. Number of generations *T* = 10,000. Population mutation rate θ = 4 *N*µ. Population recombination rate ρ = 4 *Nr*. The candidate position is in the middle of the window. The total number of replicates for each case was 1000. FPR = 100×number of replicates with significant test/1000. MAF = 1%. Significance level α = 0.05.

File Name	θ	ρ	Mean Window Size	FPR
C4	12	0	25	1%
C4	12	0	87	1%
C5	12	4	25	2%
C5	12	4	87	1%
C6	12	12	25	2%
C6	12	12	86	1%
C16	60	0	25	1%
C16	60	0	51	1%
C16	60	0	125	2%
C16	60	0	436	1%
C17	60	4	25	2%
C17	60	4	51	2%
C17	60	4	125	2%
C17	60	4	429	2%
C18	60	60	25	2%
C18	60	60	51	2%
C18	60	60	125	2%
C18	60	60	431	1%
