# Supplementary material for: Detecting Local Adaptation between North and South European Atlantic Salmon Populations
Source: Biology (Basel). 2022 Jun 19;11(6):933. doi: 10.3390/biology11060933 (PMC9219887; doi:10.3390/biology11060933)
Supplement: Supplementary file 1 [file biology-11-00933-s001.zip › Supp_TablesS1-S4.pdf]

DETECTING LOCAL ADAPTATION BETWEEN NORTH AND SOUTH EUROPEAN ATLANTIC SALMON POPULATIONS

María Gabián[1], Paloma Morán[1], María Saura[2], Antonio Carvajal-Rodríguez[1*]

[1]Centro de Investigación Mariña (CIM), Departamento de Bioquímica, Genética e Inmunología, Universidade de Vigo, Vigo, Spain

[2]Departamento de Mejora Genética Animal, Instituto Nacional de Investigación y Tecnología Agraria y Alimentaria (INIA-CSIC), Madrid, Spain

* Corresponding author

E-mail: acraaj@uvigo.es

Supplementary tables

Table S1. Significant SNPs for at least two haplotype-based methods. ID: identifier. Chrom: chromosome. Atlantic & Cantabric - Scotland: indicates that both Atlantic-Scotland and Cantabric-Scotland comparisons were significant for the SNP.

ID	Chrom	SNP	Position	Comparison
H001	*Ssa01*	ctg7180001694954_3432_SAG	20,820,778	Cantabric - Scotland
H002	*Ssa01*	ctg7180001855312_1515_SAC	20,843,008	Cantabric - Scotland
H003	*Ssa01*	ctg7180001855312_1346_SCT	20,843,177	Cantabric - Scotland
H004	*Ssa01*	ctg7180001855312_531_SCT	20,843,992	Cantabric - Scotland
H005	*Ssa01*	ctg7180001307194_7323_SAC	20,846,500	Cantabric - Scotland
H006	*Ssa01*	ctg7180001307194_6055_SAG	20,847,768	Cantabric - Scotland
H007	*Ssa01*	ctg7180001307194_5625_SCT	20,848,198	Cantabric - Scotland
H008	*Ssa01*	ctg7180001307194_5603_SCT	20,848,220	Cantabric - Scotland
H009	*Ssa01*	ctg7180001307194_4028	20,848,661	Cantabric - Scotland
H010	*Ssa01*	ctg7180001851537_3635_SAG	48,285,188	Cantabric - Scotland
H011	*Ssa01*	ctg7180001851537_8799_SAG	48,290,352	Cantabric - Scotland
H012	*Ssa01*	ctg7180001756285_1481_SAC	48,568,283	Atlantic - Scotland
H013	*Ssa01*	ctg7180001912826_424_SCT	48,631,908	Atlantic - Scotland
H014	*Ssa01*	ctg7180001909402_1529_SCT	48,634,126	Atlantic - Scotland
H015	*Ssa01*	ctg7180001909402_6720_SAG	48,639,317	Atlantic - Scotland
H016	*Ssa01*	ctg7180001909402_7221_SAC	48,639,818	Atlantic - Scotland
H017	*Ssa01*	ctg7180001367958_2782_SGT	48,642,864	Atlantic - Scotland

H018	*Ssa01*	ctg7180001875704_3999_SAG	93,514,867	Atlantic - Scotland
H019	*Ssa01*	ctg7180001875704_3788_SCT	93,515,078	Atlantic - Scotland
H020	*Ssa01*	ctg7180001875704_1783_SAG	93,517,083	Atlantic - Scotland
H021	*Ssa01*	ctg7180001904381_6727_SAC	93,536,713	Atlantic - Scotland
H022	*Ssa01*	ctg7180001904381_3866_SAG	93,539,574	Atlantic - Scotland
H023	*Ssa01*	ctg7180001865646_9569_SAG	93,553,517	Atlantic - Scotland
H024	*Ssa01*	ctg7180001844605_1872_SCT	118,396,349	Cantabric - Scotland
H025	*Ssa01*	ctg7180001813606_1744_SAC	127,089,824	Atlantic - Scotland
H026	*Ssa01*	ctg7180001625892_282_SGT	130,368,540	Cantabric - Scotland
H027	*Ssa01*	ctg7180001490210_13171_SAG	130,590,068	Cantabric - Scotland
H028	*Ssa01*	ctg7180001490210_11991_SGT	130,591,248	Cantabric - Scotland
H029	*Ssa01*	ctg7180001530551_6000_SAG	147,026,514	Atlantic& Cantabric - Scotland
H030	*Ssa01*	ctg7180001933038_21577_SCT	147,055,027	Atlantic - Scotland
H031	*Ssa01*	ctg7180001933038_27729_SAG	147,061,179	Atlantic - Scotland
H032	*Ssa01*	ctg7180001394286_776_SAG	147,073,967	Atlantic - Scotland
H033	*Ssa03*	ctg7180001838466_446_SGT	15,060,009	Cantabric - Scotland
H034	*Ssa03*	ctg7180001838466_3126_SAG	15,062,689	Cantabric - Scotland
H035	*Ssa03*	ctg7180001838466_3184_SCT	15,062,747	Cantabric - Scotland
H036	*Ssa03*	ctg7180001838466_4593_SAG	15,064,156	Cantabric - Scotland
H037	*Ssa03*	ctg7180001537731_370_SAG	20,144,561	Atlantic - Scotland
H038	*Ssa03*	ctg7180001865890_80_SAC	56,586,907	Cantabric - Scotland
H039	*Ssa03*	ctg7180001681735_1954_SCT	56,928,284	Cantabric - Scotland
H040	*Ssa03*	ctg7180001653564_9841_SGT	60,822,757	Atlantic - Cantabric
H041	*Ssa03*	ctg7180001653564_9258_SAG	60,823,340	Atlantic - Cantabric
H042	*Ssa03*	ctg7180001653564_9122_SAC	60,823,476	Atlantic - Cantabric
H043	*Ssa03*	ctg7180001653564_3546_SAG	60,829,052	Atlantic - Cantabric
H044	*Ssa03*	ctg7180001911562_17971_SCT	61,627,534	Atlantic - Scotland
H045	*Ssa03*	ctg7180001911562_34059_SAG	61,646,162	Atlantic - Scotland
H046	*Ssa03*	ctg7180001598832_173_SCT	61,649,139	Atlantic - Scotland
H047	*Ssa03*	ctg7180001727325_1273_SCT	61,680,439	Atlantic - Scotland
H048	*Ssa03*	ctg7180001810694_6823_SCT	61,682,427	Atlantic - Scotland
H049	*Ssa03*	ctg7180001810694_1839_SAG	61,687,412	Atlantic - Scotland
H050	*Ssa03*	ctg7180001810694_1828_SCT	61,687,423	Atlantic - Scotland
H051	*Ssa03*	ctg7180001895507_1301_SAC	77,261,393	Cantabric - Scotland
H052	*Ssa04*	ctg7180001387971_2101_SAG	28,448,343	Atlantic - Scotland
H053	*Ssa04*	ctg7180001387971_5254_SCT	28,451,496	Atlantic - Scotland
H054	*Ssa04*	ctg7180001387971_5642_SGT	28,451,884	Atlantic - Scotland
H055	*Ssa04*	ctg7180001276135_397_SAG	28,602,887	Atlantic - Scotland
H056	*Ssa04*	ctg7180001917074_5769_SAC	28,610,395	Atlantic - Scotland
H057	*Ssa04*	ctg7180001917074_6404_SCT	28,611,030	Atlantic - Scotland
H058	*Ssa04*	ctg7180001801401_7471_SAG	28,618,235	Atlantic - Scotland
H059	*Ssa04*	ctg7180001568011_222_SAG	28,638,139	Atlantic - Scotland
H060	*Ssa04*	ctg7180001568011_9272_SAC	28,647,189	Atlantic& Cantabric - Scotland
H061	*Ssa04*	ctg7180001568011_9346_SCT	28,647,263	Atlantic - Scotland
H062	*Ssa04*	ctg7180001568011_9651_SCT	28,647,568	Atlantic - Scotland
H063	*Ssa04*	ctg7180001568011_14270_SCT	28,652,185	Atlantic - Scotland
H064	*Ssa04*	ctg7180001920093_1352_SGT	28,755,770	Atlantic - Scotland
H065	*Ssa04*	ctg7180001865186_142_SAC	28,757,527	Atlantic - Scotland
H066	*Ssa04*	ctg7180001909538_7926_SAC	52,559,799	Cantabric - Scotland

H067	*Ssa04*	ctg7180001795621_4418_SCT	52,585,875	Cantabric - Scotland
H068	*Ssa04*	ctg7180001633401_1478_SGT	52,605,677	Cantabric - Scotland
H069	*Ssa04*	ctg7180001893589_2329_SCT	52,632,276	Cantabric - Scotland
H070	*Ssa04*	ctg7180001893589_2013_SAG	52,632,592	Cantabric - Scotland
H071	*Ssa04*	ctg7180001816477_4904_SCT	52,638,293	Cantabric - Scotland
H072	*Ssa04*	ctg7180001892043_5580_SCT	52,652,296	Cantabric - Scotland
H073	*Ssa04*	ctg7180001890283_8200_SGT	52,666,275	Cantabric - Scotland
H074	*Ssa04*	ctg7180001890283_5650_SAC	52,668,825	Cantabric - Scotland
H075	*Ssa04*	ctg7180001824917_5648_SAG	52,678,112	Cantabric - Scotland
H076	*Ssa04*	ctg7180001824917_5232_SGT	52,678,528	Cantabric - Scotland
H077	*Ssa04*	ctg7180001824917_2581_SAC	52,681,177	Cantabric - Scotland
H078	*Ssa04*	ctg7180001890282_769_SAC	52,684,592	Cantabric - Scotland
H079	*Ssa04*	ctg7180001853453_2406_SAG	52,704,419	Cantabric - Scotland
H080	*Ssa04*	ctg7180001853453_6019_SAC	52,708,032	Cantabric - Scotland
H081	*Ssa04*	ctg7180001853453_11003_SAC	52,713,016	Cantabric - Scotland
H082	*Ssa04*	ctg7180001918676_10351_SAG	52,726,215	Cantabric - Scotland
H083	*Ssa04*	ctg7180001918676_9172_SGT	52,727,394	Cantabric - Scotland
H084	*Ssa04*	ctg7180001458299_1305_SGT	52,731,200	Cantabric - Scotland
H085	*Ssa04*	ctg7180001753634_1416_SAC	52,736,270	Cantabric - Scotland
H086	*Ssa04*	ctg7180001837716_2396_SGT	52,749,831	Cantabric - Scotland
H087	*Ssa04*	ctg7180001837716_3457_SAG	52,750,892	Cantabric - Scotland
H088	*Ssa04*	ctg7180001680513_4742_SCT	52,766,975	Cantabric - Scotland
H089	*Ssa04*	ctg7180001680513_4824_SCT	52,767,091	Cantabric - Scotland
H090	*Ssa04*	ctg7180001431941_7734_SCT	52,775,443	Cantabric - Scotland
H091	*Ssa04*	ctg7180001794891_10015_SCT	52,849,579	Cantabric - Scotland
H092	*Ssa04*	ctg7180001794891_6775_SAG	52,852,819	Cantabric - Scotland
H093	*Ssa04*	ctg7180001794891_6036_SCT	52,853,558	Cantabric - Scotland
H094	*Ssa04*	ctg7180001786383_967_SAG	52,919,870	Cantabric - Scotland
H095	*Ssa04*	ctg7180001858457_3878_SAC	57,316,242	Cantabric - Scotland
H096	*Ssa05*	ctg7180001872301_13800_SAC	25,962,680	Atlantic - Scotland
H097	*Ssa05*	ctg7180001872301_13812_SCT	25,962,692	Atlantic - Scotland
H098	*Ssa05*	ctg7180001872301_13976_SCT	25,962,856	Atlantic - Scotland
H099	*Ssa05*	ctg7180001872301_15824_SGT	25,964,704	Atlantic - Scotland
H100	*Ssa05*	ctg7180001872301_18965_SAC	25,967,845	Atlantic - Scotland
H101	*Ssa05*	ctg7180001872301_19123_SAG	25,968,003	Atlantic - Scotland
H102	*Ssa05*	ctg7180001872301_19404_SGT	25,968,284	Atlantic - Scotland
H103	*Ssa05*	ctg7180001872301_19936_SAC	25,968,816	Atlantic - Scotland
H104	*Ssa05*	ctg7180001574774_983_SAC	26,081,452	Atlantic - Scotland
H105	*Ssa06*	ctg7180001865390_52593_SAG	57,524,001	Atlantic - Scotland
H106	*Ssa06*	ctg7180001865390_52072_SAC	57,524,522	Atlantic - Scotland
H107	*Ssa06*	ctg7180001865390_6152_SCT	57,571,314	Atlantic - Scotland
H108	*Ssa06*	ctg7180001865390_2479_SAC	57,574,987	Atlantic - Scotland
H109	*Ssa06*	ctg7180001825142_991_SAG	57,586,584	Atlantic - Scotland
H110	*Ssa06*	ctg7180001825142_544_SGT	57,587,031	Atlantic - Scotland
H111	*Ssa06*	ctg7180001935321_1356_SAC	57,591,328	Atlantic - Scotland
H112	*Ssa06*	ctg7180001935321_4281_SGT	57,594,253	Atlantic - Scotland
H113	*Ssa06*	ctg7180001935321_5752_SAG	57,595,724	Atlantic - Scotland
H114	*Ssa06*	ctg7180001935321_5802_SCT	57,595,774	Atlantic - Scotland
H115	*Ssa06*	ctg7180001935321_6272_SGT	57,596,244	Atlantic - Scotland

H116	*Ssa07*	ctg7180001871459_3867_SGT	42,784,225	Atlantic - Scotland
H117	*Ssa07*	ctg7180001893245_2087_SAG	43,185,946	Atlantic& Cantabric - Scotland
H118	*Ssa07*	ctg7180001672219_4481_SAC	43,264,460	Atlantic - Scotland
H119	*Ssa07*	ctg7180001672219_5520_SCT	43,265,499	Atlantic - Scotland
H120	*Ssa07*	ctg7180001672219_5995_SAC	43,265,974	Atlantic - Scotland
H121	*Ssa07*	ctg7180001672219_6240_SGT	43,266,219	Atlantic - Scotland
H122	*Ssa07*	ctg7180001672219_6301_SAC	43,266,280	Atlantic - Scotland
H123	*Ssa07*	ctg7180001672219_6390_SCT	43,266,369	Atlantic - Scotland
H124	*Ssa07*	ctg7180001672219_6510_SAC	43,266,489	Atlantic& Cantabric - Scotland
H125	*Ssa07*	ctg7180001793746_8564_SAC	43,371,429	Atlantic - Scotland
H126	*Ssa07*	ctg7180001826079_3087_SGT	43,405,455	Atlantic - Scotland
H127	*Ssa07*	ctg7180001805184_4596_SCT	43,498,942	Atlantic - Scotland
H128	*Ssa07*	ctg7180001805184_4532_SCT	43,499,006	Atlantic - Scotland
H129	*Ssa07*	ctg7180001805184_4434_SAC	43,499,104	Atlantic - Scotland
H130	*Ssa07*	ctg7180001838980_4508_SAG	43,508,161	Atlantic - Scotland
H131	*Ssa07*	ctg7180001838980_4339_SAG	43,508,330	Atlantic - Scotland
H132	*Ssa07*	ctg7180001624097_4181_SGT	43,592,260	Atlantic - Scotland
H133	*Ssa07*	ctg7180001915467_2618_SGT	43,682,168	Atlantic - Scotland
H134	*Ssa07*	ctg7180001923514_7991_SAC	43,760,760	Cantabric - Scotland
H135	*Ssa08*	ctg7180001885288_8610_SAC	5,949,717	Cantabric - Scotland
H136	*Ssa09*	ctg7180001337954_175_SAG	32,533,654	Atlantic - Scotland
H137	*Ssa09*	ctg7180001703712_449_SAC	32,606,736	Atlantic - Scotland
H138	*Ssa09*	ctg7180001619872_240_SCT	53,067,170	Atlantic - Scotland
H139	*Ssa09*	ctg7180001887817_4712_SAG	53,483,285	Atlantic - Scotland
H140	*Ssa09*	ctg7180001794200_7623_SAG	53,664,357	Atlantic - Scotland
H141	*Ssa09*	ctg7180001794200_7660_SAG	53,664,394	Atlantic - Scotland
H142	*Ssa09*	ctg7180001740047_2126_SCT	53,677,948	Atlantic - Scotland
H143	*Ssa09*	ctg7180001720194_1426_SCT	58,184,143	Atlantic - Scotland
H144	*Ssa09*	ctg7180001848662_1098_SCT	58,193,464	Atlantic - Scotland
H145	*Ssa09*	ctg7180001848663_157_SAG	58,195,011	Atlantic - Scotland
H146	*Ssa09*	ctg7180001848663_584_SAG	58,195,438	Atlantic - Scotland
H147	*Ssa09*	ctg7180001848663_1122_SAG	58,195,976	Atlantic - Scotland
H148	*Ssa09*	ctg7180001848663_10213_SCT	58,205,067	Atlantic - Scotland
H149	*Ssa09*	ctg7180001847355_9337_SAG	58,223,389	Atlantic - Scotland
H150	*Ssa09*	ctg7180001312649_14961_SAC	58,248,402	Atlantic - Scotland
H151	*Ssa09*	ctg7180001312649_453_SAG	58,262,909	Atlantic - Scotland
H152	*Ssa09*	ctg7180001887843_972_SGT	58,266,637	Atlantic - Scotland
H153	*Ssa09*	ctg7180001887843_2898_SGT	58,268,563	Atlantic - Scotland
H154	*Ssa09*	ctg7180001838944_8427_SAC	58,375,235	Atlantic - Scotland
H155	*Ssa09*	ctg7180001865919_2204_SAG	58,389,661	Atlantic - Scotland
H156	*Ssa09*	ctg7180001590636_1895_SCT	58,430,105	Atlantic - Scotland
H157	*Ssa09*	ctg7180001402062_4911_SAG	58,452,828	Atlantic - Scotland
H158	*Ssa09*	ctg7180001830587_2964_SAC	58,482,665	Atlantic - Scotland
H159	*Ssa09*	ctg7180001884548_9438_SAG	58,500,020	Atlantic - Scotland
H160	*Ssa09*	ctg7180001880756_664_SAG	58,847,162	Atlantic - Scotland
H161	*Ssa09*	ctg7180001855470_4117_SAG	58,869,843	Atlantic - Scotland
H162	*Ssa09*	ctg7180001855471_4478_SGT	58,876,238	Atlantic - Scotland
H163	*Ssa09*	ctg7180001598309_1102_SCT	58,893,732	Atlantic - Scotland
H164	*Ssa09*	ctg7180001865182_2554_SAC	58,924,618	Atlantic - Scotland

H165	*Ssa09*	ctg7180001928730_8743_SCT	58,979,811	Atlantic - Scotland
H166	*Ssa09*	ctg7180001928730_11106_SGT	58,982,174	Atlantic - Scotland
H167	*Ssa09*	ctg7180001928730_11750_SAC	58,982,818	Atlantic - Scotland
H168	*Ssa09*	ctg7180001824427_4368_SGT	59,016,443	Atlantic - Scotland
H169	*Ssa09*	ctg7180001824427_1611_SCT	59,019,200	Atlantic - Scotland
H170	*Ssa09*	ctg7180001914278_2855_SAG	59,100,095	Atlantic - Scotland
H171	*Ssa09*	ctg7180001902377_817_SGT	59,124,136	Atlantic - Scotland
H172	*Ssa09*	ctg7180001902377_742_SAC	59,124,211	Atlantic - Scotland
H173	*Ssa09*	ctg7180001910145_3004_SCT	59,167,618	Atlantic - Scotland
H174	*Ssa09*	ctg7180001910145_3738_SGT	59,168,352	Atlantic - Scotland
H175	*Ssa09*	ctg7180001910145_14822_SCT	59,179,436	Atlantic - Scotland
H176	*Ssa09*	ctg7180001910145_15746_SAG	59,180,360	Atlantic - Scotland
H177	*Ssa09*	ctg7180001807792_11224_SAC	59,214,280	Atlantic - Scotland
H178	*Ssa09*	ctg7180001807792_6698_SAG	59,218,806	Atlantic - Scotland
H179	*Ssa09*	ctg7180001807792_3473_SCT	59,222,031	Atlantic - Scotland
H180	*Ssa09*	ctg7180001478185_911_SGT	59,232,485	Atlantic - Scotland
H181	*Ssa09*	ctg7180001343147_3757_SAG	59,235,024	Atlantic - Scotland
H182	*Ssa09*	ctg7180001503789_930_SCT	59,239,714	Atlantic - Scotland
H183	*Ssa09*	ctg7180001503789_1591_SCT	59,240,375	Atlantic - Scotland
H184	*Ssa09*	ctg7180001910931_2027_SCT	59,260,699	Atlantic - Scotland
H185	*Ssa09*	ctg7180001910931_936_SGT	59,261,790	Atlantic - Scotland
H186	*Ssa09*	ctg7180001857343_125_SAG	59,267,390	Atlantic - Scotland
H187	*Ssa09*	ctg7180001857343_4447_SCT	59,271,712	Atlantic - Scotland
H188	*Ssa09*	ctg7180001857343_14669_SCT	59,281,934	Atlantic - Scotland
H189	*Ssa09*	ctg7180001880651_15601_SAC	59,293,193	Atlantic& Cantabric - Scotland
H190	*Ssa09*	ctg7180001880651_13939_SCT	59,294,855	Atlantic - Scotland
H191	*Ssa09*	ctg7180001880651_2788_SGT	59,306,006	Atlantic& Cantabric - Scotland
H192	*Ssa09*	ctg7180001880650_17087_SCT	59,310,176	Atlantic - Scotland
H193	*Ssa09*	ctg7180001880650_16601_SCT	59,310,663	Atlantic - Scotland
H194	*Ssa09*	ctg7180001880650_6322_SAG	59,320,942	Atlantic - Scotland
H195	*Ssa09*	ctg7180001880650_6282_SAG	59,320,982	Atlantic - Scotland
H196	*Ssa09*	ctg7180001880650_5409_SGT	59,321,867	Atlantic - Scotland
H197	*Ssa09*	ctg7180001798132_4101_SAG	59,353,131	Atlantic - Scotland
H198	*Ssa09*	ctg7180001590691_546_SAC	59,354,976	Atlantic - Scotland
H199	*Ssa09*	ctg7180001590691_346_SAC	59,355,176	Atlantic - Scotland
H200	*Ssa09*	ctg7180001430493_2499_SCT	59,355,855	Atlantic - Scotland
H201	*Ssa09*	ctg7180001329698_2004_SAC	59,398,479	Atlantic - Scotland
H202	*Ssa09*	ctg7180001936708_9146_SCT	59,426,403	Atlantic - Scotland
H203	*Ssa09*	ctg7180001936708_18072_SAG	59,435,329	Atlantic - Scotland
H204	*Ssa09*	ctg7180001936708_18687_SAG	59,435,944	Atlantic - Scotland
H205	*Ssa09*	ctg7180001936708_23825_SGT	59,441,082	Atlantic - Scotland
H206	*Ssa09*	ctg7180001794077_1426_SCT	59,445,417	Atlantic - Scotland
H207	*Ssa09*	ctg7180001794077_7541_SAG	59,451,532	Atlantic - Scotland
H208	*Ssa09*	ctg7180001900960_3117_SAC	59,455,456	Atlantic - Scotland
H209	*Ssa09*	ctg7180001590814_342_SAC	59,628,576	Atlantic - Scotland
H210	*Ssa09*	ctg7180001923719_5720_SGT	59,635,030	Atlantic - Scotland
H211	*Ssa09*	ctg7180001923719_8242_SGT	59,637,587	Atlantic - Scotland
H212	*Ssa09*	ctg7180001923719_8366_SAG	59,637,677	Atlantic - Scotland
H213	*Ssa09*	ctg7180001821349_5291_SAG	59,664,868	Atlantic - Scotland

H214	*Ssa09*	ctg7180001862708_2278_SAG	59,851,684	Atlantic - Scotland
H215	*Ssa09*	ctg7180001818102_11644_SAC	59,877,367	Atlantic - Scotland
H216	*Ssa09*	ctg7180001818102_12694_SGT	59,878,417	Atlantic - Scotland
H217	*Ssa09*	ctg7180001818102_14823_SCT	59,880,546	Atlantic - Scotland
H218	*Ssa09*	ctg7180001848824_3128_SAG	59,908,141	Atlantic - Scotland
H219	*Ssa09*	ctg7180001848824_6025_SCT	59,911,038	Atlantic - Scotland
H220	*Ssa09*	ctg7180001848825_513_SAG	59,911,915	Atlantic - Scotland
H221	*Ssa09*	ctg7180001848825_4505_SCT	59,915,907	Atlantic - Scotland
H222	*Ssa09*	ctg7180001874248_14870_SCT	60,004,787	Atlantic - Scotland
H223	*Ssa09*	ctg7180001841311_6068_SAC	60,030,507	Atlantic - Scotland
H224	*Ssa09*	ctg7180001744603_4752_SAC	112,700,513	Cantabric - Scotland
H225	*Ssa09*	ctg7180001898651_2354_SAG	117,342,909	Atlantic - Scotland
H226	*Ssa10*	ctg7180001910335_11899_SCT	7,693,152	Cantabric - Scotland
H227	*Ssa10*	ctg7180001881285_2000_SGT	8,690,402	Cantabric - Scotland
H228	*Ssa10*	ctg7180001873085_5595_SGT	10,589,290	Cantabric - Scotland
H229	*Ssa10*	ctg7180001873085_5852_SCT	10,589,547	Cantabric - Scotland
H230	*Ssa10*	ctg7180001826825_3446_SGT	10,635,181	Cantabric - Scotland
H231	*Ssa10*	ctg7180001433539_2375_SAG	10,673,902	Cantabric - Scotland
H232	*Ssa10*	ctg7180001433539_7568_SAG	10,679,095	Cantabric - Scotland
H233	*Ssa10*	ctg7180001436140_1098_SAG	10,748,109	Cantabric - Scotland
H234	*Ssa10*	ctg7180001835524_8080_SGT	12,600,167	Cantabric - Scotland
H235	*Ssa10*	ctg7180001908181_8404_SAG	57,340,712	Atlantic - Scotland
H236	*Ssa10*	ctg7180001908181_8142_SCT	57,340,974	Atlantic - Scotland
H237	*Ssa10*	ctg7180001549788_3692_SCT	97,674,456	Cantabric - Scotland
H238	*Ssa10*	ctg7180001868951_5834_SCT	97,779,977	Cantabric - Scotland
H239	*Ssa10*	ctg7180001739572_1279_SCT	97,788,507	Cantabric - Scotland
H240	*Ssa10*	ctg7180001615130_768_SAG	97,802,281	Cantabric - Scotland
H241	*Ssa11*	ctg7180001505492_3927_SAG	874,306	Cantabric - Scotland
H242	*Ssa11*	ctg7180001793021_7140_SAG	910,538	Cantabric - Scotland
H243	*Ssa11*	ctg7180001907554_6589_SCT	995,223	Cantabric - Scotland
H244	*Ssa11*	ctg7180001907554_4514_SGT	997,298	Cantabric - Scotland
H245	*Ssa11*	ctg7180001907554_4482_SGT	997,330	Cantabric - Scotland
H246	*Ssa11*	ctg7180001907554_1153_SGT	1,000,659	Cantabric - Scotland
H247	*Ssa11*	ctg7180001904938_3860_SAC	1,005,642	Atlantic& Cantabric - Scotland
H248	*Ssa11*	ctg7180001815957_4948_SGT	1,017,098	Cantabric - Scotland
H249	*Ssa11*	ctg7180001532142_1365_SAC	1,041,139	Atlantic& Cantabric - Scotland
H250	*Ssa11*	ctg7180001532142_1560_SCT	1,041,334	Atlantic& Cantabric - Scotland
H251	*Ssa11*	ctg7180001383734_1324_SCT	1,043,950	Atlantic& Cantabric - Scotland
H252	*Ssa11*	ctg7180001882931_4294_SAC	1,049,881	Atlantic& Cantabric - Scotland
H253	*Ssa11*	ctg7180001882931_8058_SAC	1,053,645	Atlantic& Cantabric - Scotland
H254	*Ssa11*	ctg7180001882932_5229_SAG	1,060,087	Atlantic - Scotland
H255	*Ssa11*	ctg7180001882932_5398_SAG	1,060,256	Atlantic - Scotland
H256	*Ssa11*	ctg7180001824050_3906_SCT	1,090,743	Atlantic& Cantabric - Scotland
H257	*Ssa11*	ctg7180001824050_3693_SGT	1,090,956	Atlantic - Scotland
H258	*Ssa11*	ctg7180001210942_818	1,095,585	Atlantic - Scotland
H259	*Ssa11*	ctg7180001210942_1639_SGT	1,096,371	Atlantic - Scotland
H260	*Ssa11*	ctg7180001921147_3977_SCT	1,117,469	Atlantic - Scotland
H261	*Ssa11*	ctg7180001921146_8382_SGT	1,127,695	Atlantic - Scotland
H262	*Ssa11*	ctg7180001613816_649_SCT	1,143,485	Atlantic - Scotland

H263	*Ssa11*	ctg7180001881465_7357_SAC	1,146,090	Atlantic - Scotland
H264	*Ssa11*	ctg7180001881465_6786_SAC	1,146,661	Atlantic - Scotland
H265	*Ssa11*	ctg7180001270964_8296_SAC	1,157,071	Atlantic& Cantabric - Scotland
H266	*Ssa11*	ctg7180001270964_477_SAG	1,164,890	Atlantic& Cantabric - Scotland
H267	*Ssa11*	ctg7180001270964_386_SCT	1,164,981	Atlantic - Scotland
H268	*Ssa11*	ctg7180001270964_362_SCT	1,165,005	Atlantic - Scotland
H269	*Ssa11*	ctg7180001915670_1543_SAC	1,167,122	Atlantic - Scotland
H270	*Ssa11*	ctg7180001893240_4064_SAG	1,210,370	Atlantic& Cantabric - Scotland
H271	*Ssa11*	ctg7180001893240_7158_SGT	1,213,499	Atlantic - Scotland
H272	*Ssa11*	ctg7180001893240_7351_SAG	1,213,657	Atlantic - Scotland
H273	*Ssa11*	ctg7180001858710_12327_SAG	1,234,760	Atlantic - Scotland
H274	*Ssa11*	ctg7180001858710_21824_SGT	1,244,257	Atlantic - Scotland
H275	*Ssa11*	ctg7180001309811_341_SAC	1,245,184	Atlantic& Cantabric - Scotland
H276	*Ssa11*	ctg7180001309811_750_SGT	1,245,593	Atlantic - Scotland
H277	*Ssa11*	ctg7180001914231_19513_SGT	1,257,829	Atlantic - Scotland
H278	*Ssa11*	ctg7180001914231_13917_SAG	1,263,425	Atlantic - Scotland
H279	*Ssa11*	ctg7180001914231_13301_SAG	1,264,041	Atlantic - Scotland
H280	*Ssa11*	ctg7180001914231_19694_SCT	1,273,688	Atlantic - Scotland
H281	*Ssa11*	ctg7180001853978_15040_SAG	1,297,150	Atlantic& Cantabric - Scotland
H282	*Ssa11*	ctg7180001865052_3369_SCT	1,361,087	Atlantic - Scotland
H283	*Ssa11*	ctg7180001923148_10333_SGT	1,385,706	Atlantic - Scotland
H284	*Ssa11*	ctg7180001923148_10503_SCT	1,385,876	Atlantic - Scotland
H285	*Ssa11*	ctg7180001923148_10929_SAG	1,386,302	Atlantic - Scotland
H286	*Ssa11*	ctg7180001923148_11138_SCT	1,386,511	Atlantic - Scotland
H287	*Ssa11*	ctg7180001923148_14460_SAG	1,389,833	Atlantic - Scotland
H288	*Ssa11*	ctg7180001923148_21116_SCT	1,396,489	Atlantic - Scotland
H289	*Ssa11*	ctg7180001923148_21898_SAG	1,397,271	Atlantic - Scotland
H290	*Ssa11*	ctg7180001923148_22117_SAG	1,397,490	Atlantic - Scotland
H291	*Ssa11*	ctg7180001923148_22465_SGT	1,397,838	Atlantic - Scotland
H292	*Ssa11*	ctg7180001923148_23339_SCT	1,398,718	Atlantic - Scotland
H293	*Ssa11*	ctg7180001912483_1150_SAG	1,405,904	Atlantic - Scotland
H294	*Ssa11*	ctg7180001912483_12052_SCT	1,416,806	Atlantic - Scotland
H295	*Ssa11*	ctg7180001899290_23531_SCT	1,464,450	Atlantic - Scotland
H296	*Ssa11*	ctg7180001899290_18435_SGT	1,469,546	Atlantic - Scotland
H297	*Ssa11*	ctg7180001899290_15307_SAG	1,473,123	Atlantic& Cantabric - Scotland
H298	*Ssa11*	ctg7180001899290_14531_SAC	1,473,450	Atlantic& Cantabric - Scotland
H299	*Ssa11*	ctg7180001899290_10283_SAC	1,477,698	Atlantic& Cantabric - Scotland
H300	*Ssa11*	ctg7180001899289_1861_SCT	1,493,800	Atlantic& Cantabric - Scotland
H301	*Ssa11*	ctg7180001831873_5394_SAC	1,528,954	Atlantic& Cantabric - Scotland
H302	*Ssa11*	ctg7180001883718_7638_SCT	1,572,829	Atlantic - Scotland
H303	*Ssa11*	ctg7180001901617_8656_SCT	1,589,543	Atlantic - Scotland
H304	*Ssa11*	ctg7180001886989_1294_SGT	1,747,096	Atlantic - Scotland
H305	*Ssa11*	ctg7180001831322_6749_SAC	1,799,504	Atlantic - Scotland
H306	*Ssa11*	ctg7180001848210_4086_SCT	1,802,176	Atlantic - Scotland
H307	*Ssa11*	ctg7180001848210_3857_SCT	1,802,405	Atlantic - Scotland
H308	*Ssa11*	ctg7180001848210_634_SAC	1,805,628	Atlantic - Scotland
H309	*Ssa11*	ctg7180001848210_615_SCT	1,805,647	Atlantic - Scotland
H310	*Ssa11*	ctg7180001859185_2746_SAC	1,822,335	Atlantic& Cantabric - Scotland
H311	*Ssa11*	ctg7180001859185_3461_SAG	1,823,050	Atlantic - Scotland

H312	*Ssa11*	ctg7180001859185_3875_SAC	1,823,464	Atlantic - Scotland
H313	*Ssa11*	ctg7180001734006_1590_SCT	1,837,721	Atlantic - Scotland
H314	*Ssa11*	ctg7180001843450_18275_SAG	1,853,254	Atlantic - Scotland
H315	*Ssa11*	ctg7180001843450_14099_SAG	1,857,430	Atlantic - Scotland
H316	*Ssa11*	ctg7180001843450_4015_SAG	1,867,515	Atlantic - Scotland
H317	*Ssa11*	ctg7180001843450_3924_SCT	1,867,606	Atlantic - Scotland
H318	*Ssa11*	ctg7180001826880_3896_SAG	1,906,544	Atlantic - Scotland
H319	*Ssa11*	ctg7180001267856_502_SCT	1,985,119	Atlantic - Scotland
H320	*Ssa11*	ctg7180001344387_609_SAC	1,986,163	Atlantic - Scotland
H321	*Ssa11*	ctg7180001344387_1122_SCT	1,986,676	Atlantic - Scotland
H322	*Ssa11*	ctg7180001719742_1128_SCT	1,989,872	Atlantic - Scotland
H323	*Ssa11*	ctg7180001878029_2992_SAC	2,008,424	Atlantic - Scotland
H324	*Ssa11*	ctg7180001869773_5106_SGT	2,012,544	Atlantic - Scotland
H325	*Ssa11*	ctg7180001275940_3090_SCT	2,095,841	Atlantic - Scotland
H326	*Ssa11*	ctg7180001804348_10282_SAC	4,029,683	Atlantic - Scotland
H327	*Ssa11*	ctg7180001848151_1867_SAG	5,016,230	Atlantic - Scotland
H328	*Ssa11*	ctg7180001835390_13690_SAC	5,609,706	Atlantic - Scotland
H329	*Ssa11*	ctg7180001910695_8496_SAG	5,649,247	Atlantic - Scotland
H330	*Ssa11*	ctg7180001910695_6610_SGT	5,651,133	Atlantic - Scotland
H331	*Ssa11*	ctg7180001904475_6737_SCT	77,031,378	Atlantic - Scotland
H332	*Ssa11*	ctg7180001852881_7065_SGT	77,057,570	Atlantic - Scotland
H333	*Ssa11*	ctg7180001541637_66_SCT	77,074,011	Atlantic - Scotland
H334	*Ssa11*	ctg7180001753246_79_SCT	77,089,857	Atlantic - Scotland
H335	*Ssa11*	ctg7180001878972_374_SAG	77,112,559	Atlantic - Scotland
H336	*Ssa12*	ctg7180001299154_3833_SAG	36,223,137	Atlantic - Scotland
H337	*Ssa12*	ctg7180001802246_5796_SGT	36,528,557	Atlantic - Scotland
H338	*Ssa12*	ctg7180001803899_3428_SCG	36,573,142	Atlantic - Scotland
H339	*Ssa13*	ctg7180001899453_5271_SCT	65,545,947	Atlantic - Cantabric
H340	*Ssa13*	ctg7180001805890_9129_SAG	65,709,860	Atlantic - Cantabric
H341	*Ssa14*	ctg7180001439715_9021_SAC	61,526,521	Atlantic - Scotland
H342	*Ssa14*	ctg7180001439715_9362_SAG	61,526,862	Atlantic - Scotland
H343	*Ssa14*	ctg7180001686171_3826_SAC	61,533,936	Atlantic - Scotland
H344	*Ssa14*	ctg7180001766677_331_SCT	61,537,820	Atlantic - Scotland
H345	*Ssa14*	ctg7180001766677_665_SCT	61,538,117	Atlantic - Scotland
H346	*Ssa14*	ctg7180001898178_1544_SGT	61,623,028	Atlantic - Scotland
H347	*Ssa14*	ctg7180001899974_55334_SAG	61,629,355	Atlantic - Scotland
H348	*Ssa14*	ctg7180001899974_54482_SCT	61,630,207	Atlantic - Scotland
H349	*Ssa14*	ctg7180001899974_54438_SAG	61,630,251	Atlantic - Scotland
H350	*Ssa14*	ctg7180001899974_53790_SAG	61,630,899	Atlantic - Scotland
H351	*Ssa14*	ctg7180001899974_47280_SAG	61,637,411	Atlantic - Scotland
H352	*Ssa14*	ctg7180001899974_47053_SAG	61,637,638	Atlantic - Scotland
H353	*Ssa14*	ctg7180001899974_46545_SAG	61,638,146	Atlantic - Scotland
H354	*Ssa14*	ctg7180001899974_46497_SAG	61,638,194	Atlantic - Scotland
H355	*Ssa14*	ctg7180001899974_46494_SAC	61,638,197	Atlantic - Scotland
H356	*Ssa14*	ctg7180001899974_45976_SAC	61,638,715	Atlantic - Scotland
H357	*Ssa14*	ctg7180001899974_45406_SGT	61,639,285	Atlantic - Scotland
H358	*Ssa14*	ctg7180001899974_45184_SAG	61,639,507	Atlantic - Scotland
H359	*Ssa14*	ctg7180001899974_45181_SCT	61,639,510	Atlantic - Scotland
H360	*Ssa14*	ctg7180001899974_45064_SCT	61,639,627	Atlantic - Scotland

H361	*Ssa14*	ctg7180001899974_44980_SCT	61,639,711	Atlantic - Scotland
H362	*Ssa14*	ctg7180001899974_43833_SCT	61,640,858	Atlantic - Scotland
H363	*Ssa14*	ctg7180001899974_43418_SGT	61,641,273	Atlantic - Scotland
H364	*Ssa14*	ctg7180001899974_39915_SAG	61,644,776	Atlantic - Scotland
H365	*Ssa14*	ctg7180001899974_39585_SCT	61,645,106	Atlantic - Scotland
H366	*Ssa14*	ctg7180001899974_35399_SCT	61,649,292	Atlantic - Scotland
H367	*Ssa14*	ctg7180001912177_4596_SAC	68,296,505	Atlantic - Scotland
H368	*Ssa14*	ctg7180001912177_4249_SCT	68,296,852	Atlantic - Scotland
H369	*Ssa14*	ctg7180001882025_6604_SAG	76,709,770	Cantabric - Scotland
H370	*Ssa15*	ctg7180001821128_12002_SAC	27,751,213	Cantabric - Scotland
H371	*Ssa15*	ctg7180001821128_11976_SAC	27,751,239	Cantabric - Scotland
H372	*Ssa15*	ctg7180001893122_4750_SCT	66,167,141	Atlantic - Scotland
H373	*Ssa15*	ctg7180001347103_6538_SAG	66,174,746	Atlantic - Scotland
H374	*Ssa15*	ctg7180001225716_4839_SAG	71,041,793	Cantabric - Scotland
H375	*Ssa15*	ctg7180001789781_1476_SGT	78,424,592	Atlantic - Scotland
H376	*Ssa15*	ctg7180001789781_2046_SCT	78,425,162	Atlantic - Scotland
H377	*Ssa15*	ctg7180001463780_1438_SCT	95,822,541	Cantabric - Scotland
H378	*Ssa16*	ctg7180001690596_1831_SCT	29,048,609	Atlantic - Scotland
H379	*Ssa16*	ctg7180001187717_2499_SAG	29,104,938	Atlantic& Cantabric - Scotland
H380	*Ssa16*	ctg7180001187717_1844_SAC	29,105,593	Atlantic& Cantabric - Scotland
H381	*Ssa16*	ctg7180001684182_2268_SAC	29,247,269	Atlantic - Scotland
H382	*Ssa16*	ctg7180001629042_26172_SCT	29,259,954	Atlantic - Scotland
H383	*Ssa16*	ctg7180001232716_4779_SGT	29,291,371	Atlantic - Scotland
H384	*Ssa16*	ctg7180001563794_1084	29,314,299	Atlantic - Scotland
H385	*Ssa16*	ctg7180001824301_1009_SGT	29,382,992	Atlantic - Scotland
H386	*Ssa16*	ctg7180001911673_8376_SCT	29,891,003	Atlantic - Scotland
H387	*Ssa16*	ctg7180001830338_1987_SAG	29,898,378	Atlantic - Scotland
H388	*Ssa16*	ctg7180001830338_2646_SAC	29,899,037	Atlantic - Scotland
H389	*Ssa16*	ctg7180001818928_1075_SGT	29,929,051	Atlantic - Scotland
H390	*Ssa16*	ctg7180001877117_5794_SAG	29,945,805	Atlantic - Scotland
H391	*Ssa16*	ctg7180001827929_736_SAC	29,949,157	Atlantic - Scotland
H392	*Ssa16*	GCR_cBin16227_Ctg1_85	29,966,143	Atlantic - Scotland
H393	*Ssa16*	ctg7180001795854_411_SCT	29,966,454	Atlantic - Scotland
H394	*Ssa16*	ctg7180001795854_730_SAG	29,966,773	Atlantic - Scotland
H395	*Ssa16*	ctg7180001795854_1995_SGT	29,968,038	Atlantic - Scotland
H396	*Ssa16*	ctg7180001795854_2466_SAG	29,968,509	Atlantic - Scotland
H397	*Ssa16*	ctg7180001795854_5651_SCT	29,971,693	Atlantic - Scotland
H398	*Ssa16*	ctg7180001279355_3440_SGT	29,983,686	Atlantic - Scotland
H399	*Ssa16*	ctg7180001279355_1655_SAG	29,985,471	Atlantic - Scotland
H400	*Ssa16*	ctg7180001279355_59_SCT	29,987,067	Atlantic - Scotland
H401	*Ssa16*	ctg7180001589388_1492_SGT	30,018,748	Atlantic - Scotland
H402	*Ssa16*	ctg7180001852796_12159_SAC	30,069,262	Atlantic - Scotland
H403	*Ssa16*	ctg7180001562309_1991_SGT	30,197,783	Atlantic - Scotland
H404	*Ssa16*	ctg7180001574197_675_SAG	30,204,693	Atlantic - Scotland
H405	*Ssa16*	ctg7180001581361_1577_SCT	30,209,377	Atlantic - Scotland
H406	*Ssa17*	ctg7180001871360_2456_SAC	4,499,082	Atlantic - Scotland
H407	*Ssa17*	ctg7180001871360_5835_SAG	4,502,461	Atlantic - Scotland
H408	*Ssa17*	ctg7180001894687_4835_SGT	5,507,371	Atlantic - Cantabric
H409	*Ssa17*	ctg7180001895274_64_SCT	5,557,108	Atlantic - Cantabric

H410	*Ssa17*	ctg7180001926196_6568_SAG	5,575,350	Atlantic - Cantabric
H411	*Ssa17*	ctg7180001825609_496_SGT	5,725,753	Atlantic - Cantabric
H412	*Ssa17*	ctg7180001732592_2350_SCT	5,726,785	Atlantic - Cantabric
H413	*Ssa17*	ctg7180001902025_3556_SAG	6,850,553	Atlantic - Scotland
H414	*Ssa17*	ctg7180001902025_1418_SAC	6,852,691	Atlantic - Scotland
H415	*Ssa17*	ctg7180001922955_5846_SAG	6,860,933	Atlantic - Scotland
H416	*Ssa17*	ctg7180001922955_7029_SAC	6,862,116	Atlantic - Scotland
H417	*Ssa17*	ctg7180001922955_9218_SAG	6,864,305	Atlantic - Scotland
H418	*Ssa17*	ctg7180001901180_4957_SAG	12,948,551	Atlantic - Scotland
H419	*Ssa17*	ctg7180001901180_596_SCT	12,952,912	Atlantic - Scotland
H420	*Ssa17*	ctg7180001735894_1240_SGT	12,959,113	Atlantic - Scotland
H421	*Ssa17*	ctg7180001480207_2275_SAG	12,960,485	Atlantic - Scotland
H422	*Ssa17*	ctg7180001480207_1858_SCT	12,960,902	Atlantic - Scotland
H423	*Ssa17*	ctg7180001480207_1440_SCT	12,961,320	Atlantic - Scotland
H424	*Ssa17*	ctg7180001480207_671_SCT	12,962,089	Atlantic - Scotland
H425	*Ssa17*	ctg7180001480207_234_SGT	12,962,526	Atlantic - Scotland
H426	*Ssa17*	ctg7180001780683_4176_SAG	12,969,811	Atlantic - Scotland
H427	*Ssa17*	ctg7180001822119_6429_SCT	13,018,433	Atlantic - Scotland
H428	*Ssa18*	ctg7180001432617_3815_SAG	4,445,418	Atlantic - Scotland
H429	*Ssa18*	ctg7180001703813_1551_SAC	59,027,193	Atlantic - Scotland
H430	*Ssa18*	ctg7180001703813_1713_SCT	59,027,355	Atlantic - Scotland
H431	*Ssa18*	ctg7180001703813_1948_SGT	59,027,590	Atlantic& Cantabric - Scotland
H432	*Ssa19*	ctg7180001799854_3907_SCT	4,246,376	Atlantic - Scotland
H433	*Ssa19*	ctg7180001908453_13063_SCT	8,176,370	Cantabric - Scotland
H434	*Ssa19*	ctg7180001196981_2351_SCT	8,200,317	Cantabric - Scotland
H435	*Ssa19*	ctg7180001842604_3938_SGT	8,210,801	Cantabric - Scotland
H436	*Ssa19*	ctg7180001805830_328_SAG	10,956,263	Atlantic - Scotland
H437	*Ssa20*	ctg7180001822949_1132_SCT	9,063,295	Atlantic - Scotland
H438	*Ssa20*	ctg7180001822949_896_SAG	9,063,531	Atlantic - Scotland
H439	*Ssa20*	ctg7180001458779_131_SGT	12,877,492	Atlantic - Cantabric
H440	*Ssa20*	ctg7180001859363_19054_SCT	13,742,992	Atlantic - Scotland
H441	*Ssa20*	ctg7180001875589_5087_SGT	51,348,431	Cantabric - Scotland
H442	*Ssa20*	ctg7180001289793_1211_SAC	51,400,831	Cantabric - Scotland
H443	*Ssa20*	ctg7180001932294_6712_SCT	51,409,488	Cantabric - Scotland
H444	*Ssa21*	ctg7180001910313_8618_SAG	15,460,165	Atlantic - Scotland
H445	*Ssa21*	ctg7180001881048_749_SAG	15,781,775	Atlantic - Scotland
H446	*Ssa21*	ctg7180001870953_1829_SCT	16,070,580	Atlantic - Scotland
H447	*Ssa21*	ctg7180001870953_2034_SAG	16,070,785	Atlantic - Scotland
H448	*Ssa21*	ctg7180001900148_13156_SGT	16,080,694	Atlantic - Scotland
H449	*Ssa21*	ctg7180001900148_9051_SAG	16,084,799	Atlantic - Scotland
H450	*Ssa21*	ctg7180001794673_4695_SAC	41,360,019	Atlantic - Scotland
H451	*Ssa21*	ctg7180001843512_6137_SAG	49,908,772	Atlantic - Scotland
H452	*Ssa21*	ctg7180001895237_1805_SAG	50,142,608	Cantabric - Scotland
H453	*Ssa22*	ctg7180001872597_6133_SAG	21,063,319	Atlantic - Scotland
H454	*Ssa22*	ctg7180001872597_2430_SCT	21,067,022	Atlantic - Scotland
H455	*Ssa22*	ctg7180001886294_2306_SAG	21,095,730	Atlantic - Scotland
H456	*Ssa22*	ctg7180001886294_3123_SAC	21,096,547	Atlantic - Scotland
H457	*Ssa22*	ctg7180001886294_4976_SAG	21,098,400	Atlantic - Scotland
H458	*Ssa22*	ctg7180001911652_4500_SAG	21,105,147	Atlantic - Scotland

H459	*Ssa22*	ctg7180001911652_6459_SAG	21,107,106	Atlantic - Scotland
H460	*Ssa22*	ctg7180001922606_10111_SGT	21,136,117	Atlantic - Scotland
H461	*Ssa22*	ctg7180001922606_25030	21,146,040	Atlantic - Scotland
H462	*Ssa22*	ctg7180001510474_892_SGT	21,208,122	Atlantic - Scotland
H463	*Ssa22*	ctg7180001791406_3889_SAC	21,284,102	Atlantic - Scotland
H464	*Ssa22*	ctg7180001791406_4749_SAG	21,284,962	Atlantic - Scotland
H465	*Ssa22*	ctg7180001791406_6499_SCT	21,286,712	Atlantic - Scotland
H466	*Ssa22*	ctg7180001791406_8999_SAG	21,289,212	Atlantic - Scotland
H467	*Ssa22*	ctg7180001861102_1098_SAG	21,294,818	Atlantic - Scotland
H468	*Ssa22*	ctg7180001861104_1736_SAG	21,307,877	Atlantic - Scotland
H469	*Ssa22*	ctg7180001861104_5482_SGT	21,311,588	Atlantic& Cantabric - Scotland
H470	*Ssa22*	ctg7180001861104_6760_SCT	21,312,866	Atlantic - Scotland
H471	*Ssa22*	ctg7180001790123_1093_SCT	21,391,174	Atlantic - Scotland
H472	*Ssa22*	ctg7180001817574_6975_SGT	28,796,204	Cantabric - Scotland
H473	*Ssa22*	ctg7180001569764_256_SAC	28,847,745	Cantabric - Scotland
H474	*Ssa24*	ctg7180001866170_4096_SCT	23,941,228	Atlantic - Scotland
H475	*Ssa24*	ctg7180001915028_7514_SAG	24,944,591	Atlantic - Scotland
H476	*Ssa24*	ctg7180001856738_104_SGT	25,097,646	Atlantic - Scotland
H477	*Ssa24*	ctg7180001821474_3967_SAG	25,118,090	Atlantic - Scotland
H478	*Ssa24*	ctg7180001516867_2850_SCT	25,173,557	Cantabric - Scotland
H479	*Ssa24*	ctg7180001908592_4387_SCT	25,178,986	Atlantic& Cantabric - Scotland
H480	*Ssa24*	ctg7180001908592_10854_SCT	25,185,453	Cantabric - Scotland
H481	*Ssa24*	ctg7180001908592_11488_SGT	25,186,087	Atlantic& Cantabric - Scotland
H482	*Ssa24*	ctg7180001908592_15027_SAC	25,189,626	Atlantic& Cantabric - Scotland
H483	*Ssa24*	ctg7180001908592_22106_SAG	25,196,705	Atlantic& Cantabric - Scotland
H484	*Ssa24*	ctg7180001908592_22338_SAG	25,196,937	Cantabric - Scotland
H485	*Ssa24*	ctg7180001931927_12945_SAC	25,224,283	Atlantic& Cantabric - Scotland
H486	*Ssa24*	ctg7180001878964_1947_SCT	25,239,312	Cantabric - Scotland
H487	*Ssa24*	ctg7180001878964_2058_SGT	25,239,423	Cantabric - Scotland
H488	*Ssa24*	ctg7180001812117_1745_SGT	25,249,910	Atlantic& Cantabric - Scotland
H489	*Ssa24*	ctg7180001812117_2070_SAC	25,250,235	Atlantic& Cantabric - Scotland
H490	*Ssa24*	ctg7180001812117_2701_SCT	25,250,866	Atlantic& Cantabric - Scotland
H491	*Ssa24*	ctg7180001425328_580_SCT	25,256,160	Atlantic& Cantabric - Scotland
H492	*Ssa24*	ctg7180001807519_15025_SCT	25,272,938	Atlantic - Scotland
H493	*Ssa24*	ctg7180001807519_8272_SAG	25,280,830	Atlantic - Scotland
H494	*Ssa24*	ctg7180001807519_451_SAG	25,287,512	Atlantic - Scotland
H495	*Ssa24*	ctg7180001741632_299_SGT	25,291,395	Atlantic - Scotland
H496	*Ssa24*	ctg7180001919198_16843_SGT	25,317,008	Atlantic - Scotland
H497	*Ssa24*	ctg7180001919198_7460_SAC	25,326,389	Atlantic - Scotland
H498	*Ssa24*	ctg7180001919198_4458_SAG	25,329,391	Atlantic - Scotland
H499	*Ssa24*	ctg7180001919198_306_SAG	25,333,542	Atlantic - Scotland
H500	*Ssa24*	ctg7180001899698_7089_SCT	25,502,040	Atlantic - Scotland
H501	*Ssa24*	ctg7180001919737_18870_SGT	25,514,293	Atlantic - Scotland
H502	*Ssa24*	ctg7180001919737_3620_SGT	25,529,543	Atlantic - Scotland
H503	*Ssa24*	ctg7180001817456_1115_SCT	25,537,226	Atlantic& Cantabric - Scotland
H504	*Ssa24*	ctg7180001817456_2124_SCT	25,538,633	Atlantic - Scotland
H505	*Ssa24*	ctg7180001839382_173_SGT	25,539,413	Atlantic - Scotland
H506	*Ssa24*	ctg7180001839382_2024_SAG	25,541,299	Atlantic - Scotland
H507	*Ssa24*	ctg7180001810142_632_SCT	25,554,902	Atlantic - Scotland

H508	*Ssa24*	ctg7180001810142_807_SGT	25,555,077	Atlantic - Scotland
H509	*Ssa24*	ctg7180001810142_5402_SAG	25,559,672	Atlantic - Scotland
H510	*Ssa24*	ctg7180001810142_10425_SGT	25,564,695	Atlantic - Scotland
H511	*Ssa24*	ctg7180001850076_10066_SAG	25,635,255	Atlantic - Scotland
H512	*Ssa24*	ctg7180001850076_250_SAC	25,645,595	Atlantic - Scotland
H513	*Ssa24*	ctg7180001778985_687_SAG	25,669,630	Atlantic - Scotland
H514	*Ssa24*	ctg7180001794579_12715_SAC	25,674,456	Atlantic - Scotland
H515	*Ssa24*	ctg7180001627869_731_SCT	25,840,216	Atlantic - Scotland
H516	*Ssa24*	ctg7180001469599_940_SCT	25,864,496	Atlantic - Scotland
H517	*Ssa24*	ctg7180001469599_5312_SCT	25,868,868	Atlantic - Scotland
H518	*Ssa24*	ctg7180001876298_2225_SAG	25,871,806	Atlantic - Scotland
H519	*Ssa24*	ctg7180001935000_14361_SAC	25,919,189	Atlantic - Scotland
H520	*Ssa24*	ctg7180001935000_14228_SCT	25,919,322	Atlantic - Scotland
H521	*Ssa24*	ctg7180001180420_1426_SCT	25,936,607	Atlantic - Scotland
H522	*Ssa24*	ctg7180001180420_802_SCT	25,937,231	Atlantic - Scotland
H523	*Ssa24*	ctg7180001902878_13479_SAG	25,943,149	Atlantic - Scotland
H524	*Ssa24*	ctg7180001902878_11547_SCT	25,945,081	Atlantic - Scotland
H525	*Ssa24*	ctg7180001902878_9833_SCT	25,946,795	Atlantic - Scotland
H526	*Ssa24*	ctg7180001902878_3616_SAG	25,953,012	Atlantic - Scotland
H527	*Ssa24*	ctg7180001857040_2423_SAG	25,964,712	Atlantic - Scotland
H528	*Ssa24*	ctg7180001857040_15721_SAG	25,978,010	Atlantic - Scotland
H529	*Ssa24*	ctg7180001857040_18228_SAG	25,980,517	Atlantic - Scotland
H530	*Ssa24*	ctg7180001546415_11728_SAG	25,999,329	Atlantic - Scotland
H531	*Ssa24*	ctg7180001546415_9418_SAG	26,001,639	Atlantic& Cantabric - Scotland
H532	*Ssa24*	ctg7180001546415_2232_SAG	26,008,825	Atlantic - Scotland
H533	*Ssa24*	ctg7180001890582_1324_SAG	26,059,207	Atlantic - Scotland
H534	*Ssa24*	ctg7180001890582_11181_SAG	26,070,018	Atlantic - Scotland
H535	*Ssa24*	ctg7180001918049_3927_SAG	26,103,524	Atlantic - Scotland
H536	*Ssa24*	ctg7180001918049_3340_SCT	26,104,111	Atlantic - Scotland
H537	*Ssa24*	ctg7180001758461_1649_SAC	26,127,186	Atlantic - Scotland
H538	*Ssa24*	ctg7180001357756_2268_SCT	26,138,709	Atlantic - Scotland
H539	*Ssa24*	ctg7180001788059_469_SAC	26,173,065	Atlantic - Scotland
H540	*Ssa24*	ctg7180001301776_6698_SGT	26,188,282	Atlantic - Scotland
H541	*Ssa24*	ctg7180001301776_5842_SAG	26,189,138	Atlantic - Scotland
H542	*Ssa24*	ctg7180001787258_2803_SAG	33,916,510	Atlantic - Scotland
H543	*Ssa24*	ctg7180001787258_1514_SAC	33,917,799	Atlantic - Scotland
H544	*Ssa24*	ctg7180001787258_213_SGT	33,919,100	Atlantic - Scotland
H545	*Ssa24*	ctg7180001787258_179_SCT	33,919,134	Atlantic - Scotland
H546	*Ssa24*	ctg7180001873440_14271_SAG	34,238,364	Atlantic - Scotland
H547	*Ssa24*	ctg7180001873440_11804_SAC	34,240,831	Cantabric - Scotland
H548	*Ssa24*	ctg7180001873440_11406_SCT	34,241,229	Atlantic - Scotland
H549	*Ssa24*	ctg7180001873440_4390_SAC	34,248,245	Atlantic - Scotland
H550	*Ssa24*	ctg7180001442840_2717_SCT	34,254,971	Atlantic& Cantabric - Scotland
H551	*Ssa24*	ctg7180001667216_3777_SGT	34,285,495	Cantabric - Scotland
H552	*Ssa24*	ctg7180001667216_3795_SGT	34,285,513	Cantabric - Scotland
H553	*Ssa24*	ctg7180001917002_11499_SCT	34,307,595	Atlantic - Scotland
H554	*Ssa24*	ctg7180001438770_1771_SAG	34,346,206	Cantabric - Scotland
H555	*Ssa24*	ctg7180001877673_7001_SGT	34,363,076	Atlantic - Scotland
H556	*Ssa24*	ctg7180001831265_4965_SAG	34,392,645	Atlantic - Scotland

H557	*Ssa24*	ctg7180001444597_14097_SGT	34,396,181	Atlantic& Cantabric - Scotland
H558	*Ssa24*	ctg7180001823327_1122_SAG	34,411,670	Atlantic& Cantabric - Scotland
H559	*Ssa24*	ctg7180001823327_3720_SCT	34,414,267	Atlantic& Cantabric - Scotland
H560	*Ssa24*	ctg7180001823327_8293_SAG	34,418,840	Atlantic& Cantabric - Scotland
H561	*Ssa24*	ctg7180001924378_2105_SAC	34,426,792	Atlantic& Cantabric - Scotland
H562	*Ssa24*	ctg7180001924378_2273_SGT	34,426,960	Atlantic& Cantabric - Scotland
H563	*Ssa24*	ctg7180001924378_6311_SCT	34,430,998	Atlantic& Cantabric - Scotland
H564	*Ssa24*	ctg7180001924378_8657_SAC	34,433,344	Atlantic& Cantabric - Scotland
H565	*Ssa24*	ctg7180001924378_10026_SAC	34,434,713	Atlantic& Cantabric - Scotland
H566	*Ssa24*	ctg7180001671303_5671_SCT	34,500,517	Atlantic& Cantabric - Scotland
H567	*Ssa24*	ctg7180001873903_10850_SAG	34,526,840	Atlantic - Scotland
H568	*Ssa24*	ctg7180001873903_7471_SAG	34,530,219	Atlantic - Scotland
H569	*Ssa24*	ctg7180001832887_6847_SCT	34,579,691	Atlantic& Cantabric - Scotland
H570	*Ssa24*	ctg7180001832887_4244_SGT	34,582,294	Atlantic - Scotland
H571	*Ssa24*	ctg7180001832887_3346_SGT	34,583,192	Atlantic - Scotland
H572	*Ssa24*	ctg7180001934797_11171_SAG	34,594,910	Atlantic - Scotland
H573	*Ssa24*	ctg7180001934797_5659_SGT	34,600,422	Atlantic - Scotland
H574	*Ssa24*	ctg7180001934797_3831_SAG	34,602,250	Atlantic - Scotland
H575	*Ssa24*	ctg7180001934797_956_SCT	34,605,125	Atlantic - Scotland
H576	*Ssa24*	ctg7180001739432_1031_SCT	34,607,059	Atlantic - Scotland
H577	*Ssa24*	ctg7180001739432_2109_SCT	34,608,137	Atlantic& Cantabric - Scotland
H578	*Ssa24*	ctg7180001739432_3082_SAG	34,609,110	Atlantic& Cantabric - Scotland
H579	*Ssa24*	ctg7180001826026_27021_SCT	34,622,839	Atlantic - Scotland
H580	*Ssa24*	ctg7180001826026_19583_SAG	34,630,604	Atlantic - Scotland
H581	*Ssa24*	ctg7180001826026_19535_SAG	34,630,652	Atlantic - Scotland
H582	*Ssa24*	ctg7180001826026_18626_SGT	34,631,561	Atlantic& Cantabric - Scotland
H583	*Ssa24*	ctg7180001826026_17233_SAG	34,632,954	Atlantic& Cantabric - Scotland
H584	*Ssa24*	ctg7180001826026_13446_SAG	34,636,741	Atlantic& Cantabric - Scotland
H585	*Ssa24*	ctg7180001826026_17709_SCT	34,639,940	Atlantic& Cantabric - Scotland
H586	*Ssa24*	ctg7180001919078_12605_SCT	34,653,643	Atlantic - Scotland
H587	*Ssa24*	ctg7180001919078_10373_SGT	34,655,875	Cantabric - Scotland
H588	*Ssa24*	ctg7180001919078_9062_SAC	34,657,186	Atlantic - Scotland
H589	*Ssa24*	ctg7180001919078_8281_SCT	34,657,967	Atlantic& Cantabric - Scotland
H590	*Ssa24*	ctg7180001919078_6438_SCT	34,659,810	Atlantic - Scotland
H591	*Ssa24*	ctg7180001919078_4019_SCT	34,662,229	Cantabric - Scotland
H592	*Ssa24*	ctg7180001842969_6949_SCT	34,691,349	Cantabric - Scotland
H593	*Ssa25*	ctg7180001293867_1285_SCT	7,175,102	Atlantic - Cantabric
H594	*Ssa25*	ctg7180001293867_2418_SCT	7,176,323	Atlantic - Cantabric
H595	*Ssa25*	ctg7180001870040_8892_SAC	32,308,767	Atlantic - Scotland
H596	*Ssa25*	ctg7180001267213_2822_SAG	32,316,665	Atlantic - Scotland
H597	*Ssa25*	ctg7180001734517_847_SCT	32,362,494	Atlantic - Scotland
H598	*Ssa25*	ctg7180001928497_2262_SAC	32,983,986	Cantabric - Scotland
H599	*Ssa26*	ctg7180001664107_1632_SAC	6,121,954	Atlantic - Scotland
H600	*Ssa26*	ctg7180001804697_4461_SCT	29,506,080	Atlantic - Cantabric
H601	*Ssa26*	ctg7180001879095_18614_SGT	29,547,999	Atlantic - Cantabric
H602	*Ssa27*	ctg7180001436605_2179_SCT	17,063,988	Atlantic& Cantabric - Scotland
H603	*Ssa27*	ctg7180001436605_2724_SCT	17,064,533	Atlantic& Cantabric - Scotland
H604	*Ssa27*	ctg7180001545651_306_SCT	17,070,515	Atlantic - Scotland
H605	*Ssa27*	ctg7180001545651_364_SGT	17,070,573	Atlantic - Scotland

H606	*Ssa27*	ctg7180001545651_652_SCT	17,070,861	Atlantic - Scotland
H607	*Ssa27*	ctg7180001576823_458_SCT	17,073,799	Atlantic - Scotland
H608	*Ssa27*	ctg7180001869134_308_SAG	17,082,064	Atlantic - Scotland
H609	*Ssa27*	ctg7180001583406_3778_SAG	17,093,128	Atlantic - Scotland
H610	*Ssa27*	ctg7180001583406_2745_SAC	17,094,161	Atlantic - Scotland
H611	*Ssa27*	ctg7180001583406_245_SCT	17,096,475	Atlantic - Scotland
H612	*Ssa27*	ctg7180001210476_199_SGT	17,100,902	Atlantic - Scotland
H613	*Ssa27*	ctg7180001213070_591_SCT	17,106,340	Atlantic - Scotland
H614	*Ssa27*	ctg7180001890551_1421_SCT	17,114,269	Atlantic - Scotland
H615	*Ssa27*	ctg7180001890551_1096_SAC	17,114,594	Atlantic& Cantabric - Scotland
H616	*Ssa27*	ctg7180001890551_1036_SAG	17,114,654	Atlantic& Cantabric - Scotland
H617	*Ssa27*	ctg7180001228300_461_SAC	17,121,458	Atlantic - Scotland
H618	*Ssa27*	ctg7180001436526_972_SAC	17,129,093	Atlantic - Scotland
H619	*Ssa27*	ctg7180001879469_2220_SCT	17,135,671	Atlantic - Scotland
H620	*Ssa27*	ctg7180001879469_6161_SCT	17,139,612	Atlantic - Scotland
H621	*Ssa27*	ctg7180001879469_6524_SCT	17,139,975	Atlantic - Scotland
H622	*Ssa27*	ctg7180001817910_12905_SCT	23,721,355	Cantabric - Scotland
H623	*Ssa28*	ctg7180001919998_1589_SCT	5,208,704	Atlantic - Scotland
H624	*Ssa28*	ctg7180001854384_8001_SAC	7,152,154	Cantabric - Scotland
H625	*Ssa28*	ctg7180001854384_7628_SGT	7,152,527	Cantabric - Scotland
H626	*Ssa28*	ctg7180001854384_6223_SGT	7,153,932	Cantabric - Scotland
H627	*Ssa28*	ctg7180001913270_16239_SCT	7,162,784	Cantabric - Scotland
H628	*Ssa29*	ctg7180001788132_886_SCT	31,989,744	Atlantic - Scotland
H629	*Ssa29*	ctg7180001788132_2146_SGT	31,991,045	Atlantic - Scotland
H630	*Ssa29*	ctg7180001337052_3998_SCT	32,047,897	Cantabric - Scotland

Table S2. Methods corresponding to the significant SNPs in Table S1 (significant for at least two haplotype-based methods). An asterisk indicates that the corresponding method detected the SNP as significant (SF: SmileFinder; sel: selscan).

**Crom.**	**ID**	**nvd**	**SF**	**sel**	**EOS**	**Number**
Ssa01	H1 - H9, H13 - H14	-	*	*	-	2
Ssa01	H10 - H12, H15 - H17, H25 - H28	*	*	-	-	2
Ssa01	H18 - H24, H29 - H30	*	-	*	-	2
Ssa01	H31 - H32	*	-	*	*	3
Ssa03	H33 - H36	-	*	*	-	2
Ssa03	H37, H44 - H51	*	-	*	-	2
Ssa03	H38 - H43	*	*	-	-	2
Ssa04	H52 - H65	-	*	*	-	2
Ssa04	H66 - H69, H84, H92 - H95	*	-	*	-	2
Ssa04	H70 - H91	*	-	*	*	3
Ssa05	H96 - H104	*	-	*	-	2
Ssa06	H105 - H115	-	*	*	-	2
Ssa07	H116 - H124, H133 - H134	*	*	-	-	2
Ssa07	H125, H132	-	*	*	-	2
Ssa07	H126 - H131	*	-	*	-	2
Ssa08	H135	*	-	*	-	2

Ssa09	H136 - H159, H224 - H225	*	-	*	-	2
Ssa09	H160, H165, H167, H169 - H173, H176 - H180, H187, H195, H198 - H221, H223	-	*	*	-	2
Ssa09	H161 - H164, H166, H168, H174 - H175, H181 - H186, H188 - H194, H196 - H197	*	*	*	-	3
Ssa09	H222	*	*	-	-	2
Ssa10	H226	*	*	-	*	3
Ssa10	H227 - H234	*	*	-	-	2
Ssa10	H235 - H240	*	-	*	-	2
Ssa11	H241 - H251	*	*	-	-	2
Ssa11	H252 - H253	*	*	*	-	3
Ssa11	H254, H257, H260, H273 - H274, H276 - H278, H280, H283 - H296, H303, H311 - H312, H314 - H323, H331 - H335	-	*	*	-	2
Ssa11	H255 - H256, H259, H261 - H272, H275, H279, H281 - H282, H297 - H302, H304 - H310, H313, H324 - H325	*	*	*	-	3
Ssa11	H326 - H330	*	-	*	-	2
Ssa12	H336, H338	*	-	*	-	2
Ssa12	H337	*	-	*	*	3
Ssa13	H339 - H340	*	*	-	-	2
Ssa14	H341 - H369	*	-	*	-	2
Ssa15	H370 - H371	*	*	-	-	2
Ssa15	H372 - H377	*	-	*	-	2
Ssa16	H378 - H385	*	-	*	-	2
Ssa16	H386 - H405	-	*	*	-	2
Ssa17	H406 - H407	*	-	*	-	2
Ssa17	H408 - H413, H418 - H426	*	*	-	-	2
Ssa17	H414 - H417, H427	-	*	*	-	2
Ssa18	H428	*	*	-	-	2
Ssa18	H429 - H430	*	-	*	*	3
Ssa18	H431	*	-	*	-	2
Ssa19	H432	*	-	*	*	3
Ssa19	H433 - H436	*	-	*	-	2
Ssa20	H437 - H438, H440	*	-	*	-	2
Ssa20	H439, H441 - H443	*	*	-	-	2
Ssa21	H444 - H445	*	*	-	-	2
Ssa21	H446 - H449	-	*	*	-	2
Ssa21	H450 - H452	*	-	*	-	2
Ssa22	H453	*	-	*	*	3
Ssa22	H454 - H473	*	-	*	-	2
Ssa24	H474 - H477, H485	*	-	*	-	2
Ssa24	H478, H480 - H538, H540 - H541	-	*	*	-	2
Ssa24	H479, H488	*	*	*	-	3
Ssa24	H539, H543 - H592	*	*	-	-	2
Ssa25	H593 - H594	-	*	*	-	2
Ssa25	H595 - H598	*	*	-	-	2
Ssa26	H599	*	-	*	-	2
Ssa26	H600 - H601	*	*	-	-	2
Ssa27	H602 - H603	*	*	-	-	2
Ssa27	H604 - H614, H617 - H621	-	*	*	-	2

Ssa27	H615 - H616	*	*	*	-	3
Ssa27	H622	*	-	*	-	2
Ssa28	H623	*	*	-	-	2
Ssa28	H624 - H627	*	-	*	-	2
Ssa29	H628 - H630	*	-	*	-	2

Table S3. Candidate genes, corresponding significant SNPs and gene ontology (GO).

**Chromosome**	**SNP ID (Table S1)**	**Name**	**Alias**	**GO term**
Ssa01	H1 - H9	CIGSSA_002336	*ldlrap1-b*	Biological process
Ssa01	H10 - H11	CIGSSA_002782	*lrrfip2*	Metabolism.
Ssa01	H12	CIGSSA_002790	*inpp5f*	Localization.
Ssa01	H13 - H17	CIGSSA_005109	*csgalnact2*	Development. Metabolism.
Ssa01	H18 - H23	CIGSSA_003292	*csmd1*	Multicellular organic processes. Regulation.
Ssa01	H25	CIGSSA_003864	*astn2*	Development. Localization.
Ssa01	H26	CIGSSA_003911	*tmem132c*	Cellular anatomical entity. Cellular process.
Ssa01	H29 - H32	CIGSSA_004177	*nek6*	Cellular process.
Ssa03	H33 - H35	CIGSSA_016245	*tpr*	Cellular component biogenesis. Localization. Metabolic process. Cellular process. Negative regulation of biological process. Response to stimulus.
Ssa03	H37	CIGSSA_016346	*dip2c*	Metabolic process.
Ssa03	H38	CIGSSA_016961	*psmd3*	Behaviour. Development. Metabolic process. Regulation of molecular function.
Ssa03	H39	CIGSSA_018885	*pdk2*	Development. Metabolic process. Cellular process. Regulation. Response to stimulus.
Ssa03	H43	CIGSSA_018973	*ccdc43*	-
Ssa03	H44 - H50	CIGSSA_018998	*ttyh3a*	Cellular anatomical entity. Localization
Ssa04	H52 - H54	CIGSSA_022723	*spns2*	Development. Localization. Cellular process. Regulation.
Ssa04	H59 - H63	CIGSSA_024135	*abcg4b*	Localization.
Ssa04	H64 - H65	CIGSSA_022727	*c2cd2*	Cellular anatomical entity.
Ssa05	H100 - H103	CIGSSA_028974	*pitx2*	Development. Metabolic process. Cellular process.
Ssa06	H107 - H115	CIGSSA_033021	*cep85l*	-
Ssa07	H125	CIGSSA_037981	*frrs1*	Metabolic process.
Ssa07	H126	CIGSSA_037982	*pawr*	Cellular process. Multicellular organismal process.

Ssa07	H132 - H133	CIGSSA_037983	*syt1*	Cellular component biogenesis. Behaviour. Development. Localization. Cellular process. Regulation.
Ssa09	H136	CIGSSA_045014	*daam2*	Cellular component biogenesis. Cellular anatomical entity. Cellular process.
Ssa09	H137	CIGSSA_045016	*ppp1r13b*	Cellular process.
Ssa09	H138	CIGSSA_043221	*limch1*	Cellular process.
Ssa09	H140 - H142	CIGSSA_043227	*fst15*	Cellular anatomical entity.
Ssa09	H157	CIGSSA_045324	*npm1*	Cellular component biogenesis. Development. Localization. Metabolic process. Cellular process. T cell proliferation. Regulation. Positive regulation of biological process. Response to stimulus.
Ssa09	H158	CIGSSA_045325	*tlx2*	Development. Metabolic process.
Ssa09	H170 - H173	CIGSSA_045334	*ankrd50*	Localization.
Ssa09	H177 - H188	CIGSSA_043270	*fat4*	Biological adhesion. Development.
Ssa09	H209 - H212	CIGSSA_043276	*pcgf3*	Development. Metabolic process.
Ssa09	H222	CIGSSA_043278	*smu1*	Cellular component biogenesis. Metabolic process. Cellular process.
Ssa09	H223	CIGSSA_043279	*tbc1d2*	Cellular component biogenesis. Localization. Regulation.
Ssa09	H224	CIGSSA_044147	*cct6a*	Protein complex. Cellular process.
Ssa09	H225	CIGSSA_046259	*cltcb*	Localization. Developmental process.
Ssa10	H228 - H229	CIGSSA_051679	*tab3*	Cellular process.
Ssa10	H231	CIGSSA_051680	*fam150a*	Development. Cellular process.
Ssa10	H233	CIGSSA_050022	*obscna*	Development. Cellular process.
Ssa10	H234	CIGSSA_050051	*fam163a*	-
Ssa10	H235 - H236	CIGSSA_050714	*osbpl5*	Localization.
Ssa10	H237	CIGSSA_052850	*nup205*	Cellular component biogenesis. Cellular process.
Ssa10	H239	CIGSSA_051263	*atp6v1e1b*	Development. Localization. Response to stress.
Ssa11	H256 - H264	CIGSSA_057338	*wdr43*	Cellular component biogenesis. Development.

				Localization. Regulation. Reproducción. Immune system process.
Ssa11	H269	CIGSSA_055817	*trmt61b*	Binding. Cellular process.
Ssa11	H270 - H276	CIGSSA_055818	*aats-glupro*	Protein complex.
Ssa11	H281	CIGSSA_055820	*glo1*	Development. Metabolic process. Cellular process. Immune system process.
Ssa11	H295 - H300	CIGSSA_055823	*zfand3*	Binding.
Ssa11	H304 - H325	CIGSSA_055824	*mdga1*	Cellular component biogenesis. Development.
Ssa11	H326	CIGSSA_055831	*syt24*	-
Ssa11	H328 - H330	CIGSSA_055848	*nell1*	Cellular component biogenesis. Cellular anatomical entity. Metabolic process. Cellular process. Multicellular organismal process. T cell proliferation.
Ssa11	H334 - H335	CIGSSA_056993	*dlg4*	Cellular component biogenesis. Behaviour. Development. Localization. Cellular process. Multicellular organismal process. Regulation. Response to stimulus.
Ssa12	H337	CIGSSA_063584	*pltp*	Metabolic process. Multicellular organismal process.
Ssa12	H338	CIGSSA_061951	*pcif1*	Cellular process.
Ssa13	H339 - H340	CIGSSA_067684	*arghap32*	Cellular anatomical entity.
Ssa14	H346	CIGSSA_075128	*s10ae*	Localization.
Ssa14	H366	CIGSSA_075129	*sirpa*	Biological adhesion. Cellular component biogenesis. Localization. Cellular process. Immune system process.
Ssa14	H367 - H368	CIGSSA_075230	*thrb*	Development.
Ssa15	H372 - H373	CIGSSA_080580	*espn*	Cellular process.
Ssa15	H375 - H376	CIGSSA_080784	*hdac7*	Development. Metabolic process.
Ssa15	H377	CIGSSA_079459	*plch2*	Metabolic process. Cellular process.
Ssa16	H385	CIGSSA_085229	*mef2a*	Cellular component biogenesis. Development. Localization. Metabolic process. Cellular process. Multicellular organismal process.
Ssa16	H386 - H388	CIGSSA_085235	*calb2*	Regulation.

Ssa16	H389 - H400	CIGSSA_083859	*hydin*	Cellular process.
Ssa16	H401 - H402	CIGSSA_083860	*necab2*	Cellular process.
Ssa16	H403 - H404	CIGSSA_085238	*pskh1*	Cellular process.
Ssa17	H406 - H407	CIGSSA_088719	*cpb2*	Metabolic process. Cellular process. Response to stress.
Ssa17	H408	CIGSSA_088733	*gsk3b*	Development. Cellular process.
Ssa17	H409	CIGSSA_089957	*trappc10*	Localization.
Ssa17	H418 - H419	CIGSSA_088840	*casq2*	Cellular component biogenesis. Development. Localization. Cellular process. Multicellular organismal process. Regulation. Response to stimulus.
Ssa17	H421 - H422	CIGSSA_090047	*vangl1*	Development. Cellular anatomical entity.
Ssa17	H423	CIGSSA_090048	*vangl2*	Cellular component biogenesis. Development. Cellular anatomical entity. Localization. Cellular process.
Ssa17	H424	CIGSSA_090049	*vangl3*	Cellular anatomical entity.
Ssa17	H425 - H426	CIGSSA_090050	*vangl4*	Cellular anatomical entity.
Ssa18	H428	CIGSSA_092658	*pprc1*	Metabolic process. Cellular process. Positive regulation of biological process.
Ssa18	H429 - H431	CIGSSA_093469	*pfdn2*	Cellular process.
Ssa19	H433	CIGSSA_096823	*lrit1a*	-
Ssa20	H439	CIGSSA_102235	*card9*	Cellular process.
Ssa20	H440	CIGSSA_101013	*sgsm1*	Cellular component biogenesis. Localization. Metabolic process. Regulation.
Ssa20	H441	CIGSSA_101587	*zgc:158328*	-
Ssa20	H442 - H443	CIGSSA_102872	*mecom*	Development. Metabolic process.
Ssa21	H444	CIGSSA_105802	*scn2a*	Binding. Development. Localization. Cellular process. Regulation.
Ssa21	H445	CIGSSA_105808	*b3galt1*	Metabolic process.
Ssa21	H446 - H449	CIGSSA_105813	*col28a1a*	-
Ssa21	H450	CIGSSA_106830	*clasp1*	Biological adhesion. Cellular component biogenesis. Development. Localization. Cellular process. Multicellular organismal process.
Ssa21	H452	CIGSSA_106235	*farp1*	Cellular anatomical entity. Cellular process.
Ssa22	H453 - H457	CIGSSA_108521	*ccdc3*	Cellular component

				biogenesis. Metabolic process.
Ssa22	H460 - H461	CIGSSA_109442	*kcc1a*	-
Ssa22	H462	CIGSSA_108522	*nek4*	Cellular process.
Ssa22	H471	CIGSSA_109445	*phc2b*	-
Ssa22	H472 - H473	CIGSSA_108626	*eefsec*	Metabolic process.
Ssa24	H474	CIGSSA_114324	*hps4*	Cellular component biogenesis. Cellular anatomical entity. Localization. Pigmentation. Cellular process. Regulation. Response to stress.
Ssa24	H475	CIGSSA_115013	*slc25a25*	Localization.
Ssa24	H478	CIGSSA_114350	*ppp6c*	Response to stress.
Ssa24	H480 - H482	CIGSSA_114351	*golga1*	Localization.
Ssa24	H483 - H484	CIGSSA_114352	*rpl35*	Binding. Metabolic process.
Ssa24	H492	CIGSSA_115020	*ofml2a*	-
Ssa24	H493 - H499	CIGSSA_114353	*nr6a1a*	Development. Localization. Metabolic process. Cellular process. Response to stimulus.
Ssa24	H500 - H501	CIGSSA_115024	*adgrd2*	Cellular process.
Ssa24	H514	CIGSSA_115025	*lhx2*	Anatomical structure development. Metabolic process.
Ssa24	H515	CIGSSA_115026	*crb2a*	Biological adhesion. Development. Localization. Cellular process.
Ssa24	H517	CIGSSA_114356	*zgc:55461*	Cellular process. Multi-organism process.
Ssa24	H518	CIGSSA_115029	*bcl2l1*	Binding. Development. Cellular process. Regulation.
Ssa24	H519 - H520	CIGSSA_115033	*fkbp15*	Localization. Cellular process.
Ssa24	H539	CIGSSA_115870	*enp2*	Metabolic process.
Ssa24	H531 - H532	CIGSSA_114357	*nelfb*	Development. Localization. Regulation.
Ssa24	H533 - H541	CIGSSA_114360	*cacna1ba*	Cellular component biogenesis. Cellular process.
Ssa24	H546 - H564	CIGSSA_114431	*mfge8b*	Biological adhesion.
Ssa24	H569 - H585	CIGSSA_114432	*hapln1*	Biological adhesion. Cellular component biogenesis. Development. Metabolic process. Cellular process. Multicellular organismal process. Response to

				stimulus.
Ssa24	H586 - H592	CIGSSA_115109	*vcan*	Biological adhesion. Development. Cellular process. Multicellular organismal process.
Ssa25	H593 - H594	CIGSSA_116392	*lrrfip1a*	Metabolic process.
Ssa25	H595 - H596	CIGSSA_116729	*mospd2*	-
Ssa25	H597	CIGSSA_116730	*gpr143*	Cellular process.
Ssa26	H599	CIGSSA_119826	*prmt3*	Cellular process.
Ssa26	H601	CIGSSA_119296	*cyld*	Metabolic process. Development. Cellular process.
Ssa28	H623	CIGSSA_124485	*wapa1*	Intracellular component.
Ssa29	H628 - H630	CIGSSA_126794	*mtrr*	Metabolic process.

Table S4. SNPs in chromosome *Ssa25*.

Id	SNP	BP
25_0	ctg7180001935593_3805_SCT	40203
25_1	ctg7180001855357_14284_SGT	50632
25_2	ctg7180001877470_10613_SCT	67559
25_3	ctg7180001877470_8497_SAC	69675
25_4	ctg7180001764704_504_SAC	89443
25_5	ctg7180001764704_74_SAC	89873
25_6	ctg7180001496214_1916_SCT	100308
25_7	ctg7180001877879_1317_SAG	174112
25_8	ctg7180001826470_6832_SCT	181905
25_9	ctg7180001837775_6202_SAC	248468
25_10	ctg7180001926624_1920_SAC	270664
25_11	ctg7180001926624_693_SAC	271891
25_12	ctg7180001936426_13596_SAG	279600
25_13	ctg7180001714329_1196_SGT	316193
25_14	ctg7180001932127_11853_SAG	323428
25_15	ctg7180001370096_954_SAC	359473
25_16	ctg7180001370096_1418_SAC	359937
25_17	ctg7180001370096_3431_SCT	361950
25_18	ctg7180001773173_1544_SCT	374530
25_19	ctg7180001804499_3552_SCT	379156
25_20	ctg7180001805524_6739_SCT	421269
25_21	ctg7180001805524_7463_SGT	421993
25_22	ctg7180001805524_8317_SAC	422847
25_23	ctg7180001805524_9011_SAG	423541
25_24	ctg7180001805524_9801_SAG	424331
25_25	ctg7180001805524_13771_SCT	428301
25_26	ctg7180001805524_16608_SGT	431138
25_27	ctg7180001764591_2672_SAC	441116
25_28	ctg7180001932318_10112_SGT	462904
25_29	ctg7180001890657_9608_SCT	475098
25_30	ctg7180001890657_835_SAC	483871
25_31	ctg7180001490221_1801_SGT	484975

25_32	ctg7180001615657_277_SCT	487036
25_33	ctg7180001833143_5424_SCT	495903
25_34	ctg7180001339448_2744_SGT	513300
25_35	ctg7180001891049_10685_SAG	544876
25_36	ctg7180001557515_393_SCT	545814
25_37	ctg7180001911356_10126_SGT	562519
25_38	ctg7180001911356_12608_SAC	565001
25_39	ctg7180001234870_2258_SGT	610446
25_40	ctg7180001709308_265_SCT	639308
25_41	ctg7180001709308_103_SCT	639470
25_42	ctg7180001374476_734_SCT	640743
25_43	ctg7180001883172_3488_SCT	651177
25_44	ctg7180001488992_11478_SCT	720052
25_45	ctg7180001872751_6149_SAG	726530
25_46	ctg7180001501644_3619_SAC	741429
25_47	ctg7180001794297_12438_SGT	758409
25_48	ctg7180001794297_12291_SGT	758556
25_49	ctg7180001794297_9805_SAG	761042
25_50	ctg7180001795472_13025_SAC	774453
25_51	ctg7180001871227_517_SCT	791189
25_52	ctg7180001871226_16417_SCT	793799
25_53	ctg7180001871226_13024_SCT	797192
25_54	ctg7180001871226_10403_SAG	799813
25_55	ctg7180001871225_4858_SAG	821223
25_56	ctg7180001871225_3110_SGT	822971
25_57	ctg7180001858998_1423_SCT	881883
25_58	ctg7180001858998_3454_SAG	883914
25_59	ctg7180001428206_6299_SAG	899025
25_60	ctg7180001226125_1390_SAG	901905
25_61	ctg7180001226125_1983_SCT	902230
25_62	ctg7180001226125_813_SAG	903400
25_63	ctg7180001226125_690_SCT	903523
25_64	ctg7180001226125_484_SGT	903729
25_65	ctg7180001900984_7602_SGT	906472
25_66	ctg7180001900984_7401_SAG	906673

25_67	ctg7180001900984_6732_SAG	907342
25_68	ctg7180001900984_5272_SAG	908802
25_69	ctg7180001900984_2074_SAG	912000
25_70	ctg7180001898685_682_SCT	914548
25_71	ctg7180001898685_4929_SAG	918795
25_72	ctg7180001898685_13191_SCT	927057
25_73	ctg7180001898685_17511_SCT	931377
25_74	ctg7180001918913_4985_SAC	951691
25_75	ctg7180001918913_3383_SAG	953293
25_76	ctg7180001886069_6600_SCT	958576
25_77	ctg7180001814145_749_SGT	968451
25_78	ctg7180001814145_1197_SCT	968899
25_79	ctg7180001814145_3951_SCT	971653
25_80	ctg7180001814145_4131_SCT	971833
25_81	ctg7180001814145_4468_SAC	972170
25_82	ctg7180001814145_7361_SAC	975063
25_83	ctg7180001915746_2340_SCT	994752
25_84	ctg7180001822550_2417_SAC	1370599
25_85	ctg7180001886582_954_SCT	1543728
25_86	ctg7180001832349_7185_SAG	1635925
25_87	ctg7180001287511_1263_SGT	1644437
25_88	ctg7180001742262_357_SAG	1666648
25_89	ctg7180001886963_1717_SCT	1671066
25_90	ctg7180001886963_2080_SAC	1671429
25_91	ctg7180001871095_56_SAC	1735994
25_92	ctg7180001871095_1439_SAG	1737377
25_93	ctg7180001871095_2199_SCT	1738172
25_94	ctg7180001929449_3300_SCT	1749509
25_95	ctg7180001325578_800_SCT	1767418
25_96	ctg7180001658479_531_SAG	1782949
25_97	ctg7180001904413_305_SAG	1786421
25_98	ctg7180001903113_1550_SAG	1807164
25_99	ctg7180001811099_299_SAC	1857091
25_100	ctg7180001811099_864_SGT	1857656
25_101	ctg7180001881179_5171_SGT	1898257

25_102	ctg7180001881179_5311_SAC	1898397
25_103	ctg7180001547313_547_SAG	1920679
25_104	ctg7180001817734_500_SCT	1931303
25_105	ctg7180001900935_1854_SCT	1947697
25_106	ctg7180001900935_7501_SAG	1953344
25_107	ctg7180001918404_14204_SCT	1966129
25_108	ctg7180001918404_2349_SAG	1977984
25_109	ctg7180001290487_2691_SCT	1987177
25_110	ctg7180001800687_3254_SAG	2013408
25_111	ctg7180001339452_13356_SAG	2017838
25_112	ctg7180001717994_9199_SCT	2035385
25_113	ctg7180001717994_8748_SAG	2035836
25_114	ctg7180001717994_6413_SAG	2038171
25_115	ctg7180001717994_5832_SAG	2038752
25_116	ctg7180001717994_2906_SAC	2041678
25_117	ctg7180001873181_1683_SGT	2052584
25_118	ctg7180001440554_1293_SAC	2055199
25_119	ctg7180001901671_5129_SCT	2064514
25_120	ctg7180001789746_576_SGT	2069076
25_121	ctg7180001820983_1044_SCT	2077918
25_122	ctg7180001820983_1482_SAC	2078356
25_123	ctg7180001827118_4677_SGT	2126484
25_124	ctg7180001902892_11036_SAG	2170372
25_125	ctg7180001902892_11218_SGT	2170554
25_126	ctg7180001817801_6204_SCT	2209383
25_127	ctg7180001917382_3952_SGT	2220242
25_128	ctg7180001910534_11503_SAG	2229425
25_129	ctg7180001910534_11474_SAC	2229454
25_130	ctg7180001910534_11321_SAC	2229607
25_131	ctg7180001910534_11209_SCT	2229719
25_132	ctg7180001904850_3009_SGT	2249819
25_133	ctg7180001927082_10542_SAG	2318725
25_134	ctg7180001927082_7529_SAG	2321684
25_135	ctg7180001286864_1966_SCT	2354974
25_136	ctg7180001286864_2454_SAG	2355462

25_137	ctg7180001875250_2781_SAC	2387434
25_138	ctg7180001875250_2951_SAG	2387604
25_139	ctg7180001875250_5562_SGT	2390215
25_140	ctg7180001883843_8792_SAG	2415600
25_141	ctg7180001549796_1880_SCT	2424778
25_142	ctg7180001549796_6484_SCT	2429382
25_143	ctg7180001805637_4034_SGT	2539703
25_144	ctg7180001805637_3423_SCT	2540314
25_145	ctg7180001805637_3278_SCT	2540459
25_146	ctg7180001490778_370_SAC	2548288
25_147	ctg7180001871596_10919	2572762
25_148	ctg7180001829711_831_SAG	2584792
25_149	ctg7180001829711_886_SCT	2584847
25_150	ctg7180001872191_7613_SGT	2608865
25_151	ctg7180001214221_4284_SAG	2649673
25_152	ctg7180001692300_409_SAG	2677776
25_153	ctg7180001692300_1475_SCT	2678842
25_154	ctg7180001692300_4475_SCT	2681842
25_155	ctg7180001839087_612_SGT	2727442
25_156	ctg7180001839087_961_SAG	2727791
25_157	ctg7180001763875_1707_SGT	2737167
25_158	ctg7180001868897_7728_SAG	2750567
25_159	ctg7180001868897_2174_SAC	2756121
25_160	ctg7180001910905_1337_SCT	2774019
25_161	ctg7180001910905_2259_SAC	2774941
25_162	ctg7180001910905_2964_SCT	2775646
25_163	ctg7180001910905_8823_SAG	2781505
25_164	ctg7180001910905_18840_SAC	2791522
25_165	ctg7180001910534_2984_SCT	2800113
25_166	ctg7180001820176_6140_SAG	2817561
25_167	ctg7180001228299_4344_SAC	2828121
25_168	ctg7180001228299_6174_SAG	2829951
25_169	ctg7180001846957_7799_SCT	2855830
25_170	ctg7180001850041_13884_SAG	2890250
25_171	ctg7180001850041_10004_SGT	2894130

25_172	ctg7180001931081_2185_SGT	2933125
25_173	ctg7180001931081_2872_SCT	2933812
25_174	ctg7180001931081_3223_SAC	2934163
25_175	ctg7180001931081_4720_SGT	2935660
25_176	ctg7180001931081_6512_SCT	2937452
25_177	ctg7180001901727_2759_SAC	2990508
25_178	ctg7180001935758_2726_SAG	3002167
25_179	ctg7180001507916_1359_SAG	3065430
25_180	ctg7180001841444_5634_SCT	3072255
25_181	ctg7180001345614_2505_SAG	3088526
25_182	ctg7180001924168_185_SAG	3097815
25_183	ctg7180001915454_1245_SCT	3119557
25_184	ctg7180001805729_5017_SCT	3124845
25_185	ctg7180001861707_14025_SCT	3133435
25_186	ctg7180001861707_13603_SCT	3133857
25_187	ctg7180001861707_11802_SAG	3135658
25_188	ctg7180001861707_11757_SAG	3135703
25_189	ctg7180001876896_4415_SAG	3225640
25_190	ctg7180001244499_1945_SCT	3237519
25_191	ctg7180001869686_6066_SAG	3295635
25_192	ctg7180001919467_1821_SAG	3348902
25_193	ctg7180001884727_2152_SAG	3377299
25_194	ctg7180001916740_552_SCT	3508536
25_195	ctg7180001916740_601_SCT	3508585
25_196	ctg7180001916740_8079_SAG	3516063
25_197	ctg7180001452310_421_SCT	3533346
25_198	ctg7180001669659_1323_SAC	3553048
25_199	ctg7180001589299_335_SCT	3555749
25_200	ctg7180001492932_6126_SAG	3564707
25_201	ctg7180001492932_3379_SGT	3567454
25_202	ctg7180001861920_5630_SCT	3580304
25_203	ctg7180001810033_732_SGT	3613942
25_204	ctg7180001900606_4654_SCT	3663420
25_205	ctg7180001893046_13772_SGT	3708514
25_206	ctg7180001645886_1219_SAC	3724425

25_207	ctg7180001454729_3659_SGT	3751169
25_208	ctg7180001804470_10163_SAG	3762058
25_209	ctg7180001914198_1339_SAC	3780470
25_210	ctg7180001915823_11026_SAG	3801190
25_211	ctg7180001915823_11330_SGT	3801494
25_212	ctg7180001915823_11364_SGT	3801528
25_213	ctg7180001891582_4194_SCT	3827188
25_214	ctg7180001901921_3973_SAC	3866966
25_215	ctg7180001880024_5101_SAC	3954961
25_216	ctg7180001880024_4634_SAC	3955428
25_217	ctg7180001880024_4342_SGT	3955720
25_218	ctg7180001933032_19790_SAG	3973022
25_219	ctg7180001933032_18791_SAC	3974021
25_220	ctg7180001933032_8676_SCT	3984136
25_221	ctg7180001824328_2983_SCT	4055785
25_222	ctg7180001824328_5583_SAG	4058422
25_223	ctg7180001824328_11067_SCT	4063870
25_224	ctg7180001898803_4794_SAG	4104275
25_225	ctg7180001898803_3446_SGT	4105623
25_226	ctg7180001242022_2228_SAG	4117809
25_227	ctg7180001931398_17043_SGT	4123300
25_228	ctg7180001388717_3206_SGT	4138862
25_229	ctg7180001876853_2669_SGT	4161325
25_230	ctg7180001935278_3613_SCT	4187950
25_231	ctg7180001834284_4057_SAC	4214026
25_232	ctg7180001834284_767_SGT	4217316
25_233	ctg7180001834284_732_SAC	4217351
25_234	ctg7180001834284_328_SAG	4217755
25_235	ctg7180001816812_16119_SAC	4252370
25_236	ctg7180001842599_3140_SCT	4273168
25_237	ctg7180001842599_3820_SCT	4273848
25_238	ctg7180001842599_5951_SCT	4275979
25_239	ctg7180001794302_4121_SGT	4326746
25_240	ctg7180001909521_6667_SGT	4342222
25_241	ctg7180001909521_6711_SCT	4342266

25_242	ctg7180001909521_6920_SGT	4342475
25_243	ctg7180001909521_11955_SAG	4347510
25_244	ctg7180001928602_14997_SGT	4423806
25_245	ctg7180001928602_14905_SAC	4423898
25_246	ctg7180001928602_13705_SGT	4425098
25_247	ctg7180001928602_13540_SCT	4425263
25_248	ctg7180001921938_4561_SAC	4536848
25_249	ctg7180001881900_16683_SAC	4574508
25_250	ctg7180001881900_15008_SAC	4576183
25_251	ctg7180001881900_14971_SCT	4576220
25_252	ctg7180001881900_14502_SAC	4576689
25_253	ctg7180001881900_10144_SCT	4581047
25_254	ctg7180001881900_8805_SAG	4582386
25_255	ctg7180001788314_3766_SCT	4638396
25_256	ctg7180001788314_3174_SAG	4638988
25_257	ctg7180001788314_316_SCT	4641846
25_258	ctg7180001937184_33332_SAG	4663094
25_259	ctg7180001937184_564_SCT	4697705
25_260	ctg7180001804479_4304_SGT	4702624
25_261	ctg7180001907427_9929_SCT	4729039
25_262	ctg7180001852893_3473_SAC	4740908
25_263	ctg7180001937542_21700_SCT	4760675
25_264	ctg7180001937542_21396_SAG	4760979
25_265	ctg7180001937542_18096_SCT	4764279
25_266	ctg7180001937542_17814_SCT	4764561
25_267	ctg7180001937542_17680_SGT	4775779
25_268	ctg7180001937542_5324_SCT	4777051
25_269	ctg7180001937542_4814_SAC	4777561
25_270	ctg7180001937540_15596_SAG	4900891
25_271	ctg7180001937540_13874_SAC	4902614
25_272	ctg7180001937540_8867_SAG	4907622
25_273	ctg7180001937540_3950_SCT	4912539
25_274	ctg7180001892317_7435_SAG	4921779
25_275	ctg7180001892317_667_SCT	4928547
25_276	ctg7180001636798_1175_SCT	4930575

25_277	ctg7180001636798_9909_SCT	4939309
25_278	ctg7180001865629_5400_SGT	4949289
25_279	ctg7180001865629_7700_SGT	4951589
25_280	ctg7180001865629_11397_SAC	4955286
25_281	ctg7180001865629_11766_SAG	4955655
25_282	ctg7180001865629_18481_SAG	4962370
25_283	ctg7180001865629_18685_SCT	4962574
25_284	ctg7180001865629_18772_SAG	4962661
25_285	ctg7180001865629_28313_SAG	4972202
25_286	ctg7180001865629_36058_SGT	4979947
25_287	ctg7180001800511_2003_SCT	4982761
25_288	ctg7180001800511_6383_SCT	4987141
25_289	ctg7180001670914_3795_SAG	4991280
25_290	ctg7180001895082_16635_SAG	5011796
25_291	ctg7180001895082_18235_SAG	5013396
25_292	ctg7180001895082_18688_SAG	5013849
25_293	ctg7180001904766_32348_SAC	5025152
25_294	ctg7180001904766_30100_SAG	5027400
25_295	ctg7180001904766_655_SAC	5056847
25_296	ctg7180001651772_1057_SCT	5064594
25_297	ctg7180001615140_3380_SAC	5069207
25_298	ctg7180001307690_5822_SGT	5080396
25_299	ctg7180001307690_1582_SAG	5080818
25_300	ctg7180001307690_2470_SAG	5083748
25_301	ctg7180001917075_5647_SAC	5097538
25_302	ctg7180001917075_1750_SAG	5101435
25_303	ctg7180001813548_3738_SCT	5109874
25_304	ctg7180001813548_4633_SCT	5110769
25_305	ctg7180001813548_4715_SCT	5110851
25_306	ctg7180001813548_5334_SAG	5111470
25_307	ctg7180001835918_5791_SGT	5123007
25_308	ctg7180001835918_10870_SAC	5128086
25_309	ctg7180001815641_664_SCT	5170382
25_310	ctg7180001815641_1553_SAC	5171271
25_311	ctg7180001815641_1722 5171475	

25_312	ctg7180001815641_2214_SCT	5171932
25_313	ctg7180001815641_3086_SAG	5172804
25_314	ctg7180001815641_4803_SAG	5174521
25_315	ctg7180001803192_12815_SAC	5202280
25_316	ctg7180001803192_12996_SAC	5202461
25_317	ctg7180001360769_758_SGT	5209089
25_318	ctg7180001285088_8333	5219484
25_319	ctg7180001805892_1326_SAC	5288298
25_320	ctg7180001594927_398_SGT	5292452
25_321	ctg7180001594927_436_SAC	5292490
25_322	ctg7180001594927_502_SCT	5292556
25_323	ctg7180001594927_915_SAC	5292969
25_324	ctg7180001893337_9135_SAG	5322780
25_325	ctg7180001900444_1184_SGT	5335957
25_326	ctg7180001831059_9237_SGT	5359063
25_327	ctg7180001831059_8345_SAG	5359955
25_328	ctg7180001814594_13956_SCT	5385988
25_329	ctg7180001368988_1799_SCT	5465152
25_330	GCR_cBin31585_Ctg1_216	5470730
25_331	ctg7180001650120_2144_SAG	5562143
25_332	ctg7180001923663_9757_SAC	5605835
25_333	ctg7180001798976_6581_SAG	5621039
25_334	ctg7180001798976_8463_SCT	5622921
25_335	ctg7180001908666_8331_SAC	5631683
25_336	ctg7180001896329_1754_SAG	5660907
25_337	ctg7180001927889_4720_SAG	5679139
25_338	ctg7180001916723_3879_SGT	5693424
25_339	ctg7180001856764_10656_SGT	5713867
25_340	ctg7180001856764_8886_SAG	5715637
25_341	ctg7180001211218_2037_SGT	5730531
25_342	ctg7180001211218_1799_SAG	5730769
25_343	ctg7180001844522_443_SCT	5733202
25_344	ctg7180001844522_4230_SAG	5736990
25_345	ctg7180001580412_296_SCT	5758329
25_346	ctg7180001297471_2066_SAG	5872148

25_347	ctg7180001288933_6554_SCT	5898240
25_348	ctg7180001789427_928_SAC	5945085
25_349	ctg7180001872131_6803_SAC	5961297
25_350	ctg7180001897430_6700_SAG	5977118
25_351	ctg7180001930313_4689_SAG	5991122
25_352	ctg7180001911838_4189_SAG	6076594
25_353	ctg7180001799086_4819_SGT	6093673
25_354	ctg7180001887537_8166_SAC	6189140
25_355	ctg7180001812156_2361_SCT	6215621
25_356	ctg7180001925613_5157_SGT	6250923
25_357	ctg7180001879684_5373_SAG	6282220
25_358	ctg7180001879684_5090_SCT	6282503
25_359	ctg7180001917261_10084_SAG	6319685
25_360	ctg7180001824312_729_SAG	6361775
25_361	ctg7180001816366_1061_SAG	6482133
25_362	ctg7180001539878_3877_SAC	6490173
25_363	ctg7180001539878_2671_SAG	6491379
25_364	ctg7180001539878_2342_SCT	6491708
25_365	ctg7180001895574_2747_SAG	6517587
25_366	ctg7180001895574_8794_SCT	6523599
25_367	ctg7180001895574_12728_SCT	6527533
25_368	ctg7180001895574_12898_SAG	6527703
25_369	ctg7180001895574_24741_SAG	6539546
25_370	ctg7180001453857_5532_SAG	6565850
25_371	ctg7180001453857_5252_SCT	6566130
25_372	ctg7180001453857_4516_SCT	6566866
25_373	ctg7180001814336_1520_SCT	6577827
25_374	ctg7180001361811_2292_SGT	6643502
25_375	ctg7180001361811_4429_SCT	6645639
25_376	ctg7180001855361_294_SCT	6660842
25_377	ctg7180001293420_2561_SGT	6665471
25_378	ctg7180001293420_2836_SAC	6665746
25_379	ctg7180001293420_3408_SCT	6666318
25_380	ctg7180001293420_4344_SCT	6667254
25_381	ctg7180001810041_7339_SCT	6673445

25_382	ctg7180001894108_650_SGT	6716677
25_383	ctg7180001894108_1383_SGT	6717410
25_384	ctg7180001894108_8191_SCT	6724218
25_385	ctg7180001894108_12271_SAG	6728298
25_386	ctg7180001894108_16489_SAC	6732516
25_387	ctg7180001483697_1422_SCT	6738542
25_388	ctg7180001812121_2475_SGT	6758497
25_389	ctg7180001797954_2453_SGT	6802546
25_390	ctg7180001840776_2128_SGT	6845116
25_391	ctg7180001840776_3174_SCT	6846162
25_392	ctg7180001909169_6561_SGT	6866045
25_393	ctg7180001936846_386_SCT	6893813
25_394	ctg7180001936848_1304_SCT	6972468
25_395	ctg7180001889517_9401_SAC	7019278
25_396	ctg7180001889517_1876_SCT	7026803
25_397	ctg7180001889517_1270_SAC	7027409
25_398	ctg7180001889517_1112_SAG	7027567
25_399	ctg7180001889517_1007_SAG	7027672
25_400	ctg7180001889517_748_SCT	7027931
25_401	ctg7180001895941_5314_SAC	7048022
25_402	ctg7180001895940_15003_SAG	7058649
25_403	ctg7180001895940_10255_SCT	7063397
25_404	ctg7180001895940_9243_SAG	7064409
25_405	ctg7180001895940_8055_SAC	7065597
25_406	ctg7180001895940_2665_SAG	7070987
25_407	ctg7180001602886_1614_SCT	7074446
25_408	ctg7180001929920_1022_SCT	7101120
25_409	ctg7180001849805_15379_SGT	7109056
25_410	ctg7180001836950_7814_SAC	7140426
25_411	ctg7180001694128_3601_SCT	7171067
25_412	ctg7180001293867_1362_SAG	7175025
25_413	ctg7180001293867_1308_SGT	7175079
25_414	ctg7180001293867_1285_SCT	7175102
25_415	ctg7180001293867_941_SGT	7175446
25_416	ctg7180001293867_2418_SCT	7176323

25_417	ctg7180001246245_6209_SCT	7177929
25_418	ctg7180001246245_3803_SGT	7180335
25_419	ctg7180001246245_3698_SCT	7180440
25_420	ctg7180001246245_2533_SCT	7181605
25_421	ctg7180001932392_547_SAG	7185227
25_422	ctg7180001809690_3042_SAG	7208541
25_423	ctg7180001809690_2910_SCT	7208673
25_424	ctg7180001933288_877_SCT	7267539
25_425	ctg7180001933288_745_SGT	7267671
25_426	ctg7180001933288_733_SAC	7267683
25_427	ctg7180001933288_113_SGT	7268303
25_428	ctg7180001320361_2012_SGT	7271634
25_429	ctg7180001320361_1965_SGT	7271681
25_430	ctg7180001320361_1738_SGT	7271908
25_431	ctg7180001320361_1352_SAC	7272294
25_432	ctg7180001320361_695_SCT	7272952
25_433	ctg7180001544433_2254_SGT	7290077
25_434	ctg7180001544433_8198_SAG	7296021
25_435	ctg7180001659268_7868_SGT	7307898
25_436	ctg7180001659268_7579_SAC	7308187
25_437	ctg7180001349252_565_SGT	7316305
25_438	ctg7180001349252_601_SAC	7316341
25_439	ctg7180001729866_1480_SAC	7320383
25_440	ctg7180001674422_2743_SCT	7350245
25_441	ctg7180001674422_2194_SCT	7350794
25_442	ctg7180001674422_2172_SAG	7350816
25_443	ctg7180001937991_7984_SCT	7361214
25_444	ctg7180001937991_8629_SAC	7361859
25_445	ctg7180001937991_8680_SAC	7361910
25_446	ctg7180001577012_2059_SAG	7402424
25_447	ctg7180001577012_708_SCT	7403775
25_448	ctg7180001628303_3218_SAG	7405034
25_449	ctg7180001628303_558_SGT	7407694
25_450	ctg7180001628303_285_SAG	7407967
25_451	ctg7180001787249_2581_SAG	7411791

25_452	ctg7180001934787_13500_SCT	7417339
25_453	ctg7180001891578_237_SAG	7433981
25_454	ctg7180001593785_2760_SGT	7455340
25_455	ctg7180001593785_879_SCT	7457221
25_456	ctg7180001821135_865_SCT	7469797
25_457	ctg7180001821135_831_SCT	7469831
25_458	ctg7180001216992_7013_SAG	7483722
25_459	ctg7180001912994_6720_SGT	7497502
25_460	ctg7180001912994_6771_SAG	7497553
25_461	ctg7180001912994_8701_SAG	7499483
25_462	ctg7180001913162_1601_SAG	7511985
25_463	ctg7180001913162_3479_SCT	7513863
25_464	ctg7180001913162_4022_SAC	7514406
25_465	ctg7180001913162_22489_SCT	7532873
25_466	ctg7180001913162_23291_SCT	7533675
25_467	ctg7180001913162_24586_SAC	7534970
25_468	ctg7180001913162_25423_SAC	7535807
25_469	ctg7180001913162_25669_SGT	7536053
25_470	ctg7180001386140_1423_SAG	7542548
25_471	ctg7180001386140_1351_SGT	7542620
25_472	ctg7180001326282_564_SAG	7548718
25_473	ctg7180001740542_2964_SAG	7552782
25_474	ctg7180001869786_6967_SAC	7569303
25_475	ctg7180001488958_1864_SAG	7582939
25_476	ctg7180001488958_1623_SAG	7583180
25_477	ctg7180001826187_4908_SAG	7586531
25_478	ctg7180001826187_4705_SAG	7586734
25_479	ctg7180001937488_2080_SCT	7593802
25_480	ctg7180001937488_3573_SAG	7595295
25_481	ctg7180001937488_5249_SCT	7596969
25_482	ctg7180001344083_2247_SGT	7602408
25_483	ctg7180001901783_6435_SAG	7621452
25_484	ctg7180001901783_9787_SCT	7624804
25_485	ctg7180001927162_2918_SAG	7642378
25_486	ctg7180001927162_19214_SGT	7658674

25_487	ctg7180001927162_19402_SCT	7658862
25_488	ctg7180001927162_19770_SAG	7659230
25_489	ctg7180001927162_19842_SCT	7659302
25_490	ctg7180001927162_21998_SAG	7661458
25_491	ctg7180001927162_22495_SAC	7661955
25_492	ctg7180001863418_13483_SAG	7666762
25_493	ctg7180001863418_11497_SGT	7668748
25_494	ctg7180001863418_8884_SGT	7671361
25_495	ctg7180001863418_8359_SAC	7671886
25_496	ctg7180001863418_8055_SAC	7672190
25_497	ctg7180001863418_6896_SAG	7673349
25_498	ctg7180001863418_5608_SAC	7674637
25_499	ctg7180001863418_5417_SAC	7674828
25_500	ctg7180001603500_622_SGT	7685070
25_501	ctg7180001824997_4490_SGT	7699363
25_502	ctg7180001824997_4409_SCT	7699444
25_503	ctg7180001496552_3822_SGT	7707435
25_504	ctg7180001496552_5011_SGT	7708589
25_505	ctg7180001496552_5760_SAC	7709338
25_506	ctg7180001496552_6223_SAC	7709801
25_507	ctg7180001496552_8073_SCT	7711651
25_508	ctg7180001496552_9613_SCT	7713191
25_509	ctg7180001820735_20945_SAC	7732578
25_510	ctg7180001820735_21665_SCT	7733298
25_511	ctg7180001820735_22425_SAG	7734058
25_512	ctg7180001820735_22023	7741514
25_513	ctg7180001299329_1388_SAC	7746267
25_514	ctg7180001913830_3355_SAG	7750631
25_515	ctg7180001913830_7669_SAC	7754945
25_516	ctg7180001913830_8964_SCT	7756240
25_517	ctg7180001913831_3376_SAG	7773754
25_518	ctg7180001913831_3602_SGT	7773980
25_519	ctg7180001913831_14563_SCT	7784941
25_520	ctg7180001913831_14817_SAG	7785195
25_521	ctg7180001700884_935_SCT	7810280

25_522	ctg7180001250815_7768_SCT	7818992
25_523	ctg7180001250815_9622_SAC	7820846
25_524	ctg7180001250815_10098_SAC	7821322
25_525	ctg7180001361536_1783_SGT	7829383
25_526	ctg7180001361536_954_SCT	7830212
25_527	ctg7180001919017_7901_SAG	7841867
25_528	ctg7180001907059_1921_SAG	7865280
25_529	ctg7180001886887_5783_SAG	7874935
25_530	ctg7180001477832_2889_SCT	7896965
25_531	ctg7180001877202_14790_SCT	7916087
25_532	ctg7180001892444_19191_SGT	7931082
25_533	ctg7180001892444_19179_SGT	7931094
25_534	ctg7180001892444_23100_SAC	7941649
25_535	ctg7180001661310_889_SCT	7953342
25_536	ctg7180001921885_1080_SGT	7972217
25_537	ctg7180001921885_1218_SAC	7972355
25_538	ctg7180001921885_7954_SGT	7979091
25_539	ctg7180001894387_5106_SCT	8008948
25_540	ctg7180001894387_3038_SCT	8011016
25_541	ctg7180001860204_1286_SAC	8127389
25_542	ctg7180001860204_2848_SAG	8128951
25_543	ctg7180001860204_36975_SAG	8163078
25_544	ctg7180001910774_7911_SAC	8188942
25_545	ctg7180001921815_7944_SAG	8208428
25_546	ctg7180001921815_7576_SAC	8208796
25_547	ctg7180001921815_7485_SCT	8208887
25_548	ctg7180001842434_4085_SAG	8255400
25_549	ctg7180001842434_2971_SAG	8256514
25_550	ctg7180001650895_1184_SAG	8261424
25_551	ctg7180001650895_2371_SAG	8262611
25_552	ctg7180001840167_5964_SCT	8293861
25_553	ctg7180001840167_6988_SAC	8294885
25_554	ctg7180001928793_12045_SAG	8327052
25_555	ctg7180001928793_11338_SCT	8327759
25_556	ctg7180001928793_5786_SAC	8333311

25_557	ctg7180001221724_5798_SAG	8341790
25_558	ctg7180001221724_5626_SAC	8341962
25_559	ctg7180001909594_3776_SAG	8354999
25_560	ctg7180001909594_12364_SAG	8363586
25_561	ctg7180001909594_12456_SCT	8363678
25_562	ctg7180001870044_17212_SAC	8391348
25_563	ctg7180001192072_1350_SAG	8427148
25_564	ctg7180001793547_4987_SAC	8434269
25_565	ctg7180001793547_6316_SAG	8435598
25_566	ctg7180001793547_7243_SGT	8436525
25_567	ctg7180001793547_7666_SAG	8436948
25_568	ctg7180001609599_10858_SAG	8438877
25_569	ctg7180001609599_5560_SCT	8444175
25_570	ctg7180001609599_5115_SAG	8444620
25_571	ctg7180001866255_18658_SAC	8451061
25_572	ctg7180001866255_18348_SAC	8451371
25_573	ctg7180001656204_6126_SCT	8477178
25_574	ctg7180001518674_190_SAG	8487789
25_575	ctg7180001907494_3657_SAC	8498913
25_576	ctg7180001907494_3591_SAG	8498979
25_577	ctg7180001484292_550_SCT	8504828
25_578	ctg7180001433617_4203_SAC	8539065
25_579	ctg7180001688315_1601_SGT	8555644
25_580	ctg7180001903203_7596_SAG	8609883
25_581	ctg7180001717584_3729_SGT	8637786
25_582	ctg7180001825551_9441_SAG	8656876
25_583	ctg7180001935619_7806_SCT	8675070
25_584	ctg7180001935619_753_SCT	8682123
25_585	ctg7180001257370_3015_SAG	8685166
25_586	ctg7180001257370_5194_SAG	8687345
25_587	ctg7180001257370_5230_SGT	8687416
25_588	ctg7180001257370_5997_SCT	8688148
25_589	ctg7180001257370_6557_SAG	8688708
25_590	ctg7180001351747_456_SCT	8703714
25_591	ctg7180001351747_1243_SCT	8704501

25_592	ctg7180001351747_1260_SCT	8704518
25_593	ctg7180001913751_13154_SGT	8718866
25_594	ctg7180001913751_7352_SAG	8724668
25_595	ctg7180001913751_1750_SCT	8730264
25_596	ctg7180001929917_2826_SGT	8736509
25_597	ctg7180001194812_1085_SGT	8749814
25_598	ctg7180001821918_1901_SCT	8773753
25_599	ctg7180001821918_3801_SAG	8775654
25_600	ctg7180001821918_3897_SAC	8775750
25_601	ctg7180001270031_1201_SAG	8826856
25_602	ctg7180001264182_1782_SAG	8838029
25_603	ctg7180001598829_2153_SCT	8860581
25_604	ctg7180001598829_1934_SCT	8860800
25_605	ctg7180001818955_5026_SCT	8886821
25_606	ctg7180001818955_4342_SAG	8887505
25_607	ctg7180001875571_257_SGT	8914204
25_608	ctg7180001875571_2428_SAC	8916375
25_609	ctg7180001450487_158_SAG	8933838
25_610	ctg7180001450487_348_SGT	8934028
25_611	ctg7180001450487_2653_SAG	8936333
25_612	ctg7180001450487_3232_SGT	8936912
25_613	ctg7180001491644_6913_SGT	8949336
25_614	ctg7180001491644_6839_SCT	8949410
25_615	ctg7180001526568_10004_SGT	8957808
25_616	ctg7180001526568_2020_SCT	8965792
25_617	ctg7180001886316_2524_SAG	8973956
25_618	ctg7180001807747_18321_SCT	8996017
25_619	ctg7180001825439_9546_SAG	9086839
25_620	ctg7180001737979_2208_SCT	9131638
25_621	ctg7180001836545_4079_SAG	9155894
25_622	ctg7180001795265_4858_SGT	9196407
25_623	ctg7180001795265_5360_SAG	9196909
25_624	ctg7180001213231_3056_SAC	9217501
25_625	ctg7180001821765_10341_SGT	9223405
25_626	ctg7180001821765_3051_SCT	9225155

25_627	ctg7180001821765_4554_SCT	9229194
25_628	ctg7180001821765_466_SCT	9233281
25_629	ctg7180001857836_2572_SGT	9239996
25_630	ctg7180001377987_1086_SAG	9254360
25_631	ctg7180001377987_1167_SGT	9254441
25_632	ctg7180001844787_3631_SCT	9263256
25_633	ctg7180001844787_4024_SAC	9263649
25_634	ctg7180001827334_2979_SAG	9281032
25_635	ctg7180001827334_3745_SCT	9281798
25_636	ctg7180001855009_4183_SGT	9297615
25_637	ctg7180001498532_6208_SAG	9299010
25_638	ctg7180001937900_29203_SAG	9316563
25_639	ctg7180001937900_29053_SCT	9316713
25_640	ctg7180001937900_28881_SAC	9316885
25_641	ctg7180001937900_28674_SCT	9317092
25_642	ctg7180001937900_28591_SGT	9317175
25_643	ctg7180001937900_28506_SAG	9317260
25_644	ctg7180001937900_28278_SCT	9317488
25_645	ctg7180001937900_26879_SAC	9318887
25_646	ctg7180001937900_22816_SAC	9322950
25_647	ctg7180001937900_18610_SAG	9327156
25_648	ctg7180001937900_16704_SAG	9329062
25_649	ctg7180001937900_16358_SAG	9329408
25_650	ctg7180001937900_22634_SGT	9337221
25_651	ctg7180001937900_7735_SGT	9338031
25_652	ctg7180001937900_6841_SCT	9338925
25_653	ctg7180001937900_6231_SCT	9339535
25_654	ctg7180001872385_256_SCT	9349127
25_655	ctg7180001913316_1787_SGT	9352804
25_656	ctg7180001685787_2689_SAC	9386686
25_657	ctg7180001816664_3453_SAG	9397789
25_658	ctg7180001784796_4551_SAG	9407545
25_659	ctg7180001784796_242_SAG	9411854
25_660	ctg7180001510359_114_SAC	9420883
25_661	ctg7180001867869_3525_SAC	9424113

25_662	ctg7180001867869_2742_SAC	9424896
25_663	ctg7180001820576_4391_SAC	9432077
25_664	ctg7180001700823_5642_SAC	9440061
25_665	ctg7180001700823_3576_SAG	9442127
25_666	ctg7180001700823_1995_SAC	9443708
25_667	ctg7180001700823_387_SAG	9445316
25_668	ctg7180001617142_1977_SAG	9474627
25_669	ctg7180001802600_1178_SAG	9492888
25_670	ctg7180001802600_1842_SAG	9493552
25_671	ctg7180001802600_4370_SCT	9496080
25_672	ctg7180001802600_4540_SAG	9496250
25_673	ctg7180001802600_4995_SCT	9496705
25_674	ctg7180001802600_5701_SAG	9497411
25_675	ctg7180001802600_6644_SAG	9498354
25_676	ctg7180001802600_7149_SAC	9498859
25_677	ctg7180001802600_8160_SCT	9499870
25_678	ctg7180001802600_8335_SAG	9500045
25_679	ctg7180001802600_9728_SCT	9501438
25_680	ctg7180001912390_364_SCT	9549739
25_681	ctg7180001934986_3650_SCT	9570741
25_682	ctg7180001934986_8817_SAG	9575908
25_683	ctg7180001934986_8996_SAC	9576087
25_684	ctg7180001934986_16127_SAC	9583220
25_685	ctg7180001934986_17345_SGT	9584438
25_686	ctg7180001287773_6302_SCT	9607428
25_687	ctg7180001889079_7502_SAG	9615586
25_688	ctg7180001615195_1234_SCT	9622992
25_689	ctg7180001615195_2400_SCT	9624158
25_690	ctg7180001615195_2755_SAC	9624513
25_691	ctg7180001876823_7670_SCT	9627351
25_692	ctg7180001906920_2196_SCT	9660352
25_693	ctg7180001906920_445_SGT	9662103
25_694	ctg7180001634671_5908_SCT	9693866
25_695	ctg7180001634671_3948_SCT	9695826
25_696	ctg7180001634671_3588_SAC	9696186

25_697	ctg7180001743685_1181_SAG	9709037
25_698	ctg7180001937019_2731_SGT	9735252
25_699	ctg7180001937019_11510_SAG	9744031
25_700	ctg7180001937019_12311_SAG	9744832
25_701	ctg7180001811247_9871_SAG	9758277
25_702	ctg7180001811247_9182_SAC	9758966
25_703	ctg7180001811247_9179_SCT	9758969
25_704	ctg7180001811247_2636_SGT	9765512
25_705	ctg7180001811247_2569_SAG	9765579
25_706	ctg7180001792933_11730_SCT	9771909
25_707	ctg7180001792933_11545_SCT	9772094
25_708	ctg7180001792933_11308_SCT	9772331
25_709	ctg7180001792933_9779_SAC	9773860
25_710	ctg7180001792933_9485_SAC	9774154
25_711	ctg7180001792933_5878_SCT	9777761
25_712	ctg7180001792933_4603_SAC	9779036
25_713	ctg7180001792933_3890_SAC	9779749
25_714	ctg7180001792933_2799_SGT	9780840
25_715	ctg7180001792933_2562_SAG	9781077
25_716	ctg7180001792933_1841_SCT	9781798
25_717	ctg7180001792933_1276_SAC	9782363
25_718	ctg7180001604360_498_SCT	9785791
25_719	ctg7180001604360_902_SAC	9786195
25_720	ctg7180001586346_548_SCT	9792698
25_721	ctg7180001586346_2463_SCT	9794613
25_722	ctg7180001821282_9166_SAG	9839804
25_723	ctg7180001821282_7979_SCT	9840991
25_724	ctg7180001821282_4012_SAG	9844958
25_725	ctg7180001821282_2313_SCT	9846657
25_726	ctg7180001292196_6499_SAG	9850016
25_727	ctg7180001292196_2832_SAG	9853683
25_728	ctg7180001292196_2445_SAG	9854070
25_729	ctg7180001292196_2426_SCT	9854089
25_730	ctg7180001292196_877_SCT	9855638
25_731	ctg7180001887187_3393_SCT	9868854

25_732	ctg7180001800164_1298_SCT	9877192
25_733	ctg7180001800164_1978_SAG	9877872
25_734	ctg7180001800164_5903_SAC	9881797
25_735	ctg7180001355199_160_SAC	9885587
25_736	ctg7180001937020_1779_SGT	9887916
25_737	ctg7180001937020_7703_SAC	9893840
25_738	ctg7180001937020_12198_SAG	9898335
25_739	ctg7180001937020_17924_SAC	9904061
25_740	ctg7180001937020_19833_SCT	9905970
25_741	ctg7180001937020_26723_SCT	9912860
25_742	ctg7180001937020_26809_SAC	9912946
25_743	ctg7180001937020_27007_SAG	9913144
25_744	ctg7180001937020_27378_SAC	9913515
25_745	ctg7180001837057_945_SAC	9916675
25_746	ctg7180001837057_1493_SAG	9917223
25_747	ctg7180001837057_5900_SAG	9921630
25_748	ctg7180001837057_7733_SAG	9923463
25_749	ctg7180001837057_8823_SAC	9924553
25_750	ctg7180001837057_9668_SAG	9925398
25_751	ctg7180001866525_3161_SAC	9939895
25_752	ctg7180001866525_3877_SAG	9940611
25_753	ctg7180001866525_9903_SAG	9946637
25_754	ctg7180001821901_894_SCT	9956012
25_755	ctg7180001821900_11150_SCT	9957610
25_756	ctg7180001821900_10638_SAG	9958122
25_757	ctg7180001821900_7892_SGT	9960865
25_758	ctg7180001821900_7866_SAG	9960891
25_759	ctg7180001821900_6577_SAC	9962180
25_760	ctg7180001821900_505_SAC	9968240
25_761	ctg7180001821900_11199_SGT	9968555
25_762	ctg7180001840010_4779_SCT	9971071
25_763	ctg7180001932831_18872_SCT	9976732
25_764	ctg7180001932831_1783_SAC	9993821
25_765	ctg7180001794004_2261_SAC	10006500
25_766	ctg7180001794004_1345_SAC	10007417

25_767	ctg7180001794004_780_SCT	10007982
25_768	ctg7180001860462_12668_SGT	10027014
25_769	ctg7180001860462_11167_SAG	10028515
25_770	ctg7180001860462_9372_SAG	10030310
25_771	ctg7180001860462_6435_SAG	10033247
25_772	ctg7180001860462_5886_SAG	10033796
25_773	ctg7180001860462_3111_SAC	10036571
25_774	ctg7180001860462_1996_SGT	10037686
25_775	ctg7180001860461_1158_SCT	10044618
25_776	ctg7180001874853_18955_SCT	10058946
25_777	ctg7180001874853_11695_SGT	10066206
25_778	ctg7180001874853_11647_SAG	10066254
25_779	ctg7180001874853_10774_SAC	10067127
25_780	ctg7180001874853_3350_SAC	10074551
25_781	ctg7180001874853_2320_SAG	10075581
25_782	ctg7180001455688_3628_SGT	10081940
25_783	ctg7180001455688_3048_SCT	10082520
25_784	ctg7180001902362_11293_SCT	10111095
25_785	ctg7180001902362_11351_SAC	10111153
25_786	ctg7180001897317_2347_SAT	10130608
25_787	ctg7180001897317_1350_SAG	10131605
25_788	ctg7180001813259_1157_SCT	10143258
25_789	ctg7180001813259_1090_SAG	10143325
25_790	ctg7180001923649_686_SAG	10145054
25_791	ctg7180001915130_5298_SCT	10153360
25_792	ctg7180001702532_3927_SAC	10174185
25_793	ctg7180001866298_3962_SCT	10181845
25_794	ctg7180001866298_4685_SAG	10182568
25_795	ctg7180001866298_5573_SAG	10183456
25_796	ctg7180001866298_6337_SCT	10184220
25_797	ctg7180001866298_7466_SAC	10185349
25_798	ctg7180001535605_964_SCT	10188198
25_799	ctg7180001433766_2158_SAG	10197534
25_800	ctg7180001739449_5929_SAC	10205600
25_801	ctg7180001739449_5752_SGT	10205777

25_802	ctg7180001898468_5055_SCT	10219619
25_803	ctg7180001615113_915_SAC	10236219
25_804	ctg7180001615113_1563_SAG	10236867
25_805	ctg7180001908452_3414_SAC	10242717
25_806	ctg7180001908452_5043_SAG	10244346
25_807	ctg7180001908452_6585_SAC	10245888
25_808	ctg7180001908452_6648_SAG	10245951
25_809	ctg7180001910967_4383_SCT	10255962
25_810	ctg7180001910967_10291_SAG	10261870
25_811	ctg7180001866979_2365_SGT	10266982
25_812	ctg7180001866979_2789_SCT	10267406
25_813	ctg7180001528025_7319_SGT	10299091
25_814	ctg7180001528025_4657_SAG	10301753
25_815	ctg7180001798567_1402_SAC	10312880
25_816	ctg7180001688370_3516_SAG	10333955
25_817	ctg7180001807907_1752_SAG	10356812
25_818	ctg7180001914005_3268_SCT	10373667
25_819	ctg7180001914005_3414_SAG	10373813
25_820	ctg7180001914005_3580_SAG	10373979
25_821	ctg7180001914005_5174_SGT	10375573
25_822	ctg7180001914005_5438_SAG	10375837
25_823	ctg7180001914005_5966_SCT	10376365
25_824	ctg7180001914005_7977_SCT	10378376
25_825	ctg7180001914005_8801_SAG	10379200
25_826	ctg7180001914005_10136_SAC	10380535
25_827	ctg7180001914005_10225_SCT	10380624
25_828	ctg7180001914005_17004_SCT	10387403
25_829	ctg7180001914005_17704_SCT	10388103
25_830	ctg7180001914006_7411_SCT	10405069
25_831	ctg7180001892775_534 10409495	
25_832	ctg7180001892775_1264_SAG	10419693
25_833	ctg7180001892775_706_SAG	10420251
25_834	ctg7180001795930_3295_SCT	10425800
25_835	ctg7180001795930_3353_SAG	10425858
25_836	ctg7180001795930_3487_SAC	10425992

25_837	ctg7180001795930_3586_SAG	10426091
25_838	ctg7180001818408_21706_SCT	10431401
25_839	ctg7180001818408_15605_SCT	10437502
25_840	ctg7180001818408_15436_SGT	10437671
25_841	ctg7180001818408_12383_SAC	10440724
25_842	ctg7180001818408_11799_SCT	10441308
25_843	ctg7180001912741_842_SCT	10454406
25_844	ctg7180001912741_1876_SGT	10455440
25_845	ctg7180001912741_2282_SAG	10455846
25_846	ctg7180001912741_4407_SGT	10457971
25_847	ctg7180001912741_8845_SAC	10462409
25_848	ctg7180001913733_16837_SGT	10476237
25_849	ctg7180001913733_3315_SGT	10489932
25_850	ctg7180001913733_1443_SCT	10491804
25_851	ctg7180001904937_1224_SGT	10494710
25_852	ctg7180001216944_6008_SAC	10509066
25_853	ctg7180001216944_5764_SCT	10509310
25_854	ctg7180001917939_43_SAG	10522687
25_855	ctg7180001907194_20821_SAG	10527639
25_856	ctg7180001904904_5154_SCT	10556341
25_857	ctg7180001800243_909_SAG	10588344
25_858	ctg7180001691730_929_SAC	10590426
25_859	ctg7180001845734_14563_SAG	10600683
25_860	ctg7180001845734_13562_SCT	10601684
25_861	ctg7180001845734_13472_SGT	10601774
25_862	ctg7180001845734_6341_SCT	10608905
25_863	ctg7180001845734_6290_SAC	10608956
25_864	ctg7180001845734_1777_SCT	10613469
25_865	ctg7180001845734_1337_SCT	10613909
25_866	ctg7180001802151_14276_SGT	10631268
25_867	ctg7180001802151_1818_SAG	10643726
25_868	ctg7180001802151_103_SAG	10645441
25_869	ctg7180001792132_4131_SGT	10652141
25_870	ctg7180001792132_3227_SCT	10653045
25_871	ctg7180001792132_402_SAC	10655870

25_872	ctg7180001792132_238_SAG	10656034
25_873	ctg7180001864750_1081_SAG	10657386
25_874	ctg7180001864750_3622_SCT	10660711
25_875	ctg7180001864750_5409_SGT	10662498
25_876	ctg7180001937672_18577_SCT	10667345
25_877	ctg7180001864750_18471_SAC	10675018
25_878	ctg7180001864750_19389_SAC	10675936
25_879	ctg7180001864750_20177_SAG	10676724
25_880	ctg7180001864750_22413_SAC	10678960
25_881	ctg7180001789208_692_SCT	10686055
25_882	ctg7180001789208_612_SAC	10686135
25_883	ctg7180001204739_704_SAC	10731911
25_884	ctg7180001204739_2728_SAG	10733935
25_885	ctg7180001204739_6762_SAG	10738004
25_886	ctg7180001917652_415_SAC	10740728
25_887	ctg7180001917652_595_SCT	10740908
25_888	ctg7180001917653_1307_SGT	10748381
25_889	ctg7180001917653_1352_SCT	10748426
25_890	ctg7180001917653_1624_SCT	10748698
25_891	ctg7180001637188_1206_SAG	10754968
25_892	ctg7180001637188_1534_SAC	10755296
25_893	ctg7180001829949_2018_SAG	10765958
25_894	ctg7180001281830_1837_SCT	10775777
25_895	ctg7180001389609_797_SGT	10789616
25_896	ctg7180001389609_2763_SAG	10791582
25_897	ctg7180001389609_2961_SAG	10791780
25_898	ctg7180001378642_3969_SAC	10802793
25_899	ctg7180001378642_3952_SAG	10802810
25_900	ctg7180001378642_3772_SCT	10802990
25_901	ctg7180001378642_2758_SCT	10804004
25_902	ctg7180001378642_2201_SAC	10804561
25_903	ctg7180001822507_15142_SAC	10827640
25_904	ctg7180001527144_1466_SCT	10833349
25_905	ctg7180001527144_1110_SCT	10833705
25_906	ctg7180001628409_615_SAG	10844297

25_907	ctg7180001809298_5846_SAC	10860871
25_908	ctg7180001809298_5898_SGT	10860923
25_909	ctg7180001809298_5965_SCT	10860990
25_910	ctg7180001809298_6042_SCT	10861067
25_911	ctg7180001809298_6083_SCT	10861108
25_912	ctg7180001809298_6371_SCG	10861396
25_913	ctg7180001902250_9631_SGT	10868370
25_914	ctg7180001902250_2676_SGT	10875325
25_915	ctg7180001902249_11482_SGT	10878492
25_916	ctg7180001902249_10163_SAC	10879811
25_917	ctg7180001902249_9902_SGT	10880072
25_918	ctg7180001902249_2698_SAG	10887276
25_919	ctg7180001925111_3327_SGT	10927692
25_920	ctg7180001925111_9262_SCT	10933627
25_921	ctg7180001925111_9450_SCT	10933815
25_922	ctg7180001925111_11755_SAG	10936120
25_923	ctg7180001925111_20262_SAG	10944627
25_924	ctg7180001800464_1192_SAG	10958112
25_925	ctg7180001833281_1996_SAG	10973603
25_926	ctg7180001815373_4087_SAG	10991855
25_927	ctg7180001815373_4010_SCT	10991932
25_928	ctg7180001907605_5971 11002106	
25_929	ctg7180001907605_9140_SGT	11005240
25_930	ctg7180001824858_9422_SGT	11019604
25_931	ctg7180001769198_866_SCT	11025362
25_932	ctg7180001578930_6444_SGT	11053211
25_933	ctg7180001897003_19142_SAC	11065208
25_934	ctg7180001897003_17932_SGT	11066418
25_935	ctg7180001897003_6472_SGT	11077878
25_936	ctg7180001897003_1201_SCT	11083149
25_937	ctg7180001833368_1334_SAG	11127365
25_938	ctg7180001833368_2229_SGT	11128260
25_939	ctg7180001809601_12717_SCT	11135624
25_940	ctg7180001809601_12583_SCT	11135758
25_941	ctg7180001809601_11983_SCT	11136358

25_942	ctg7180001809601_11896_SCT	11136445
25_943	ctg7180001809601_10179_SAG	11138162
25_944	ctg7180001809601_3549_SCT	11144792
25_945	ctg7180001843030_9192_SGT	11155353
25_946	ctg7180001843030_7406_SCT	11157139
25_947	ctg7180001843030_2289_SCT	11162256
25_948	ctg7180001843030_2110_SAG	11162435
25_949	ctg7180001843030_846_SGT	11163699
25_950	ctg7180001915192_10451_SAG	11173110
25_951	ctg7180001915192_6815_SCT	11176746
25_952	ctg7180001915192_4923_SGT	11178638
25_953	ctg7180001915192_4430_SGT	11179131
25_954	ctg7180001915192_4335_SAC	11179226
25_955	ctg7180001915192_4270_SAG	11179291
25_956	ctg7180001915192_4213_SAG	11179348
25_957	ctg7180001915192_824_SCT	11182737
25_958	ctg7180001846829_3696_SAG	11202688
25_959	ctg7180001846829_2446_SAC	11203938
25_960	ctg7180001846829_2420_SCT	11203964
25_961	ctg7180001846829_2279_SAC	11204105
25_962	ctg7180001358165_8306_SGT	11207365
25_963	ctg7180001358165_6351_SAC	11209320
25_964	ctg7180001358165_5229_SAG	11210442
25_965	ctg7180001358165_242_SAC	11215429
25_966	ctg7180001229997_1579_SGT	11217240
25_967	ctg7180001229997_2158_SCT	11217819
25_968	ctg7180001622829_1251_SAG	11220915
25_969	ctg7180001933552_1480_SAC	11223099
25_970	ctg7180001933552_16109_SCT	11237728
25_971	ctg7180001933552_16909_SCT	11238528
25_972	ctg7180001933552_17160_SAG	11238779
25_973	ctg7180001790927_4556_SCT	11250744
25_974	ctg7180001823613_8935_SAC	11262322
25_975	ctg7180001823613_8836_SGT	11262421
25_976	ctg7180001823613_7890_SAG	11263367

25_977	ctg7180001823613_624_SAC	11270633
25_978	ctg7180001222180_833_SAG	11277816
25_979	ctg7180001319344_2127_SCT	11284783
25_980	ctg7180001845876_1028_SAC	11287495
25_981	ctg7180001845876_4619_SCT	11291086
25_982	ctg7180001873205_296_SAG	11294225
25_983	ctg7180001315087_2655_SCT	11299598
25_984	ctg7180001869003_5556_SAG	11311304
25_985	ctg7180001869003_3757_SAG	11313103
25_986	ctg7180001869003_2926_SGT	11313934
25_987	ctg7180001721520_1185_SAG	11319717
25_988	ctg7180001927874_2570_SAG	11330730
25_989	ctg7180001439515_21605_SAG	11337892
25_990	ctg7180001439515_20883_SAG	11338614
25_991	ctg7180001439515_12019_SAG	11347478
25_992	ctg7180001439515_4458_SAC	11354940
25_993	ctg7180001439515_3842_SCT	11355556
25_994	ctg7180001439515_1178_SCT	11358220
25_995	ctg7180001439515_264_SAG	11359134
25_996	ctg7180001439515_207_SGT	11359191
25_997	ctg7180001591156_4982_SCT	11366111
25_998	ctg7180001591156_6796_SCT	11367925
25_999	ctg7180001191515_1543_SGT	11370078
25_1000	ctg7180001807531_1701_SAG	11407939
25_1001	ctg7180001807531_2982_SCT	11409220
25_1002	ctg7180001807531_3163_SCT	11409401
25_1003	ctg7180001807531_3429_SCT	11409667
25_1004	ctg7180001807531_7470_SCT	11413708
25_1005	ctg7180001807531_7479_SCT	11413717
25_1006	ctg7180001807531_14635_SCT	11420873
25_1007	ctg7180001807531_14881_SAG	11421119
25_1008	ctg7180001807531_14975_SCT	11421213
25_1009	ctg7180001807532_3718_SCT	11431126
25_1010	ctg7180001807532_3968_SAC	11431376
25_1011	ctg7180001745454_467_SGT	11467542

25_1012	ctg7180001922204_15715_SCT	11487588
25_1013	ctg7180001926398_39_SAG	11510900
25_1014	ctg7180001846909_461_SAG	11552949
25_1015	ctg7180001788319_2362_SGT	11580519
25_1016	ctg7180001901322_2945_SCT	11585609
25_1017	ctg7180001901322_725_SCT	11587829
25_1018	ctg7180001910716_7352_SAC	11592060
25_1019	ctg7180001910716_5402_SAG	11594010
25_1020	ctg7180001910715_16718_SGT	11600549
25_1021	ctg7180001910715_15306_SCT	11601961
25_1022	ctg7180001910715_5801_SCT	11611466
25_1023	ctg7180001528210_924_SCT	11625113
25_1024	ctg7180001865524_8446_SGT	11634722
25_1025	ctg7180001897741_3987_SCT	11650564
25_1026	ctg7180001897742_428_SAC	11652693
25_1027	ctg7180001806488_2966_SGT	11679547
25_1028	ctg7180001588397_301_SAG	11688559
25_1029	ctg7180001865754_4091_SAC	11700518
25_1030	ctg7180001865754_1967_SAC	11702642
25_1031	ctg7180001547550_944_SAC	11718209
25_1032	ctg7180001481239_1283_SCT	11740281
25_1033	ctg7180001799661_21572_SAG	11745146
25_1034	ctg7180001799661_10947_SCT	11755470
25_1035	ctg7180001799661_10283_SAC	11756134
25_1036	ctg7180001799661_8810_SCT	11757607
25_1037	ctg7180001799661_6489_SAG	11759928
25_1038	ctg7180001799661_3937_SGT	11762480
25_1039	ctg7180001799661_1105_SCT	11765312
25_1040	ctg7180001440983_2817_SAG	11789726
25_1041	ctg7180001485051_124_SCT	11793853
25_1042	ctg7180001820563_559_SCT	11795334
25_1043	ctg7180001820563_1682_SGT	11796458
25_1044	ctg7180001820563_2264_SAC	11797040
25_1045	ctg7180001820563_3008_SAC	11797784
25_1046	ctg7180001820563_3610_SGT	11798387

25_1047	ctg7180001795447_8592_SCT	11810311
25_1048	ctg7180001795447_6145_SAG	11812758
25_1049	ctg7180001795447_4387_SAG	11814516
25_1050	ctg7180001795447_365_SCT	11818538
25_1051	ctg7180001820170_11272_SAG	11819760
25_1052	ctg7180001820170_9758_SCT	11821274
25_1053	ctg7180001820170_9409_SAG	11821623
25_1054	ctg7180001820170_2487_SAG	11828546
25_1055	ctg7180001820170_1654_SGT	11829379
25_1056	ctg7180001227682_1526_SAG	11831322
25_1057	ctg7180001227682_378_SAC	11832470
25_1058	ctg7180001871954_17323_SAG	11834019
25_1059	ctg7180001871954_6164_SCT	11845179
25_1060	ctg7180001871954_5714_SAG	11845630
25_1061	ctg7180001871954_3670_SAC	11847674
25_1062	ctg7180001871954_1762_SGT	11849582
25_1063	ctg7180001862185_8175_SCT	11853787
25_1064	ctg7180001547439_2058_SAG	11876454
25_1065	ctg7180001547439_1609_SCT	11876903
25_1066	ctg7180001547439_1500_SGT	11877012
25_1067	ctg7180001547439_1829_SCT	11877103
25_1068	ctg7180001823978_10358_SCT	11896730
25_1069	ctg7180001929502_7605_SCT	11914750
25_1070	ctg7180001328908_2960_SGT	11919967
25_1071	ctg7180001328908_3933_SCT	11920940
25_1072	ctg7180001328908_5052_SCT	11922059
25_1073	ctg7180001214573_2288_SGT	11938399
25_1074	ctg7180001214573_5272_SAG	11941383
25_1075	ctg7180001214573_6155_SGT	11942266
25_1076	ctg7180001214573_7109_SCT	11943220
25_1077	ctg7180001794793_5075_SCT	11949140
25_1078	ctg7180001794793_6425_SCT	11950490
25_1079	ctg7180001794793_9511_SAG	11953576
25_1080	ctg7180001794793_10610_SAG	11954675
25_1081	ctg7180001811672_1661_SAG	11964530

25_1082	ctg7180001855733_5784_SAC	11973630
25_1083	ctg7180001935082_3692_SAG	11981802
25_1084	ctg7180001935082_7981_SCT	11986054
25_1085	ctg7180001935082_10080_SCT	11988153
25_1086	ctg7180001935082_15389_SAC	11993462
25_1087	ctg7180001286913_1026_SCT	12025503
25_1088	ctg7180001897056_4029_SGT	12035033
25_1089	ctg7180001897056_5810_SAG	12036814
25_1090	ctg7180001897056_7687_SCT	12038691
25_1091	ctg7180001257048_6191_SCT	12044962
25_1092	ctg7180001257048_6082_SCT	12045071
25_1093	ctg7180001257048_4154_SAC	12046999
25_1094	ctg7180001843451_5478_SCT	12053519
25_1095	ctg7180001444165_1203_SCT	12066386
25_1096	ctg7180001444165_1480_SCT	12066663
25_1097	ctg7180001769476_378_SAC	12077890
25_1098	ctg7180001724871_2064_SCT	12080113
25_1099	ctg7180001261775_817_SAG	12081023
25_1100	ctg7180001845383_1884_SGT	12089681
25_1101	ctg7180001869076_1835_SAC	12136851
25_1102	ctg7180001869076_2370_SCT	12137386
25_1103	ctg7180001919341_32513_SCT	12152032
25_1104	ctg7180001919341_31608_SAG	12152937
25_1105	ctg7180001919341_8456_SAC	12176090
25_1106	ctg7180001919341_7107_SGT	12177439
25_1107	ctg7180001463897_725_SGT	12231667
25_1108	ctg7180001878177_7409_SCT	12239622
25_1109	ctg7180001906643_5292_SAC	12248076
25_1110	ctg7180001906643_13046_SAG	12250395
25_1111	ctg7180001906643_6123_SAG	12257355
25_1112	ctg7180001635049_1619_SAG	12265770
25_1113	ctg7180001298596_1178_SAG	12267948
25_1114	ctg7180001759824_752_SCT	12269856
25_1115	ctg7180001848910_15231_SAG	12291216
25_1116	ctg7180001848910_15111_SAG	12291336

25_1117	ctg7180001810589_270_SCT	12316292
25_1118	ctg7180001810589_160_SGT	12316402
25_1119	ctg7180001796314_7416_SAC	12317138
25_1120	ctg7180001796314_7302_SAG	12317252
25_1121	ctg7180001796314_2991_SAG	12321563
25_1122	ctg7180001796314_1511_SAG	12323043
25_1123	ctg7180001796314_190_SCT	12324364
25_1124	ctg7180001898022_8512_SGT	12356403
25_1125	ctg7180001898022_3685_SCT	12361230
25_1126	ctg7180001898022_2477_SCT	12362436
25_1127	ctg7180001537452_1194_SAC	12368566
25_1128	ctg7180001841174_18123_SAG	12375940
25_1129	ctg7180001841174_17265_SCT	12376798
25_1130	ctg7180001841174_10886_SAG	12383177
25_1131	ctg7180001841174_6954_SAG	12387109
25_1132	ctg7180001841174_6212_SCT	12387851
25_1133	ctg7180001841174_5170_SCT	12388893
25_1134	ctg7180001841174_3092_SCT	12390971
25_1135	ctg7180001841174_1737_SGT	12392326
25_1136	ctg7180001186552_3036_SGT	12397948
25_1137	ctg7180001186552_3522_SCT	12398434
25_1138	GCR_cBin466_Ctg1_234	12401841
25_1139	ctg7180001799478_3541_SCT	12411112
25_1140	ctg7180001799478_4112_SAC	12411683
25_1141	ctg7180001799478_6633_SAC	12414239
25_1142	ctg7180001799478_7797_SAC	12415368
25_1143	ctg7180001868148_1791_SGT	12439072
25_1144	ctg7180001868148_1709_SGT	12439154
25_1145	ctg7180001871711_3633_SAG	12449338
25_1146	ctg7180001871711_2860_SCT	12450111
25_1147	ctg7180001823124_4376_SGT	12460387
25_1148	ctg7180001427879_1991_SAG	12466769
25_1149	ctg7180001427879_3494_SAG	12468272
25_1150	ctg7180001389052_493_SAG	12469011
25_1151	ctg7180001389052_460_SAG	12469013

25_1152	ctg7180001867332_12274_SCT	12487298
25_1153	ctg7180001935286_1420_SAG	12491472
25_1154	ctg7180001935286_2450_SAG	12492502
25_1155	ctg7180001935286_5199_SCT	12495251
25_1156	ctg7180001935286_5779_SAG	12495831
25_1157	ctg7180001935286_13152_SAC	12503204
25_1158	ctg7180001935286_14962_SAC	12505014
25_1159	ctg7180001935286_19612_SAG	12509663
25_1160	ctg7180001935286_20656_SCT	12510707
25_1161	ctg7180001805887_2454_SGT	12534835
25_1162	ctg7180001245633_2167_SAG	12538715
25_1163	ctg7180001855710_4907_SCT	12550127
25_1164	ctg7180001295818_3067_SCT	12558568
25_1165	ctg7180001278497_484_SAG	12578782
25_1166	ctg7180001278497_4791_SCT	12583089
25_1167	ctg7180001743547_1030_SCT	12590642
25_1168	ctg7180001901227_13900_SCT	12594988
25_1169	ctg7180001901227_12880_SCT	12596008
25_1170	ctg7180001901227_12067_SAG	12596821
25_1171	ctg7180001901227_11435_SCT	12597453
25_1172	ctg7180001901227_10234_SGT	12598654
25_1173	ctg7180001901227_9769_SCT	12599119
25_1174	ctg7180001901227_9212_SAC	12599676
25_1175	ctg7180001901227_8132_SAG	12600756
25_1176	ctg7180001901227_6815_SCT	12602073
25_1177	ctg7180001722864_7346_SAG	12620284
25_1178	ctg7180001722864_11340_SCT	12624278
25_1179	ctg7180001689951_3662_SAG	12638521
25_1180	ctg7180001689951_1558_SGT	12640625
25_1181	ctg7180001859764_6227_SAC	12644305
25_1182	ctg7180001868286_12431_SCT	12691440
25_1183	ctg7180001868286_11143_SCT	12692724
25_1184	ctg7180001868286_9995_SGT	12693872
25_1185	ctg7180001868286_6840_SAC	12697027
25_1186	ctg7180001868286_6785_SAC	12697082

25_1187	ctg7180001868286_5186_SCT	12698681
25_1188	ctg7180001868286_4998_SAC	12698869
25_1189	ctg7180001868286_2379_SCT	12701488
25_1190	ctg7180001868286_2346_SGT	12701521
25_1191	ctg7180001868286_1542_SCT	12702325
25_1192	ctg7180001304401_6202_SAC	12713225
25_1193	ctg7180001856795_13840_SAG	12733191
25_1194	ctg7180001934933_19690_SCT	12754330
25_1195	ctg7180001934933_25668_SCT	12760308
25_1196	GCR_cBin22511_Ctg1_246	12767399
25_1197	ctg7180001840776_28488_SAC	12794095
25_1198	ctg7180001840776_7313	12799347
25_1199	ctg7180001840776_13135_SAG	12809447
25_1200	ctg7180001840776_13049_SGT	12809533
25_1201	ctg7180001840776_12963_SAG	12809619
25_1202	ctg7180001898541_423_SCT	12819664
25_1203	ctg7180001803858_13333_SAC	12837802
25_1204	ctg7180001803858_12519_SAG	12838616
25_1205	ctg7180001803858_7544_SAC	12843698
25_1206	ctg7180001803858_6000_SGT	12845242
25_1207	ctg7180001799730_5571_SAG	12859042
25_1208	ctg7180001799730_1484_SCT	12863129
25_1209	ctg7180001799730_1261_SAG	12863352
25_1210	ctg7180001697830_16682_SGT	12867724
25_1211	ctg7180001697830_11182_SAG	12873224
25_1212	ctg7180001697830_10377_SCT	12874029
25_1213	ctg7180001697830_5681_SAG	12878725
25_1214	ctg7180001697830_5062_SAC	12879344
25_1215	ctg7180001697830_4935_SAC	12879471
25_1216	ctg7180001697830_2048_SAG	12882358
25_1217	ctg7180001582895_939_SCT	12918677
25_1218	ctg7180001860240_12997_SCT	12919875
25_1219	ctg7180001860240_9887_SAG	12929553
25_1220	ctg7180001903656_9829_SAC	12957487
25_1221	ctg7180001903657_2442_SGT	12962156

25_1222	ctg7180001903657_5895_SAG	12965609
25_1223	ctg7180001903657_6304_SGT	12966018
25_1224	ctg7180001434805_1740_SAC	12974940
25_1225	ctg7180001434805_1895_SAC	12975095
25_1226	ctg7180001434805_2450_SAC	12975650
25_1227	ctg7180001496951_2109_SAG	12978009
25_1228	ctg7180001496951_1822_SCT	12978296
25_1229	ctg7180001496951_1347_SCT	12978771
25_1230	ctg7180001496951_1242_SGT	12978876
25_1231	ctg7180001804742_102_SAG	12983689
25_1232	ctg7180001753374_2035_SAG	13003407
25_1233	ctg7180001753374_1341_SAG	13004101
25_1234	ctg7180001211403_2738_SGT	13009931
25_1235	ctg7180001792191_4499_SGT	13020123
25_1236	ctg7180001792193_2027_SGT	13024326
25_1237	ctg7180001536638_2516_SAG	13036839
25_1238	ctg7180001826606_934_SAG	13045629
25_1239	ctg7180001826606_6958_SAG	13051653
25_1240	ctg7180001901329_10935_SCT	13060767
25_1241	ctg7180001901329_6971_SCT	13064731
25_1242	ctg7180001901329_3668_SCT	13068034
25_1243	ctg7180001901329_10285_SGT	13068051
25_1244	ctg7180001901329_1935_SGT	13069767
25_1245	ctg7180001901329_718_SGT	13070984
25_1246	ctg7180001896175_8313_SAG	13074609
25_1247	ctg7180001792505_789_SAG	13099204
25_1248	ctg7180001792505_2729_SGT	13101144
25_1249	ctg7180001792505_5143_SAG	13103558
25_1250	ctg7180001713094_1429_SAG	13106355
25_1251	ctg7180001806132_1760_SCT	13115740
25_1252	ctg7180001802522_1424_SCT	13128490
25_1253	ctg7180001619100_154_SCT	13130844
25_1254	ctg7180001542949_1393_SAG	13136893
25_1255	ctg7180001649349_864_SCT	13141891
25_1256	ctg7180001263016_12740_SAG	13143517

25_1257	GCR_cBin2569_Ctg1_435	13143765
25_1258	ctg7180001263016_10030_SGT	13146227
25_1259	ctg7180001263016_9871_SGT	13146386
25_1260	ctg7180001263016_9845_SAG	13146412
25_1261	ctg7180001263016_4678_SCT	13151579
25_1262	ctg7180001263016_3931_SAG	13152326
25_1263	ctg7180001858986_16957_SAC	13186088
25_1264	ctg7180001858986_19333_SAG	13188464
25_1265	ctg7180001858986_20425_SAG	13189556
25_1266	ctg7180001858986_20786_SGT	13189917
25_1267	ctg7180001446392_2935_SAG	13191013
25_1268	ctg7180001446392_2784_SAG	13191164
25_1269	ctg7180001446392_2456_SCT	13191492
25_1270	ctg7180001888625_3156_SCT	13220017
25_1271	ctg7180001589825_3146_SGT	13244480
25_1272	ctg7180001265023_3622_SAG	13260097
25_1273	ctg7180001232768_231_SAC	13305283
25_1274	ctg7180001798739_9523_SAC	13309751
25_1275	ctg7180001798739_7975_SAG	13311299
25_1276	ctg7180001798739_3562_SGT	13315712
25_1277	ctg7180001841187_5839_SGT	13362340
25_1278	ctg7180001518741_4972_SAC	13398749
25_1279	ctg7180001865598_2675_SCT	13417448
25_1280	ctg7180001809046_3857_SCT	13433669
25_1281	ctg7180001834645_3184_SAC	13442094
25_1282	ctg7180001834645_3750_SCT	13442660
25_1283	ctg7180001572527_6494_SGT	13444869
25_1284	ctg7180001820751_8382_SAC	13457641
25_1285	ctg7180001820751_7720_SCT	13458303
25_1286	ctg7180001820751_7272_SGT	13458751
25_1287	ctg7180001797138_226_SAC	13478492
25_1288	ctg7180001261473_1020_SCT	13480656
25_1289	ctg7180001261473_1441_SAG	13481077
25_1290	ctg7180001860727_15859_SCT	13507905
25_1291	ctg7180001860727_31430_SCT	13523476

25_1292	ctg7180001665834_5692_SCT	13528299
25_1293	ctg7180001665834_4284_SCT	13529707
25_1294	ctg7180001665834_4264_SGT	13529727
25_1295	ctg7180001665834_3297_SAC	13530694
25_1296	ctg7180001797947_9549_SAC	13538913
25_1297	ctg7180001797947_9197_SGT	13539265
25_1298	ctg7180001797947_7463_SAC	13540999
25_1299	ctg7180001797947_5432_SAG	13543030
25_1300	ctg7180001908211_685_SCT	13550321
25_1301	ctg7180001908211_5190_SCT	13554826
25_1302	ctg7180001833406_5576_SAG	13581541
25_1303	ctg7180001794762_10902_SGT	13585515
25_1304	ctg7180001794762_6938_SAG	13589479
25_1305	ctg7180001794762_6136_SCT	13590281
25_1306	ctg7180001855493_66_SCT	13595257
25_1307	ctg7180001649109_4288_SCT	13620593
25_1308	ctg7180001811620_4944_SCT	13648945
25_1309	ctg7180001201753_1153_SCT	13652289
25_1310	ctg7180001254686_5509_SAC	13658726
25_1311	ctg7180001872473_8796_SAC	13667530
25_1312	ctg7180001872473_8763_SAG	13667563
25_1313	ctg7180001872473_4211_SGT	13672115
25_1314	ctg7180001872473_4012_SAG	13672314
25_1315	ctg7180001887307_18011_SGT	13678549
25_1316	ctg7180001887307_10952_SAG	13685608
25_1317	ctg7180001924157_7978_SAG	13702525
25_1318	ctg7180001924157_7538_SAG	13702965
25_1319	ctg7180001924157_7342_SGT	13703161
25_1320	ctg7180001805381_6456_SGT	13718295
25_1321	ctg7180001805381_6092_SGT	13718659
25_1322	ctg7180001877196_2068_SAG	13726588
25_1323	ctg7180001805380_3722_SAC	13729298
25_1324	ctg7180001805380_496_SAC	13732524
25_1325	ctg7180001477774_310_SAG	13753837
25_1326	ctg7180001809818_1896_SAC	13759922

25_1327	ctg7180001809818_1996_SAG	13760022
25_1328	ctg7180001937397_11561_SCT	13772693
25_1329	ctg7180001937397_13409_SAC	13774541
25_1330	ctg7180001937397_15034_SAC	13776166
25_1331	ctg7180001937397_15761_SAC	13776893
25_1332	ctg7180001937397_16538_SGT	13777670
25_1333	ctg7180001937397_16630_SAG	13777762
25_1334	ctg7180001791023_1257	13780820
25_1335	ctg7180001791023_11251_SCT	13781119
25_1336	ctg7180001791023_11230_SGT	13781140
25_1337	ctg7180001791023_10847_SAG	13781523
25_1338	ctg7180001791023_10732_SCT	13781638
25_1339	ctg7180001791023_10600_SAG	13781770
25_1340	ctg7180001791023_8936_SAT	13783434
25_1341	ctg7180001791023_6123_SCT	13786247
25_1342	ctg7180001431565_1578_SAC	13806121
25_1343	ctg7180001431565_2129_SCT	13806672
25_1344	ctg7180001431565_8895_SGT	13813438
25_1345	ctg7180001443722_2913_SCT	13817852
25_1346	ctg7180001443722_2906_SCT	13817859
25_1347	ctg7180001443722_2837_SGT	13817928
25_1348	ctg7180001443722_2013_SCT	13818752
25_1349	ctg7180001443722_1269_SAG	13819496
25_1350	ctg7180001932956_824_SAG	13822345
25_1351	ctg7180001932956_3120_SCT	13824642
25_1352	ctg7180001932956_5658_SAC	13827180
25_1353	ctg7180001932956_7098_SAG	13828620
25_1354	ctg7180001932956_9044_SAG	13830566
25_1355	ctg7180001814724_10561_SAG	13845430
25_1356	ctg7180001814724_1273_SAC	13854718
25_1357	ctg7180001877552_4408_SAC	13861171
25_1358	ctg7180001877552_643_SCT	13864936
25_1359	ctg7180001487314_525_SCT	13868457
25_1360	ctg7180001825820_16168_SCT	13871806
25_1361	ctg7180001825820_15528_SAG	13872446

25_1362	ctg7180001825820_15421_SCT	13872553
25_1363	ctg7180001825820_12090_SCT	13875884
25_1364	ctg7180001825820_9259_SCT	13878715
25_1365	ctg7180001328260_445_SCT	13897633
25_1366	ctg7180001795021_17049_SGT	13905640
25_1367	ctg7180001795021_7398_SGT	13915290
25_1368	ctg7180001570302_1764_SAG	13925199
25_1369	ctg7180001570302_750_SGT	13926213
25_1370	ctg7180001806439_925_SAG	13931313
25_1371	ctg7180001694933_5013_SAC	13966522
25_1372	ctg7180001694933_6447_SCT	13967956
25_1373	ctg7180001694933_7449_SCT	13968958
25_1374	ctg7180001694933_8147_SCT	13969656
25_1375	ctg7180001694933_10680_SAG	13972189
25_1376	ctg7180001694933_11014_SAG	13972523
25_1377	ctg7180001694933_12988_SCT	13974497
25_1378	ctg7180001694933_15820_SAG	13977329
25_1379	ctg7180001694933_19200_SAC	13980709
25_1380	ctg7180001694933_28626_SCT	13990135
25_1381	ctg7180001823145_1985_SGT	13996877
25_1382	ctg7180001823145_7661_SAC	14002553
25_1383	ctg7180001823145_9501_SAG	14004393
25_1384	ctg7180001823145_12406_SCT	14007298
25_1385	ctg7180001792802_13916 14025089	
25_1386	ctg7180001714005_204_SGT	14027529
25_1387	ctg7180001714005_266_SAC	14027591
25_1388	ctg7180001714005_1458_SCT	14028783
25_1389	ctg7180001298896_1472_SAC	14031696
25_1390	ctg7180001529746_1216 14035047	
25_1391	ctg7180001529746_907_SGT	14035053
25_1392	ctg7180001195351_673_SAG	14036683
25_1393	ctg7180001195351_6392_SAG	14042402
25_1394	ctg7180001195351_6594_SCT	14042604
25_1395	ctg7180001930404_25919_SCT	14050541
25_1396	ctg7180001787827_17259_SAG	14092408

25_1397	ctg7180001787827_16403_SCT	14093264
25_1398	ctg7180001787827_16077_SAG	14093590
25_1399	ctg7180001805565_3401_SAC	14111357
25_1400	ctg7180001805565_1525_SAC	14113233
25_1401	ctg7180001805565_942_SAG	14113816
25_1402	ctg7180001242993_1050_SCT	14152051
25_1403	ctg7180001242993_857_SCT	14152244
25_1404	ctg7180001892895_3631_SCT	14158418
25_1405	ctg7180001397418_1388_SAG	14167969
25_1406	ctg7180001868335_1848_SAG	14171268
25_1407	ctg7180001868335_2121_SCT	14171541
25_1408	ctg7180001868335_2209_SAC	14171629
25_1409	ctg7180001868335_9500_SCT	14178920
25_1410	ctg7180001868335_9837_SCT	14179257
25_1411	ctg7180001868335_10178_SAG	14179598
25_1412	ctg7180001868335_10524_SAG	14179944
25_1413	ctg7180001868335_11904_SAG	14181324
25_1414	ctg7180001826649_1478_SAC	14200664
25_1415	ctg7180001826649_1418_SCT	14200724
25_1416	ctg7180001917291_5198_SAG	14243739
25_1417	ctg7180001917291_16032_SAG	14254574
25_1418	ctg7180001917291_18830_SAG	14257372
25_1419	ctg7180001807671_693_SAG	14258958
25_1420	ctg7180001807671_1874_SAG	14260139
25_1421	ctg7180001807671_7572_SGT	14265803
25_1422	ctg7180001548826_3464_SCT	14267138
25_1423	ctg7180001548826_3105_SCT	14267497
25_1424	ctg7180001548826_1194_SAG	14269408
25_1425	ctg7180001548826_693_SCT	14269909
25_1426	ctg7180001880481_34787_SCT	14278875
25_1427	ctg7180001880481_34760_SAG	14278902
25_1428	ctg7180001880481_21958_SGT	14291704
25_1429	ctg7180001880481_18421_SAG	14295241
25_1430	ctg7180001880481_18244_SCT	14295418
25_1431	ctg7180001880481_18086_SCT	14295576

25_1432	ctg7180001880481_7342_SGT	14306321
25_1433	ctg7180001880481_6393_SAC	14307270
25_1434	ctg7180001880481_6243_SAC	14307420
25_1435	ctg7180001880481_6232_SAC	14307431
25_1436	ctg7180001880481_3873_SCT	14309790
25_1437	ctg7180001880481_2786_SCT	14310877
25_1438	ctg7180001880481_2781_SCT	14310882
25_1439	ctg7180001819624_5971_SAG	14326274
25_1440	ctg7180001819624_5573_SCT	14326672
25_1441	ctg7180001819624_2152_SAG	14330093
25_1442	ctg7180001819624_1104_SCT	14331141
25_1443	ctg7180001195769_2601_SGT	14333803
25_1444	ctg7180001770212_2742_SCT	14341076
25_1445	ctg7180001776038_1765_SCT	14344406
25_1446	ctg7180001776038_1402_SGT	14344769
25_1447	ctg7180001855381_38132_SAC	14353281
25_1448	ctg7180001855381_36232_SAG	14355181
25_1449	ctg7180001855381_36139_SAC	14355274
25_1450	ctg7180001855381_36096_SAG	14355317
25_1451	ctg7180001855381_36093_SAC	14355320
25_1452	ctg7180001855381_31537_SCT	14359876
25_1453	ctg7180001855381_24129_SAG	14367284
25_1454	ctg7180001855381_9276_SAG	14382137
25_1455	ctg7180001888903_8122_SCT	14394754
25_1456	ctg7180001888903_9118_SAC	14395750
25_1457	ctg7180001888903_17584_SCT	14404216
25_1458	ctg7180001904716_6572_SAG	14411312
25_1459	ctg7180001904716_7446_SCT	14412186
25_1460	ctg7180001724900_440_SCT	14413221
25_1461	ctg7180001793293_1488_SAG	14428367
25_1462	ctg7180001793293_2579_SAG	14429458
25_1463	ctg7180001793293_3251_SAC	14430130
25_1464	ctg7180001793294_570_SAC	14438365
25_1465	ctg7180001793294_3286_SAG	14441081
25_1466	ctg7180001661712_3538_SAC	14445124

25_1467	ctg7180001661712_2861_SAC	14445801
25_1468	ctg7180001661712_2694_SAG	14445968
25_1469	ctg7180001818391_6178_SCT	14454949
25_1470	ctg7180001818391_7222_SCT	14455993
25_1471	ctg7180001818391_7757_SAG	14456528
25_1472	ctg7180001818391_8634_SAG	14457405
25_1473	ctg7180001818391_9652_SAG	14458423
25_1474	ctg7180001818391_12059_SCT	14460830
25_1475	ctg7180001818391_14473_SCT	14463244
25_1476	ctg7180001818391_14896_SGT	14463667
25_1477	ctg7180001457626_5992_SAG	14467128
25_1478	ctg7180001804964_1954_SAG	14501145
25_1479	ctg7180001804964_1138_SCT	14501961
25_1480	ctg7180001925474_4177_SCT	14511672
25_1481	ctg7180001925474_17143_SAC	14524638
25_1482	ctg7180001925474_18880_SCT	14526375
25_1483	ctg7180001543793_1174_SGT	14552615
25_1484	ctg7180001884124_4925_SGT	14560079
25_1485	ctg7180001884124_5005_SCT	14560159
25_1486	ctg7180001884124_5081_SCT	14560235
25_1487	ctg7180001891857_605_SCT	14566987
25_1488	ctg7180001891857_2108_SAG	14568490
25_1489	ctg7180001891857_2265_SAC	14568647
25_1490	ctg7180001891857_2595_SCT	14568977
25_1491	ctg7180001891857_2949_SCT	14569331
25_1492	ctg7180001891857_7310_SCT	14573692
25_1493	ctg7180001891857_7776_SAG	14574158
25_1494	ctg7180001719494_494_SAG	14596021
25_1495	ctg7180001822187_1719_SCT	14600713
25_1496	ctg7180001822187_908_SCT	14601524
25_1497	ctg7180001822186_5583_SGT	14613111
25_1498	ctg7180001822186_3638_SGT	14615056
25_1499	ctg7180001822186_1791_SCT	14616904
25_1500	ctg7180001822186_1065_SAG	14617630
25_1501	ctg7180001822186_76_SAG	14618619

25_1502	ctg7180001818388_3386_SAG	14672457
25_1503	ctg7180001818388_7363_SAG	14676435
25_1504	ctg7180001818388_10162_SAG	14679234
25_1505	ctg7180001809312_983_SAC	14682965
25_1506	ctg7180001809312_4536_SAG	14686518
25_1507	ctg7180001809312_5461_SCT	14687443
25_1508	ctg7180001809312_5966_SAG	14687948
25_1509	ctg7180001661845_946_SGT	14695911
25_1510	ctg7180001937649_6431_SAG	14698871
25_1511	ctg7180001937649_3527_SGT	14701775
25_1512	ctg7180001937648_13682_SGT	14709600
25_1513	ctg7180001937648_11348_SAC	14711934
25_1514	ctg7180001937648_7261_SCT	14716021
25_1515	ctg7180001937648_4185_SCT	14719097
25_1516	ctg7180001589914_881_SGT	14724588
25_1517	ctg7180001589914_1767_SCT	14725474
25_1518	ctg7180001589914_7155_SCT	14730862
25_1519	ctg7180001589914_7369_SGT	14731076
25_1520	ctg7180001589914_7612_SAG	14731319
25_1521	ctg7180001589914_7943_SCT	14731650
25_1522	ctg7180001589914_10900_SAG	14734607
25_1523	ctg7180001589914_19264_SAC	14742971
25_1524	ctg7180001589914_19366_SAG	14743073
25_1525	ctg7180001589914_19401_SAC	14743108
25_1526	ctg7180001589914_20429_SGT	14744136
25_1527	ctg7180001589914_21166_SAC	14744873
25_1528	ctg7180001587754_685_SCT	14748515
25_1529	ctg7180001587754_2493_SGT	14750323
25_1530	ctg7180001587754_6795_SCT	14753704
25_1531	ctg7180001821668_12096_SCT	14770007
25_1532	ctg7180001799783_2319_SAG	14783234
25_1533	ctg7180001343691_1183_SAG	14793778
25_1534	ctg7180001860012_5933_SAC	14825489
25_1535	ctg7180001860012_6850_SAG	14826406
25_1536	ctg7180001860012_6885_SCT	14826441

25_1537	ctg7180001853008_1263_SCT	14848280
25_1538	ctg7180001853008_1043_SAG	14848500
25_1539	ctg7180001274718_8744_SAG	14851286
25_1540	ctg7180001274718_8256_SGT	14851774
25_1541	ctg7180001274718_6239_SAG	14853791
25_1542	ctg7180001274718_5300_SGT	14854730
25_1543	ctg7180001274718_3450_SCT	14856580
25_1544	ctg7180001205881_1179_SGT	14863526
25_1545	ctg7180001362795_4945_SAG	14870295
25_1546	ctg7180001362795_4999_SCT	14870349
25_1547	ctg7180001362795_5107_SAG	14870457
25_1548	ctg7180001362795_5170_SAC	14870520
25_1549	ctg7180001362795_6878_SGT	14872228
25_1550	ctg7180001232337_4168_SAG	14882535
25_1551	ctg7180001232337_5435_SAC	14883802
25_1552	ctg7180001232337_5805_SAC	14884172
25_1553	ctg7180001847184_402_SAG	14885420
25_1554	ctg7180001847184_1369_SGT	14886387
25_1555	ctg7180001847184_12417_SCT	14897435
25_1556	ctg7180001901926_5696_SAG	14903555
25_1557	ctg7180001901926_2224_SAG	14907027
25_1558	ctg7180001901926_975_SAG	14908276
25_1559	ctg7180001487801_11759_SCT	14922174
25_1560	ctg7180001487801_13224_SAG	14923639
25_1561	ctg7180001487801_13602_SGT	14924017
25_1562	ctg7180001487801_13672_SAG	14924087
25_1563	ctg7180001487801_16428_SCT	14926843
25_1564	ctg7180001520904_3358_SAC	14945288
25_1565	ctg7180001789174_5389_SAG	14954183
25_1566	ctg7180001789174_3256_SAG	14956316
25_1567	ctg7180001789173_3447_SCT	14960930
25_1568	ctg7180001789173_3025_SAC	14961352
25_1569	ctg7180001334937_243_SAG	14965307
25_1570	ctg7180001817994_1705_SAG	14973347
25_1571	ctg7180001817994_8836_SAG	14980478

25_1572	ctg7180001671425_4974_SGT	14986242
25_1573	ctg7180001671425_4657_SAG	14986559
25_1574	ctg7180001879591_6125_SGT	14998258
25_1575	ctg7180001307516_952_SAG	15021446
25_1576	ctg7180001598663_760_SAG	15031998
25_1577	ctg7180001598663_2533_SCT	15033771
25_1578	ctg7180001598663_4830_SCT	15036068
25_1579	ctg7180001598663_6250_SCT	15037488
25_1580	ctg7180001598663_7914_SAC	15039152
25_1581	ctg7180001598663_8638_SGT	15039876
25_1582	ctg7180001597210_82_SAC	15047174
25_1583	ctg7180001498987_1929_SGT	15048591
25_1584	ctg7180001498987_1780_SCT	15048740
25_1585	ctg7180001498987_976_SAG	15049544
25_1586	ctg7180001572635_5931_SAG	15056436
25_1587	ctg7180001572635_4973_SAG	15057394
25_1588	ctg7180001572635_1472_SAG	15060895
25_1589	ctg7180001572635_950_SAC	15061417
25_1590	ctg7180001430603_3625_SAC	15064352
25_1591	ctg7180001808570_295_SAC	15073544
25_1592	ctg7180001808570_4451_SGT	15077700
25_1593	ctg7180001808570_5691_SAG	15078940
25_1594	ctg7180001808570_8393_SCT	15081642
25_1595	ctg7180001793417_1928_SGT	15097311
25_1596	ctg7180001808529_2099_SCT	15103520
25_1597	ctg7180001710793_675_SGT	15117137
25_1598	ctg7180001710793_248_SAG	15117564
25_1599	ctg7180001457880_1068_SAG	15122118
25_1600	ctg7180001196945_3367_SAC	15124377
25_1601	ctg7180001196945_3108_SAC	15124636
25_1602	ctg7180001590711_923_SAG	15128762
25_1603	ctg7180001590711_1252_SAG	15129091
25_1604	ctg7180001865752_2465_SAG	15157688
25_1605	ctg7180001865752_2079_SGT	15158074
25_1606	ctg7180001865752_1935_SAG	15158218

25_1607	ctg7180001865752_1778_SGT	15158375
25_1608	ctg7180001622509_2212_SAG	15162982
25_1609	ctg7180001622509_2237_SCT	15163007
25_1610	ctg7180001622509_3002_SAG	15163772
25_1611	ctg7180001688202_2571_SCT	15174043
25_1612	ctg7180001688202_4634_SCT	15176105
25_1613	ctg7180001688202_7857_SGT	15179328
25_1614	ctg7180001688202_8101_SCT	15179572
25_1615	ctg7180001906631_1271_SAG	15188233
25_1616	ctg7180001906631_4434_SAG	15191396
25_1617	ctg7180001873402_338_SAC	15202189
25_1618	ctg7180001814621_3606_SAG	15206189
25_1619	ctg7180001814621_7767_SCT	15210350
25_1620	ctg7180001814621_9502_SAG	15212085
25_1621	ctg7180001814621_13016_SAC	15215599
25_1622	ctg7180001814621_14952	15217570
25_1623	ctg7180001814621_16194_SGT	15218777
25_1624	ctg7180001814621_16210_SGT	15218793
25_1625	ctg7180001814621_22813_SAG	15225396
25_1626	ctg7180001814621_27754_SAG	15230337
25_1627	ctg7180001814621_31016_SAC	15233599
25_1628	ctg7180001533131_7897_SAG	15244904
25_1629	ctg7180001533131_6069_SCT	15246732
25_1630	ctg7180001533131_5985_SAG	15246816
25_1631	ctg7180001533131_5977_SCT	15246824
25_1632	ctg7180001533131_2056_SGT	15250745
25_1633	ctg7180001876535_15250_SAG	15272127
25_1634	ctg7180001876535_15078_SAG	15272299
25_1635	ctg7180001652022_2655_SCT	15298940
25_1636	ctg7180001556714_1294_SAG	15303206
25_1637	ctg7180001738556_870_SAC	15319999
25_1638	ctg7180001797261_649_SAG	15331006
25_1639	ctg7180001797261_666_SGT	15331023
25_1640	ctg7180001797261_2664_SAG	15333021
25_1641	ctg7180001797261_7372_SCT	15337729

25_1642	ctg7180001797261_8498_SAC	15338855
25_1643	ctg7180001922482_18052_SAC	15362113
25_1644	ctg7180001922482_16773_SCT	15363392
25_1645	ctg7180001922482_16678_SCT	15363487
25_1646	ctg7180001922482_13126_SAG	15367039
25_1647	ctg7180001922482_10790_SCT	15369375
25_1648	ctg7180001922482_9290_SCT	15370875
25_1649	ctg7180001922482_9166_SCT	15370999
25_1650	ctg7180001922482_9654_SAG	15371412
25_1651	ctg7180001922482_4866_SAG	15375299
25_1652	ctg7180001922482_4002_SCT	15376163
25_1653	ctg7180001812366_8522_SCT	15381964
25_1654	ctg7180001812366_7385_SAG	15383101
25_1655	ctg7180001812366_7305_SAG	15383181
25_1656	ctg7180001812366_7260_SCT	15383226
25_1657	ctg7180001812366_200_SCT	15390286
25_1658	ctg7180001919046_1730_SCT	15401177
25_1659	ctg7180001795475_6430_SAC	15415031
25_1660	ctg7180001563254_343_SCT	15428956
25_1661	ctg7180001563254_7911_SAG	15436524
25_1662	ctg7180001563254_11647_SCT	15440260
25_1663	ctg7180001757567_1786_SCT	15442875
25_1664	ctg7180001829134_4603_SCT	15451349
25_1665	ctg7180001192735_849_SGT	15453944
25_1666	ctg7180001738880_1329_SGT	15462829
25_1667	ctg7180001391836_1335_SCG	15467187
25_1668	ctg7180001799786_4583_SAG	15496703
25_1669	ctg7180001799786_3845_SGT	15497441
25_1670	ctg7180001799786_3722_SAG	15497564
25_1671	ctg7180001799786_3699_SAC	15497587
25_1672	ctg7180001799786_3213_SCT	15498073
25_1673	ctg7180001799786_17405	15500342
25_1674	ctg7180001799786_809_SCT	15500477
25_1675	ctg7180001328235_1864_SAC	15503381
25_1676	ctg7180001328235_3846_SAG	15505363

25_1677	ctg7180001328235_4284_SAC	15505801
25_1678	ctg7180001328235_5850_SAG	15507367
25_1679	ctg7180001826395_3056_SAC	15514269
25_1680	ctg7180001826395_4123_SGT	15515336
25_1681	ctg7180001826395_7173_SGT	15518386
25_1682	ctg7180001826395_11601_SGT	15522814
25_1683	ctg7180001826395_11607_SGT	15522820
25_1684	ctg7180001826395_11713_SGT	15522926
25_1685	ctg7180001806145_4524_SCT	15533645
25_1686	ctg7180001830447_620_SGT	15557417
25_1687	ctg7180001830447_710_SAC	15557507
25_1688	ctg7180001830447_6338_SAG	15563135
25_1689	ctg7180001830447_6872_SGT	15563669
25_1690	ctg7180001919538_9182_SCT	15569435
25_1691	ctg7180001927478_5141_SAG	15583451
25_1692	ctg7180001803755_7036_SAC	15597285
25_1693	ctg7180001803755_7670_SAG	15597919
25_1694	ctg7180001803755_7743_SAC	15597992
25_1695	ctg7180001803755_14929_SAG	15605178
25_1696	ctg7180001803755_15813_SAG	15606062
25_1697	ctg7180001825322_6343_SAG	15609124
25_1698	ctg7180001825322_4198_SAC	15611269
25_1699	ctg7180001825322_3520_SCT	15611947
25_1700	ctg7180001868925_1089_SAC	15619285
25_1701	ctg7180001868925_1624_SCT	15619820
25_1702	ctg7180001868925_2607_SAG	15620803
25_1703	ctg7180001868925_8339_SAG	15626535
25_1704	ctg7180001868925_15857_SAG	15634053
25_1705	ctg7180001920131_17411_SAG	15653217
25_1706	ctg7180001920131_16408_SGT	15654220
25_1707	ctg7180001920131_15308_SCT	15655320
25_1708	ctg7180001815985_3095_SGT	15674875
25_1709	ctg7180001398980_585_SAC	15689770
25_1710	ctg7180001600413_1203_SAG	15691989
25_1711	ctg7180001600413_1329_SAG	15692115

25_1712	ctg7180001600413_1873_SCT	15692659
25_1713	ctg7180001600413_3262_SCT	15694048
25_1714	ctg7180001818228_3010_SCT	15702517
25_1715	ctg7180001818228_3999_SAG	15703506
25_1716	ctg7180001214430_4513_SCT	15717163
25_1717	ctg7180001214430_5882_SAG	15718532
25_1718	ctg7180001447883_5177_SGT	15736838
25_1719	ctg7180001770100_4545_SAG	15742465
25_1720	ctg7180001770100_3598_SAG	15743412
25_1721	ctg7180001770100_2744_SGT	15744266
25_1722	ctg7180001770100_2140_SAG	15744870
25_1723	ctg7180001770100_1868_SAC	15745142
25_1724	ctg7180001834724_12975_SGT	15770644
25_1725	ctg7180001834724_15856_SCT	15773525
25_1726	ctg7180001834724_17070_SAG	15774739
25_1727	ctg7180001834724_18760_SAC	15776429
25_1728	ctg7180001834724_21124_SAC	15778793
25_1729	ctg7180001794168_11347_SAC	15780276
25_1730	ctg7180001430018_97_SCT	15795721
25_1731	ctg7180001642699_1046_SCT	15798351
25_1732	ctg7180001826612_11763_SCT	15834203
25_1733	ctg7180001826612_15584_SGT	15838023
25_1734	ctg7180001926701_2231_SAC	15848848
25_1735	ctg7180001926701_10103_SAG	15856720
25_1736	ctg7180001926701_11188_SGT	15857805
25_1737	ctg7180001723136_1012_SCT	15861955
25_1738	ctg7180001898633_8446_SAC	15906355
25_1739	ctg7180001863436_918_SAC	15935482
25_1740	ctg7180001843708_4774_SCT	15944155
25_1741	ctg7180001843708_5023_SAC	15944404
25_1742	ctg7180001794300_3545_SCT	15950514
25_1743	ctg7180001794300_7085_SGT	15954054
25_1744	ctg7180001794300_7328_SAC	15954297
25_1745	ctg7180001794300_9247_SAG	15956216
25_1746	ctg7180001898543_7625_SAG	15967815

25_1747	ctg7180001811536_5717_SAC	15991088
25_1748	ctg7180001811536_12548_SAC	15997919
25_1749	ctg7180001811536_12555_SGT	15997926
25_1750	ctg7180001791100_1957_SCT	16035674
25_1751	ctg7180001791102_3343_SCT	16043848
25_1752	ctg7180001834705_1627_SAG	16053998
25_1753	ctg7180001834705_3912_SCT	16056283
25_1754	ctg7180001834705_17113_SGT	16069480
25_1755	ctg7180001812439_9267_SAG	16077958
25_1756	ctg7180001812439_2845_SAG	16084380
25_1757	ctg7180001812439_2423_SGT	16084802
25_1758	ctg7180001335434_2391_SCT	16102208
25_1759	ctg7180001335434_1848_SGT	16102751
25_1760	ctg7180001822568_5831_SAG	16112001
25_1761	ctg7180001822568_5116_SAG	16112716
25_1762	ctg7180001822568_3811_SAG	16114021
25_1763	ctg7180001866739_5135_SAG	16128396
25_1764	ctg7180001903350_12865_SAG	16147800
25_1765	ctg7180001903350_13763_SAG	16148698
25_1766	ctg7180001903350_15439_SAG	16150374
25_1767	ctg7180001903350_20761_SAG	16155696
25_1768	ctg7180001903350_22272_SGT	16157207
25_1769	ctg7180001871908_3392_SCT	16166470
25_1770	ctg7180001929281_13316_SAG	16173997
25_1771	ctg7180001929281_12999_SCT	16174314
25_1772	ctg7180001929281_10314_SCT	16177357
25_1773	ctg7180001929281_4912_SGT	16182759
25_1774	ctg7180001285699_2960_SCT	16201868
25_1775	ctg7180001210087_2798_SAG	16208664
25_1776	ctg7180001889088_4525_SAG	16229971
25_1777	ctg7180001889088_1711_SCT	16232785
25_1778	ctg7180001889088_579_SCT	16233917
25_1779	ctg7180001844987_6130_SAG	16258264
25_1780	ctg7180001844987_2545_SCT	16259942
25_1781	ctg7180001214664_4397_SGT	16263383

25_1782	ctg7180001214664_4197_SAG	16263583
25_1783	ctg7180001214664_3975_SGT	16263805
25_1784	ctg7180001214664_2288_SCT	16265492
25_1785	ctg7180001905133_3534_SGT	16270882
25_1786	ctg7180001919185_6061_SCT	16278062
25_1787	ctg7180001919185_3138_SCT	16280985
25_1788	ctg7180001919185_2083_SGT	16282040
25_1789	ctg7180001846268_8805_SCT	16296558
25_1790	ctg7180001458363_2382_SCT	16310929
25_1791	ctg7180001458363_2514	16311061
25_1792	ctg7180001458363_2696_SAG	16311208
25_1793	ctg7180001458363_4400_SCT	16312912
25_1794	ctg7180001458363_5570_SGT	16314082
25_1795	ctg7180001458363_5643_SCT	16314155
25_1796	ctg7180001299407_4361_SCT	16331118
25_1797	GCR_cBin43301_Ctg1_366	16351501
25_1798	ctg7180001537178_1594_SAG	16351901
25_1799	ctg7180001736161_63_SGT	16355663
25_1800	ctg7180001736161_683_SCT	16356283
25_1801	ctg7180001868394_21533_SCT	16378625
25_1802	ctg7180001868394_28356_SCT	16385448
25_1803	ctg7180001813015_1739_SGT	16400159
25_1804	ctg7180001390441_687_SAC	16406077
25_1805	ctg7180001800255_493_SCT	16407321
25_1806	ctg7180001800255_1211_SCT	16408039
25_1807	ctg7180001599310_1864_SCT	16459210
25_1808	ctg7180001261835_1111_SGT	16480484
25_1809	ctg7180001920244_1046_SAC	16524673
25_1810	ctg7180001437221_156_SGT	16534610
25_1811	ctg7180001935720_6480_SCT	16547056
25_1812	ctg7180001935720_5554_SAG	16547982
25_1813	ctg7180001935720_3599_SCT	16549937
25_1814	ctg7180001930619_8391_SAG	16562280
25_1815	ctg7180001930619_13483_SGT	16567372
25_1816	ctg7180001794728_5140_SAG	16581563

25_1817	ctg7180001900180_3438_SCT	16608930
25_1818	ctg7180001900180_6238_SAG	16611730
25_1819	ctg7180001900180_6427_SAG	16611919
25_1820	ctg7180001900180_7171_SAC	16612663
25_1821	ctg7180001900180_14822_SCT	16620314
25_1822	ctg7180001887127_2742_SCT	16632121
25_1823	ctg7180001921700_7929_SCT	16635840
25_1824	ctg7180001921700_7381_SGT	16636387
25_1825	ctg7180001861437_4875_SCT	16681818
25_1826	ctg7180001861437_4703_SAG	16681990
25_1827	ctg7180001861437_3954_SGT	16682739
25_1828	ctg7180001928786_3992_SAC	16687976
25_1829	ctg7180001928786_7090_SGT	16691074
25_1830	ctg7180001928786_8856_SCT	16692839
25_1831	ctg7180001791507_421_SCT	16695552
25_1832	ctg7180001791507_1250_SAC	16696381
25_1833	ctg7180001904696_9187_SCT	16721847
25_1834	ctg7180001898513_2933_SGT	16747609
25_1835	ctg7180001834091_8638_SAG	16754462
25_1836	ctg7180001834091_4005	16754523
25_1837	ctg7180001834091_8015_SGT	16755085
25_1838	ctg7180001834091_6654_SAG	16756446
25_1839	ctg7180001834091_4469_SCT	16758631
25_1840	ctg7180001834091_2373_SAG	16760727
25_1841	ctg7180001834091_2060_SGT	16761040
25_1842	ctg7180001834091_1640_SCT	16761460
25_1843	ctg7180001834091_1516_SAG	16761584
25_1844	ctg7180001834091_834_SCT	16762266
25_1845	ctg7180001834091_674_SAG	16762426
25_1846	ctg7180001805253_7689_SCT	16829284
25_1847	ctg7180001559773_7468_SAC	16845425
25_1848	ctg7180001927291_9345_SCT	16859705
25_1849	ctg7180001927291_7383_SCT	16861667
25_1850	ctg7180001927291_6932_SCT	16862118
25_1851	ctg7180001927291_6890_SGT	16862160

25_1852	ctg7180001927291_5196_SCT	16863854
25_1853	ctg7180001550307_1623_SAC	16890308
25_1854	ctg7180001550307_1706_SGT	16890391
25_1855	ctg7180001913148_989_SAG	16895943
25_1856	ctg7180001934635_9908_SAC	16903827
25_1857	ctg7180001934635_1651_SAC	16912085
25_1858	ctg7180001934635_12565_SAG	16913080
25_1859	ctg7180001933426_24142_SGT	16941775
25_1860	ctg7180001899908_352_SGT	16991301
25_1861	ctg7180001899908_12305_SGT	17003254
25_1862	ctg7180001924415_5047_SAG	17030237
25_1863	ctg7180001920498_1714_SAG	17055603
25_1864	ctg7180001812919_5751_SAC	17109959
25_1865	ctg7180001812919_5957_SGT	17110165
25_1866	ctg7180001812919_6246_SCT	17110454
25_1867	ctg7180001812919_8008_SAG	17112216
25_1868	ctg7180001812919_10486_SCT	17114694
25_1869	ctg7180001902771_576_SCT	17120590
25_1870	ctg7180001449907_2035_SAG	17142528
25_1871	ctg7180001826822_4685_SAG	17152214
25_1872	ctg7180001826822_2794_SCT	17154105
25_1873	ctg7180001826822_269_SGT	17156630
25_1874	ctg7180001834134_2659_SGT	17163046
25_1875	ctg7180001843406_2778_SAG	17170579
25_1876	ctg7180001843406_3274_SGT	17171075
25_1877	ctg7180001843406_10720_SAC	17178521
25_1878	ctg7180001843406_11064_SCT	17178865
25_1879	ctg7180001487612_229_SCT	17203411
25_1880	ctg7180001453538_136_SCT	17240780
25_1881	ctg7180001874650_10872_SCT	17255670
25_1882	ctg7180001797803_12827_SAG	17294665
25_1883	ctg7180001797803_7951_SCT	17299541
25_1884	ctg7180001797803_7285_SAG	17300207
25_1885	ctg7180001797803_6035_SGT	17301457
25_1886	ctg7180001797803_5806_SCT	17301686

25_1887	ctg7180001797803_4742_SCT	17302750
25_1888	ctg7180001797803_4368_SAC	17303124
25_1889	ctg7180001249557_1622_SAG	17309060
25_1890	ctg7180001833679_5028_SAC	17343568
25_1891	ctg7180001833679_5817_SAC	17344357
25_1892	ctg7180001327339_373_SAG	17367729
25_1893	ctg7180001841434_1242_SAG	17374918
25_1894	ctg7180001834484_261_SAG	17414905
25_1895	ctg7180001843196_8205_SGT	17431508
25_1896	ctg7180001843196_8573_SGT	17431876
25_1897	ctg7180001815639_4102_SGT	17438972
25_1898	ctg7180001861759_2796_SGT	17449763
25_1899	ctg7180001873645_6623_SGT	17500804
25_1900	ctg7180001459100_515_SAG	17507965
25_1901	ctg7180001459100_5295_SAG	17512745
25_1902	ctg7180001459100_5421_SGT	17512871
25_1903	ctg7180001459100_8405_SAG	17515855
25_1904	ctg7180001459100_9501_SCT	17516951
25_1905	ctg7180001911752_25613_SAG	17542473
25_1906	ctg7180001911752_21638_SAC	17546448
25_1907	ctg7180001911752_21581_SAG	17546505
25_1908	ctg7180001911752_20818_SAG	17547268
25_1909	ctg7180001911752_20660_SAG	17547426
25_1910	ctg7180001911752_20605_SGT	17547481
25_1911	ctg7180001911752_18316_SAC	17549770
25_1912	ctg7180001911752_16412_SCT	17551674
25_1913	ctg7180001911752_10773_SGT	17557313
25_1914	ctg7180001911752_7153_SAC	17560933
25_1915	ctg7180001801965_5706_SAG	17571032
25_1916	ctg7180001911751_10349_SAG	17579929
25_1917	ctg7180001911751_9706_SAC	17580572
25_1918	ctg7180001911751_821_SGT	17589458
25_1919	ctg7180001923001_272_SCT	17598127
25_1920	ctg7180001923001_3349_SCT	17601204
25_1921	ctg7180001844772_206_SCT	17620070

25_1922	ctg7180001844772_2004_SGT	17621868
25_1923	ctg7180001844772_2447_SCT	17622311
25_1924	ctg7180001844772_5101_SAC	17624965
25_1925	ctg7180001696354_3684_SAC	17680425
25_1926	ctg7180001896881_3121_SCT	17694239
25_1927	ctg7180001896881_3950_SAG	17695068
25_1928	ctg7180001896882_414_SCT	17698173
25_1929	ctg7180001896882_652_SAC	17698411
25_1930	ctg7180001896882_784_SAG	17698543
25_1931	ctg7180001928113_5582_SCT	17709988
25_1932	ctg7180001854051_17692_SCT	17750854
25_1933	ctg7180001562053_447_SAG	17779044
25_1934	ctg7180001575198_1207_SCT	17921383
25_1935	ctg7180001679061_5250_SAG	17935171
25_1936	ctg7180001679061_6545 17936501	
25_1937	ctg7180001386207_363_SCT	17956058
25_1938	ctg7180001790217_7606_SAG	17986866
25_1939	ctg7180001545895_400_SAG	18003597
25_1940	ctg7180001860645_15766_SAG	18005673
25_1941	ctg7180001860645_11635_SAG	18009804
25_1942	ctg7180001860645_7264_SCT	18014175
25_1943	ctg7180001860645_2560_SGT	18018879
25_1944	ctg7180001786538_227_SGT	18022091
25_1945	ctg7180001291293_1451_SCT	18040545
25_1946	ctg7180001291210_2440_SCT	18044250
25_1947	ctg7180001860644_6574_SAG	18068005
25_1948	ctg7180001860644_4134_SAG	18070445
25_1949	ctg7180001699795_667_SAG	18090322
25_1950	ctg7180001267682_926_SCT	18092405
25_1951	ctg7180001233336_1710_SAG	18121993
25_1952	ctg7180001445712_2858_SGT	18137002
25_1953	ctg7180001817312_4025_SCT	18165375
25_1954	ctg7180001881608_14447_SAG	18180705
25_1955	ctg7180001855879_12482_SAC	18221306
25_1956	ctg7180001804883_29408_SCT	18279739

25_1957	ctg7180001804883_28048_SAG	18281099
25_1958	ctg7180001804883_24967_SAG	18284180
25_1959	ctg7180001919866_2081_SCT	18328924
25_1960	ctg7180001810268_10484_SCT	18338068
25_1961	ctg7180001835136_1732_SGT	18355735
25_1962	ctg7180001686912_62_SCT	18376537
25_1963	ctg7180001913739_12546_SCT	18398865
25_1964	ctg7180001795413_2023_SAC	18412028
25_1965	ctg7180001795413_1703_SAG	18412348
25_1966	ctg7180001795113_9939_SCT	18426505
25_1967	ctg7180001795113_10622_SAC	18427188
25_1968	ctg7180001825254_84_SAG	18433301
25_1969	ctg7180001269492_4196_SCT	18440750
25_1970	ctg7180001826145_12468_SCT	18448052
25_1971	ctg7180001826145_11067_SAG	18449453
25_1972	ctg7180001826145_9875_SCT	18450645
25_1973	ctg7180001467630_5104_SGT	18473523
25_1974	ctg7180001467630_3793_SAG	18474834
25_1975	ctg7180001467630_2911_SAG	18475716
25_1976	ctg7180001916943_1611_SCT	18482309
25_1977	ctg7180001916943_7343_SAG	18488041
25_1978	ctg7180001916943_11837_SAC	18492535
25_1979	ctg7180001916943_26986_SGT	18507685
25_1980	ctg7180001916943_26989_SAC	18507688
25_1981	ctg7180001916943_42676_SAC	18523375
25_1982	ctg7180001542568_2855_SAG	18538477
25_1983	ctg7180001542568_2766_SCT	18538566
25_1984	ctg7180001542568_2729_SAG	18538603
25_1985	ctg7180001542568_994_SCT	18540338
25_1986	ctg7180001502479_848_SCT	18542166
25_1987	ctg7180001502479_979_SAC	18542297
25_1988	ctg7180001916945_6708_SAG	18550234
25_1989	ctg7180001916945_7019_SGT	18550545
25_1990	ctg7180001935006_4011_SAC	18556637
25_1991	ctg7180001935006_8103_SCT	18560729

25_1992	ctg7180001935006_8778_SCT	18561439
25_1993	ctg7180001570425_3057_SCT	18570597
25_1994	ctg7180001873544_18024_SGT	18579287
25_1995	ctg7180001443474_585_SCT	18598031
25_1996	ctg7180001853206_4276_SCT	18621350
25_1997	ctg7180001650116_9955_SGT	18643489
25_1998	ctg7180001650116_10745_SAG	18644279
25_1999	ctg7180001650116_10947_SCT	18644481
25_2000	ctg7180001696022_2142_SGT	18647779
25_2001	ctg7180001527715_245_SCT	18648978
25_2002	ctg7180001887062_2412_SCT	18659127
25_2003	ctg7180001736496_1077_SAG	18663166
25_2004	ctg7180001793476_2789_SGT	18669474
25_2005	ctg7180001793476_4445_SGT	18671130
25_2006	ctg7180001793476_7787_SCT	18674472
25_2007	ctg7180001793476_7873_SAG	18674558
25_2008	ctg7180001793476_15829_SGT	18682514
25_2009	ctg7180001329038_2054_SCT	18689987
25_2010	ctg7180001896806_12660_SAG	18705287
25_2011	ctg7180001896806_6134_SAG	18711814
25_2012	ctg7180001859244_5798_SAG	18731481
25_2013	ctg7180001859244_5123_SGT	18732156
25_2014	ctg7180001859244_4648_SAG	18732631
25_2015	ctg7180001859244_1512_SCT	18735767
25_2016	ctg7180001859244_1304_SCT	18735975
25_2017	ctg7180001859244_818_SCT	18736461
25_2018	ctg7180001821756_11990_SAC	18740045
25_2019	ctg7180001821756_3500_SAG	18748535
25_2020	ctg7180001856149_7836_SGT	18776104
25_2021	ctg7180001329211_13415_SCT	18785316
25_2022	ctg7180001329211_13215_SGT	18785516
25_2023	ctg7180001299431_727_SCT	18799782
25_2024	ctg7180001925308_8504_SCT	18809729
25_2025	ctg7180001925308_8118_SCT	18810115
25_2026	ctg7180001474484_8081_SAG	18849907

25_2027	ctg7180001911575_5068_SAC	18859322
25_2028	ctg7180001911575_12387_SCG	18866641
25_2029	ctg7180001924893_812	18873068
25_2030	ctg7180001924893_13695_SAG	18885916
25_2031	ctg7180001924893_14787_SCT	18887008
25_2032	ctg7180001924893_16608_SAC	18888829
25_2033	ctg7180001924893_16726_SCT	18888947
25_2034	ctg7180001924893_17248_SAG	18889469
25_2035	ctg7180001763522_6453_SGT	18894409
25_2036	ctg7180001763522_6434_SAG	18894428
25_2037	ctg7180001820029_5044_SAG	18905911
25_2038	ctg7180001794635_25990_SCT	18953300
25_2039	ctg7180001918108_18585_SGT	18954428
25_2040	ctg7180001918108_17371_SAG	18955642
25_2041	ctg7180001918108_7626_SGT	18965387
25_2042	ctg7180001918108_6501_SAG	18966512
25_2043	ctg7180001918108_6023_SCT	18966990
25_2044	ctg7180001918108_5995_SAG	18967018
25_2045	ctg7180001811785_3571_SGT	18980939
25_2046	ctg7180001811785_1794_SAG	18982717
25_2047	ctg7180001341116_146_SAG	19038775
25_2048	ctg7180001643209_2345_SAC	19064298
25_2049	ctg7180001643209_1044_SAG	19065599
25_2050	ctg7180001892824_12806_SCT	19080508
25_2051	ctg7180001892825_5074_SAC	19095732
25_2052	ctg7180001892825_5938_SAG	19096596
25_2053	ctg7180001745826_2484_SAG	19103623
25_2054	ctg7180001745826_1371_SCT	19104736
25_2055	ctg7180001897016_3216_SAC	19124642
25_2056	ctg7180001483933_1847_SCT	19157380
25_2057	ctg7180001483933_1364_SCT	19157863
25_2058	ctg7180001795135_6344_SCT	19180960
25_2059	ctg7180001795135_6471_SGT	19181087
25_2060	ctg7180001828312_5421_SAG	19200331
25_2061	ctg7180001828312_2418_SGT	19203334

25_2062	ctg7180001841556_3234_SAC	19213699
25_2063	ctg7180001927563_2427_SCT	19235929
25_2064	ctg7180001272989_846_SAG	19271329
25_2065	ctg7180001936139_9480_SCT	19277417
25_2066	ctg7180001936139_9161_SCT	19277736
25_2067	ctg7180001936139_9111_SCT	19277786
25_2068	ctg7180001936139_7778_SGT	19279119
25_2069	ctg7180001238976_1332_SAG	19293470
25_2070	ctg7180001871645_13966_SCT	19299551
25_2071	ctg7180001914645_4944_SGT	19346257
25_2072	ctg7180001870515_184_SAG	19386479
25_2073	ctg7180001870515_226_SAC	19386521
25_2074	ctg7180001870516_1186_SGT	19395790
25_2075	ctg7180001870516_1536_SAC	19396140
25_2076	ctg7180001870516_9025_SGT	19403629
25_2077	ctg7180001870516_9378_SCT	19403982
25_2078	ctg7180001870516_9433_SCT	19404037
25_2079	ctg7180001920558_8737_SGT	19430229
25_2080	ctg7180001920558_11529_SAG	19433021
25_2081	ctg7180001920558_12129_SCT	19433621
25_2082	ctg7180001895355_7785_SAG	19444955
25_2083	ctg7180001926317_9340_SAC	19485441
25_2084	ctg7180001926317_5742_SCT	19489039
25_2085	ctg7180001926317_4925_SAG	19489856
25_2086	ctg7180001446702_1092_SCT	19500428
25_2087	ctg7180001861234_23075_SAC	19514848
25_2088	ctg7180001861234_21372_SAG	19516551
25_2089	ctg7180001334706_70_SAC	19533884
25_2090	ctg7180001334706_1288_SAG	19535102
25_2091	ctg7180001452831_4153_SGT	19541114
25_2092	ctg7180001452831_4798_SAC	19541759
25_2093	ctg7180001918913_6971_SGT	19547925
25_2094	ctg7180001807564_18555_SCT	19560306
25_2095	ctg7180001807564_11203	19560546
25_2096	ctg7180001807564_17268_SAG	19561593

25_2097	ctg7180001807564_10330_SCT	19568531
25_2098	ctg7180001807564_6811_SAC	19572050
25_2099	ctg7180001805689_1927_SCT	19592877
25_2100	ctg7180001934203_2234_SAG	19609921
25_2101	ctg7180001934203_16892_SAG	19624578
25_2102	ctg7180001934203_19937_SCT	19627623
25_2103	ctg7180001672408_12938_SCT	19634617
25_2104	ctg7180001672408_12476_SAG	19635079
25_2105	ctg7180001672408_11769_SAC	19635786
25_2106	ctg7180001808628_3509_SCT	19660146
25_2107	ctg7180001814682_5564_SCT	19678462
25_2108	ctg7180001805390_2375_SGT	19698745
25_2109	ctg7180001805390_1337_SGT	19699783
25_2110	ctg7180001792711_2479_SGT	19710686
25_2111	ctg7180001902348_10421_SCT	19731692
25_2112	ctg7180001256096_1475_SAG	19749155
25_2113	ctg7180001819425_1491_SCT	19789427
25_2114	ctg7180001895633_5771_SAG	19800654
25_2115	ctg7180001895548_6186_SAG	19812921
25_2116	ctg7180001270621_89_SAG	19817591
25_2117	GCR_cBin23464_Ctg1_501	19825365
25_2118	ctg7180001817452_296_SCT	19827046
25_2119	ctg7180001817452_2267_SCT	19829017
25_2120	ctg7180001817452_2402_SAG	19829152
25_2121	ctg7180001906629_9532_SCT	19835783
25_2122	ctg7180001906629_9002_SAG	19836313
25_2123	ctg7180001906629_6933_SGT	19838382
25_2124	ctg7180001906629_4470_SAG	19840845
25_2125	ctg7180001697324_4845_SAC	19851450
25_2126	ctg7180001269808_1435_SAG	19871444
25_2127	ctg7180001632169_7157_SAG	19876223
25_2128	ctg7180001632169_7117_SAC	19876263
25_2129	ctg7180001632169_5810_SAG	19877570
25_2130	ctg7180001632169_5376_SAC	19878004
25_2131	ctg7180001830187_2431_SGT	19892575

25_2132	ctg7180001899964_4383_SGT	19899359
25_2133	ctg7180001899964_3438_SCT	19900304
25_2134	ctg7180001751215_1787_SGT	19911047
25_2135	ctg7180001854609_2930_SCT	19920012
25_2136	ctg7180001852674_3889_SCT	19933786
25_2137	ctg7180001852674_2511_SAC	19935164
25_2138	ctg7180001889072_1024_SCT	19942267
25_2139	ctg7180001889072_3884_SCT	19944913
25_2140	ctg7180001889072_975_SAG	19947822
25_2141	ctg7180001889072_254_SAG	19948543
25_2142	ctg7180001921212_8940_SCT	19976152
25_2143	ctg7180001921212_3433_SAG	19977228
25_2144	ctg7180001921212_3764_SCT	19981328
25_2145	ctg7180001430807_3076_SGT	19998199
25_2146	ctg7180001641258_4860_SAC	20030136
25_2147	ctg7180001445681_128_SCT	20037330
25_2148	ctg7180001578824_768_SAG	20046696
25_2149	ctg7180001578824_1265_SCT	20047193
25_2150	ctg7180001282094_235_SAC	20119489
25_2151	ctg7180001814954_872_SAG	20147311
25_2152	ctg7180001667091_473_SGT	20149916
25_2153	ctg7180001283014_1533_SCT	20175017
25_2154	ctg7180001563471_8209_SAG	20182830
25_2155	ctg7180001563471_1876_SGT	20189163
25_2156	ctg7180001563471_1209_SAC	20189830
25_2157	ctg7180001191992_438_SCT	20200045
25_2158	ctg7180001334321_1086_SCT	20209816
25_2159	ctg7180001341246_84_SCT	20255254
25_2160	ctg7180001921539_2504_SGT	20294210
25_2161	ctg7180001916901_5669_SCT	20303042
25_2162	ctg7180001916901_8098_SAC	20305471
25_2163	ctg7180001553736_126_SAC	20313028
25_2164	ctg7180001706031_390_SAC	20323777
25_2165	ctg7180001852913_5793_SAG	20363498
25_2166	ctg7180001915329_10017_SCT	20369399

25_2167	ctg7180001190145_1126	20394886
25_2168	ESTNV_35473_1871	20394976
25_2169	ctg7180001269516_1278_SCT	20412739
25_2170	ctg7180001269516_1413_SAG	20412874
25_2171	ctg7180001269516_1481_SGT	20412942
25_2172	ctg7180001269516_2424_SAC	20413885
25_2173	ctg7180001269516_3425_SAG	20414886
25_2174	ctg7180001269516_3441_SCT	20414902
25_2175	ctg7180001856821_534_SGT	20427784
25_2176	ctg7180001856821_11011_SAC	20438259
25_2177	ctg7180001856822_8641_SCT	20461104
25_2178	ctg7180001525881_4571_SCT	20474204
25_2179	ctg7180001525881_3789_SCT	20475184
25_2180	ctg7180001525881_3062_SGT	20475910
25_2181	ctg7180001892842_767_SAG	20485599
25_2182	ctg7180001766282_1232_SAC	20534019
25_2183	ctg7180001890000_1106_SAC	20542986
25_2184	ctg7180001890000_1608_SAG	20543488
25_2185	ctg7180001824305_1649_SAG	20600134
25_2186	ctg7180001808021_1018_SAC	20622755
25_2187	ctg7180001600740_1378_SAG	20629220
25_2188	ctg7180001190772_4101_SGT	20658184
25_2189	ctg7180001851296_1080_SCT	20663067
25_2190	ctg7180001645977_2887_SGT	20695619
25_2191	ctg7180001597614_2241_SAG	20726316
25_2192	ctg7180001545719_1356_SCT	20726901
25_2193	ctg7180001879907_4025_SAG	20748009
25_2194	ctg7180001879907_1885_SGT	20750149
25_2195	ctg7180001798602_1653_SAG	20777947
25_2196	ctg7180001798602_5338_SCT	20781632
25_2197	ctg7180001798602_5971_SAC	20782265
25_2198	ctg7180001798602_6358_SAC	20782652
25_2199	ctg7180001575028_52_SAG	20784952
25_2200	ctg7180001575028_1788_SCT	20786688
25_2201	ctg7180001381275_1470_SAG	20789226

25_2202	ctg7180001567797_17807_SAG	20791979
25_2203	ctg7180001567797_11937_SGT	20797849
25_2204	ctg7180001567797_9785_SAG	20800001
25_2205	ctg7180001567797_9621_SCT	20800165
25_2206	ctg7180001567797_7876_SAG	20801910
25_2207	ctg7180001567797_7631_SCT	20802155
25_2208	ctg7180001567797_7274_SAG	20802512
25_2209	ctg7180001567797_7249_SAC	20802537
25_2210	ctg7180001567797_7094_SGT	20802692
25_2211	ctg7180001567797_5810_SAG	20803976
25_2212	ctg7180001860429_588_SCT	20812324
25_2213	ctg7180001860428_449_SAG	20821455
25_2214	ctg7180001905032_8967_SAC	20837698
25_2215	ctg7180001241672_1557_SAC	20893338
25_2216	ctg7180001391618_638_SCT	20918751
25_2217	ctg7180001391618_723_SGT	20918836
25_2218	ctg7180001848498_1006_SCT	20937698
25_2219	ctg7180001848498_1023_SAC	20937715
25_2220	ctg7180001773830_6506_SCT	20941077
25_2221	ctg7180001639293_3549_SGT	20956148
25_2222	ctg7180001639293_3738_SAG	20956373
25_2223	ctg7180001639293_3949_SAG	20956548
25_2224	ctg7180001834654_7618_SAC	20973179
25_2225	ctg7180001834654_10912_SCT	20976473
25_2226	ctg7180001834654_16963_SGT	20982524
25_2227	ctg7180001834654_17220	20982816
25_2228	ctg7180001834654_17371_SAC	20982932
25_2229	ctg7180001790422_1167_SAC	20985980
25_2230	ctg7180001790422_2220_SCT	20987033
25_2231	ctg7180001790422_3234_SAG	20988047
25_2232	ctg7180001935563_5696_SCT	21015192
25_2233	ctg7180001789680_68_SCT	21031218
25_2234	ctg7180001789678_3963_SAG	21035080
25_2235	ctg7180001789678_2060_SCT	21036983
25_2236	ctg7180001789678_1909_SGT	21037134

25_2237	ctg7180001901658_1775_SGT	21041004
25_2238	ctg7180001224859_832_SAG	21045745
25_2239	ctg7180001889172_449_SGT	21046257
25_2240	ctg7180001889172_4546_SCT	21050354
25_2241	ctg7180001889172_9196_SAG	21055004
25_2242	ctg7180001889172_9692_SAC	21055500
25_2243	ctg7180001889172_11079_SGT	21056887
25_2244	ctg7180001889172_13364_SGT	21059172
25_2245	ctg7180001833295_17660_SCT	21090433
25_2246	ctg7180001833295_1674_SAG	21106419
25_2247	ctg7180001823577_1444_SAG	21112556
25_2248	ctg7180001823577_2084_SAG	21113196
25_2249	ctg7180001823577_2153_SAG	21113265
25_2250	ctg7180001823577_2203_SGT	21113315
25_2251	ctg7180001823577_12194_SAC	21123306
25_2252	ctg7180001823577_14857_SCT	21125969
25_2253	ctg7180001797294_6301_SAC	21133396
25_2254	ctg7180001811035_3880_SCT	21139675
25_2255	ctg7180001811035_3808_SCT	21139747
25_2256	ctg7180001381434_2629_SCT	21146488
25_2257	ctg7180001807626_20182_SGT	21160697
25_2258	ctg7180001807626_6031_SAG	21174889
25_2259	ctg7180001909670_11250_SAC	21243615
25_2260	ctg7180001928231_7878_SAG	21254771
25_2261	ctg7180001801910_5094_SAC	21275256
25_2262	ctg7180001312822_423_SCT	21322062
25_2263	ctg7180001316671_2081_SAG	21325251
25_2264	ctg7180001316671_2260_SAG	21325430
25_2265	ctg7180001801332_4222_SGT	21366092
25_2266	ctg7180001184194_950_SAG	21386583
25_2267	ctg7180001870139_12609_SCT	21417445
25_2268	ctg7180001266423_212_SAG	21443852
25_2269	ctg7180001266423_1796_SAG	21445436
25_2270	ctg7180001374284_1199_SGT	21450597
25_2271	ctg7180001374284_4447_SAC	21451174

25_2272	ctg7180001374284_3061_SCT	21452560
25_2273	ctg7180001364486_652_SGT	21458561
25_2274	ctg7180001935877_6818_SGT	21473657
25_2275	ctg7180001935877_9802_SAG	21476641
25_2276	ctg7180001611627_875_SCT	21501636
25_2277	ctg7180001807765_5176_SAG	21510103
25_2278	ctg7180001807765_6324_SAG	21511251
25_2279	ctg7180001807765_8552_SAG	21513479
25_2280	ctg7180001807765_12239_SCT	21517166
25_2281	ctg7180001807765_12602_SCT	21517529
25_2282	ctg7180001686347_3940_SAC	21520585
25_2283	ctg7180001686347_3880_SCT	21520645
25_2284	ctg7180001686347_3850_SGT	21520675
25_2285	ctg7180001686347_3432_SAC	21521093
25_2286	ctg7180001686347_3288_SAG	21521237
25_2287	ctg7180001686347_1121_SAG	21523404
25_2288	ctg7180001650968_4164_SCT	21526767
25_2289	ctg7180001650968_4053_SCT	21526878
25_2290	ctg7180001650968_3376_SCT	21527555
25_2291	ctg7180001650968_2266_SAC	21528665
25_2292	ctg7180001650968_1794_SCT	21529137
25_2293	ctg7180001650968_753_SAG	21530178
25_2294	ctg7180001432126_177_SAC	21534653
25_2295	ctg7180001793408_13362_SAG	21535839
25_2296	ctg7180001793408_12985_SAG	21536216
25_2297	ctg7180001793408_12890_SAC	21536311
25_2298	ctg7180001793408_10803_SGT	21538398
25_2299	ctg7180001793408_10559_SAG	21538642
25_2300	ctg7180001793408_10282_SGT	21538919
25_2301	ctg7180001793408_8546_SAG	21540655
25_2302	ctg7180001793408_5023_SAG	21544178
25_2303	ctg7180001793408_4069_SCT	21545132
25_2304	ctg7180001793408_3952_SAC	21545249
25_2305	ctg7180001290274_1702_SAC	21551470
25_2306	ctg7180001793379_2965_SCT	21569303

25_2307	ctg7180001793379_4362_SAG	21570700
25_2308	ctg7180001793379_7627_SAG	21573965
25_2309	ctg7180001793379_9896_SGT	21576234
25_2310	ctg7180001793379_13921_SGT	21580259
25_2311	ctg7180001793379_23853_SAC	21590191
25_2312	ctg7180001807506_286_SGT	21620702
25_2313	ctg7180001807506_360_SAG	21620776
25_2314	ctg7180001807506_515_SAG	21620931
25_2315	ctg7180001807506_3330_SAG	21623746
25_2316	ctg7180001807506_3608_SAG	21624024
25_2317	ctg7180001807506_4303_SAG	21624719
25_2318	ctg7180001807506_13109_SCT	21633525
25_2319	ctg7180001807506_16610_SAG	21637026
25_2320	ctg7180001859114_3165_SAG	21647188
25_2321	ctg7180001322825_594_SGT	21651932
25_2322	ctg7180001920867_1607 21654303	
25_2323	ctg7180001920867_12756_SCT	21656176
25_2324	ctg7180001920867_11975_SAG	21656957
25_2325	ctg7180001920867_9538_SAG	21659394
25_2326	ctg7180001920867_9476_SCT	21659456
25_2327	ctg7180001920867_9292_SCT	21659640
25_2328	ctg7180001920867_8812_SGT	21660120
25_2329	ctg7180001920867_5618_SAC	21663314
25_2330	ctg7180001349905_1036_SAG	21671514
25_2331	ctg7180001862705_772_SGT	21674694
25_2332	ctg7180001862705_801_SAG	21674723
25_2333	ctg7180001862705_971_SGT	21674893
25_2334	ctg7180001862705_1046_SAG	21674968
25_2335	ctg7180001823360_2441_SGT	21691755
25_2336	ctg7180001823360_2587_SCT	21691901
25_2337	ctg7180001823360_6153_SAC	21695467
25_2338	ctg7180001491847_653_SAC	21704665
25_2339	ctg7180001491847_573_SAG	21704745
25_2340	ctg7180001491847_469_SAC	21704849
25_2341	ctg7180001878791_352_SCT	21710003

25_2342	ctg7180001878791_493_SAG	21710144
25_2343	ctg7180001878791_827_SAG	21710478
25_2344	ctg7180001829891_2226_SCT	21728454
25_2345	ctg7180001491844_295_SAG	21734377
25_2346	ctg7180001491844_2909_SGT	21736991
25_2347	ctg7180001491844_3203_SCT	21737285
25_2348	ctg7180001491844_3360_SAC	21737442
25_2349	ctg7180001817127_3063_SCT	21763602
25_2350	ctg7180001817127_3220_SGT	21763759
25_2351	ctg7180001184800_2270_SGT	21782596
25_2352	ctg7180001184800_2608_SCT	21782934
25_2353	ctg7180001184800_2633_SAG	21782959
25_2354	ctg7180001184800_2817_SAG	21783143
25_2355	ctg7180001184800_3767_SCT	21784093
25_2356	ctg7180001429970_1274_SCT	21792788
25_2357	ctg7180001436891_440_SCT	21794698
25_2358	ctg7180001340822_1087_SCT	21815857
25_2359	ctg7180001861431_618_SAG	21827509
25_2360	ctg7180001861431_2233_SCT	21829124
25_2361	ctg7180001311432_4813_SCT	21834795
25_2362	ctg7180001311432_527_SGT	21839081
25_2363	ctg7180001796050_1612_SAC	21855213
25_2364	ctg7180001627531_895_SAG	21858848
25_2365	ctg7180001257539_4482_SGT	21864636
25_2366	ctg7180001294741_8006_SAG	21884957
25_2367	ctg7180001881515_6475_SGT	21920957
25_2368	ctg7180001903776_1712_SAG	21959796
25_2369	ctg7180001223066_1043_SCT	22003303
25_2370	ctg7180001624767_4399_SCT	22010932
25_2371	ctg7180001624767_3721_SCT	22011610
25_2372	ctg7180001624767_1738_SAG	22013593
25_2373	ctg7180001624767_1634_SGT	22013697
25_2374	ctg7180001873466_853_SAG	22016401
25_2375	ctg7180001873466_1109_SAG	22016657
25_2376	ctg7180001873466_1956_SAC	22017504

25_2377	ctg7180001923844_4458_SAG	22073247
25_2378	ctg7180001923844_5281_SAC	22074070
25_2379	ctg7180001923844_14623_SAC	22083412
25_2380	ctg7180001288750_1010_SCT	22095577
25_2381	ctg7180001288750_625_SGT	22095962
25_2382	ctg7180001821415_5215_SAG	22107846
25_2383	ctg7180001821415_6691_SGT	22109322
25_2384	ctg7180001821415_6954_SAG	22109585
25_2385	ctg7180001821415_7514_SAC	22110145
25_2386	ctg7180001821415_8455_SCT	22111086
25_2387	ctg7180001666715_2324_SCT	22115437
25_2388	ctg7180001666715_2079_SAC	22115682
25_2389	ctg7180001458952_850_SGT	22118925
25_2390	ctg7180001458952_2771_SAG	22120846
25_2391	ctg7180001458952_3181_SAG	22121256
25_2392	ctg7180001458952_3722_SCT	22121797
25_2393	ctg7180001810544_2167_SAC	22158905
25_2394	ctg7180001810544_777_SGT	22160295
25_2395	ctg7180001789999_4918_SAG	22164893
25_2396	ctg7180001846657_2944_SGT	22180654
25_2397	ctg7180001601521_1662_SAG	22202508
25_2398	ctg7180001601521_1647_SAC	22202523
25_2399	ctg7180001601521_902_SAG	22203268
25_2400	ctg7180001828382_2999_SAG	22207131
25_2401	ctg7180001828382_5147_SCT	22209279
25_2402	ctg7180001883264_2311_SCT	22236296
25_2403	ctg7180001554359_1549_SCT	22249539
25_2404	ctg7180001532522_325_SAC	22252751
25_2405	ctg7180001882972_1388_SCT	22256369
25_2406	ctg7180001882972_6315_SAC	22261296
25_2407	ctg7180001430142_3963_SCT	22272417
25_2408	ctg7180001848868_4040_SGT	22288658
25_2409	ctg7180001848868_5769_SGT	22290387
25_2410	ctg7180001848868_6089_SAC	22290707
25_2411	ctg7180001534138_1505_SAC	22306526

25_2412	ctg7180001534138_1815_SAG	22306836
25_2413	ctg7180001534138_4738_SAC	22309759
25_2414	ctg7180001862341_2406_SGT	22323006
25_2415	ctg7180001871617_2999_SAG	22356927
25_2416	ctg7180001871617_2889_SAC	22357037
25_2417	ctg7180001919974_11861_SCT	22361396
25_2418	ctg7180001919974_11551_SCT	22361706
25_2419	ctg7180001919974_3944_SCT	22369313
25_2420	ctg7180001799518_4930_SAG	22414260
25_2421	ctg7180001403415_1046_SAC	22442055
25_2422	ctg7180001279571_4949_SAG	22443923
25_2423	ctg7180001279571_4235_SAC	22444637
25_2424	ctg7180001794875_670_SCT	22450483
25_2425	ctg7180001794875_3739_SAC	22453552
25_2426	ctg7180001794875_8504_SCT	22458984
25_2427	ctg7180001907426_7096_SCT	22538209
25_2428	ctg7180001874605_2094_SAG	22581028
25_2429	ctg7180001874605_2485_SCT	22581419
25_2430	ctg7180001874605_2544_SGT	22581478
25_2431	ctg7180001887714_6419_SCT	22655512
25_2432	GCR_cBin5128_Ctg1_77	22662454
25_2433	ctg7180001658589_537_SCT	22668081
25_2434	ctg7180001651396_999_SCT	22690012
25_2435	ctg7180001651396_250_SAG	22690761
25_2436	ctg7180001341023_1142_SAG	22699056
25_2437	ctg7180001341023_1647_SAG	22699561
25_2438	ctg7180001341023_1654_SAG	22699568
25_2439	ctg7180001341023_1690_SCT	22699604
25_2440	ctg7180001795950_3894_SAG	22706906
25_2441	ctg7180001795950_4014_SAG	22707026
25_2442	ctg7180001844146_7455_SAC	22722256
25_2443	ctg7180001844146_4204_SCT	22725507
25_2444	ctg7180001844146_3555_SAG	22726156
25_2445	ctg7180001208382_5008_SAC	22738769
25_2446	ctg7180001829093_10842_SAG	22758578

25_2447	ctg7180001426531_2189_SAC	22765527
25_2448	ctg7180001833332_8620_SGT	22822116
25_2449	ctg7180001833332_10664_SAG	22824160
25_2450	ctg7180001833332_18340_SAG	22831836
25_2451	ctg7180001833332_37517_SAC	22851013
25_2452	ctg7180001833332_38049_SAG	22851545
25_2453	ctg7180001833332_38071_SAC	22851567
25_2454	ctg7180001833332_38125_SCT	22851621
25_2455	GCR_cBin17723_Ctg1_219	22892107
25_2456	ctg7180001875223_5124_SAC	22902107
25_2457	ctg7180001459204_2693_SAG	22910097
25_2458	ctg7180001698483_1424_SAG	22926536
25_2459	ctg7180001851856_2313_SCT	22934462
25_2460	ctg7180001793040_1508_SAC	22944558
25_2461	ctg7180001793040_9491_SCT	22952541
25_2462	ctg7180001312216_453_SAG	22957303
25_2463	ctg7180001312216_886_SAC	22957736
25_2464	ctg7180001650710_5177_SCT	22960830
25_2465	ctg7180001650710_3707_SCT	22962300
25_2466	ctg7180001893481_395_SCT	22966775
25_2467	ctg7180001820311_12112_SAG	22976962
25_2468	ctg7180001820311_11456_SCT	22977618
25_2469	ctg7180001820311_8927_SCT	22980147
25_2470	ctg7180001625439_1325_SGT	22999330
25_2471	ctg7180001625439_680_SAG	22999975
25_2472	ctg7180001605156_3979_SCT	23002668
25_2473	ctg7180001605156_3838_SCT	23002809
25_2474	ctg7180001605156_2405_SGT	23004242
25_2475	ctg7180001816210_2263_SGT	23012564
25_2476	ctg7180001836737_10900_SCT	23049299
25_2477	ctg7180001932774_13448_SAG	23083668
25_2478	ctg7180001932774_9390_SAG	23087726
25_2479	ctg7180001759736_1295_SCT	23106287
25_2480	ctg7180001759736_1730_SAC	23106757
25_2481	ctg7180001876505_22940_SAC	23125524

25_2482	ctg7180001805811_413_SAC	23150528
25_2483	ctg7180001805811_1425_SAC	23151540
25_2484	ctg7180001742697_655_SAG	23164263
25_2485	ctg7180001742697_362_SAC	23164556
25_2486	ctg7180001867079_799_SCT	23197120
25_2487	ctg7180001300731_1902_SCT	23247988
25_2488	ctg7180001646097_1239_SGT	23289323
25_2489	ctg7180001859286_1634_SGT	23345839
25_2490	ctg7180001859286_3086_SAC	23347291
25_2491	ctg7180001859286_5693_SCT	23349900
25_2492	ctg7180001905813_17431_SAC	23358102
25_2493	ctg7180001905813_12652_SAG	23362881
25_2494	ctg7180001905813_12485_SAG	23363048
25_2495	ctg7180001905813_9093_SAC	23366440
25_2496	ctg7180001905813_8162_SAG	23367371
25_2497	ctg7180001934842_9090_SCT	23391426
25_2498	ctg7180001934842_9459_SAC	23391795
25_2499	ctg7180001934842_10575_SGT	23392911
25_2500	ctg7180001934842_15799_SAG	23398135
25_2501	ctg7180001839258_6103_SCT	23436886
25_2502	ctg7180001839258_5597_SGT	23437392
25_2503	ctg7180001808171_5950_SAG	23448951
25_2504	ctg7180001497798_678_SCT	23459941
25_2505	ctg7180001799852_13978_SAG	23464043
25_2506	ctg7180001799852_11462_SGT	23466560
25_2507	ctg7180001799852_2015_SAG	23476007
25_2508	ctg7180001883966_1589_SAG	23509948
25_2509	ctg7180001883966_1952_SAC	23510311
25_2510	ctg7180001793742_3388_SCT	23517825
25_2511	ctg7180001833116_4702_SCT	23568286
25_2512	ctg7180001794170_1141_SCT	23602972
25_2513	ctg7180001621394_735_SAC	23608839
25_2514	ctg7180001386517_1153_SCT	23610949
25_2515	ctg7180001849073_6259_SGT	23617947
25_2516	ctg7180001883626_1084_SAC	23683273

25_2517	ctg7180001383086_3523_SAG	23702120
25_2518	ctg7180001326003_5594_SAC	23718499
25_2519	ctg7180001326003_5684_SGT	23718589
25_2520	ctg7180001536526_1042_SGT	23734947
25_2521	ctg7180001252143_12040_SAG	23750148
25_2522	ctg7180001252143_11473_SAG	23750715
25_2523	ctg7180001252143_10983_SCT	23751205
25_2524	ctg7180001231622_641_SGT	23772561
25_2525	ctg7180001234739_1745_SAG	23775086
25_2526	ctg7180001388351_1544_SAG	23781288
25_2527	ctg7180001807757_5110_SAG	23788724
25_2528	ctg7180001807757_10409_SAG	23794022
25_2529	ctg7180001511012_5394_SAG	23810311
25_2530	ctg7180001511012_4713_SCT	23810992
25_2531	ctg7180001602540_2770_SAC	23817731
25_2532	ctg7180001796855_1599_SAC	23823291
25_2533	ctg7180001826244_6747_SCT	23830349
25_2534	ctg7180001826244_5594_SAC	23831502
25_2535	ctg7180001937078_1888_SGT	23839356
25_2536	ctg7180001937078_3338_SAG	23840806
25_2537	ctg7180001937078_7377_SAG	23844845
25_2538	ctg7180001836010_1012_SAG	23882827
25_2539	ctg7180001836010_12023_SAG	23893838
25_2540	ctg7180001740502_888_SAG	23899124
25_2541	ctg7180001790770_2399_SAC	23931999
25_2542	ctg7180001790770_2535_SAG	23932135
25_2543	ctg7180001813366_1322_SAC	23938112
25_2544	ctg7180001401300_7464_SCT	23957442
25_2545	ctg7180001838061_6659_SAG	23985556
25_2546	ctg7180001887214_6430_SGT	24019654
25_2547	ctg7180001887214_15974_SCT	24029198
25_2548	ctg7180001887214_14895 24029359	
25_2549	ctg7180001838417_1400_SAC	24046692
25_2550	ctg7180001523165_2271_SAC	24050124
25_2551	ctg7180001509948_2655_SAC	24055993

25_2552	ctg7180001509948_1720_SAC	24056928
25_2553	ctg7180001509948_483_SAG	24058165
25_2554	ctg7180001564061_6196_SGT	24059415
25_2555	ctg7180001564061_2122_SCT	24063489
25_2556	GCR_cBin18679_Ctg1_42	24063801
25_2557	ctg7180001628350_2410_SGT	24078460
25_2558	ctg7180001832411_1600_SAG	24094492
25_2559	ctg7180001361822_1644_SGT	24116658
25_2560	ctg7180001361822_2076_SCT	24117090
25_2561	ctg7180001361822_4288_SCT	24119302
25_2562	ctg7180001361822_4331_SAG	24119345
25_2563	ctg7180001361822_6513_SCT	24121527
25_2564	ctg7180001361822_8027_SGT	24123041
25_2565	ctg7180001902122_5491_SCT	24147018
25_2566	ctg7180001902121_3231_SCT	24155159
25_2567	ctg7180001860867_4339_SAG	24166890
25_2568	ctg7180001860867_4784_SAG	24167335
25_2569	ctg7180001860867_4925_SAG	24167476
25_2570	ctg7180001804456_1656_SCT	24186421
25_2571	ctg7180001842907_3187_SAC	24193716
25_2572	ctg7180001889095_10958_SCT	24225197
25_2573	ctg7180001798138_5554_SAG	24237354
25_2574	ctg7180001827809_1562_SAC	24249965
25_2575	ctg7180001827809_1949_SAC	24250352
25_2576	ctg7180001827809_2383_SGT	24250786
25_2577	ctg7180001827809_2984_SAG	24251387
25_2578	ctg7180001846839_9532_SAG	24266454
25_2579	ctg7180001834597_7217_SCT	24319892
25_2580	ctg7180001326695_380_SCT	24356603
25_2581	ctg7180001839420_10052_SCT	24375474
25_2582	ctg7180001491983_1843_SAG	24408740
25_2583	ctg7180001900919_514_SCT	24411617
25_2584	ctg7180001376968_2425_SCT	24440421
25_2585	ctg7180001603978_4213_SCT	24444976
25_2586	ctg7180001866016_810_SGT	24447091

25_2587	ctg7180001927673_3326_SCT	24456476
25_2588	ctg7180001926086_5976_SAG	24568019
25_2589	ctg7180001807186_269_SAC	24581969
25_2590	ctg7180001795378_9150_SAG	24614143
25_2591	ctg7180001795378_8779_SGT	24614514
25_2592	ctg7180001929973_319_SGT	24624089
25_2593	ctg7180001929973_2312_SAC	24626082
25_2594	ctg7180001848015_7035_SAC	24634547
25_2595	ctg7180001848015_6680_SCT	24634902
25_2596	ctg7180001819524_230_SGT	24646871
25_2597	ctg7180001819524_2434_SGT	24649018
25_2598	ctg7180001819524_3619_SAC	24650203
25_2599	ctg7180001819524_3673_SCT	24650257
25_2600	ctg7180001804895_1480_SAG	24654430
25_2601	ctg7180001804895_1891_SAG	24654841
25_2602	ctg7180001897349_4664_SGT	24692279
25_2603	ctg7180001431420_4288_SAG	24735120
25_2604	ctg7180001908717_8886_SGT	24942051
25_2605	ctg7180001342874_1990_SCT	24954893
25_2606	ctg7180001851775_10268_SAG	24993831
25_2607	ctg7180001852750_2827_SCT	25051753
25_2608	ctg7180001852750_2857_SGT	25051783
25_2609	ctg7180001344143_1257_SAC	25152690
25_2610	ctg7180001801534_2562_SAC	25236426
25_2611	ctg7180001801534_2954_SCT	25236818
25_2612	ctg7180001823194_3283_SAG	25252067
25_2613	ctg7180001368843_2148_SAG	25263062
25_2614	ctg7180001887122_2376_SAC	25269171
25_2615	ctg7180001808600_864_SAG	25288149
25_2616	ctg7180001909190_4809_SCT	25411957
25_2617	ctg7180001909190_8462_SAG	25415610
25_2618	ctg7180001909190_10141_SGT	25417289
25_2619	ctg7180001909190_11355_SAC	25418503
25_2620	ctg7180001909190_11723_SCT	25418871
25_2621	ctg7180001810687_9623_SGT	25511043

25_2622	ctg7180001646273_1207_SAG	25525959
25_2623	ctg7180001879511_8871_SAC	25572024
25_2624	ctg7180001879511_9289_SCT	25572408
25_2625	ctg7180001879511_15632_SCT	25578751
25_2626	ctg7180001922282_11638_SAG	25587922
25_2627	ctg7180001847893_12021_SAG	25610681
25_2628	ctg7180001797659_1636_SAG	25645878
25_2629	ctg7180001797659_2360_SAG	25646602
25_2630	ctg7180001797659_4356_SGT	25648598
25_2631	ctg7180001797659_5080_SAC	25649322
25_2632	ctg7180001797659_5326_SAC	25649568
25_2633	ctg7180001892505_4274_SGT	25658483
25_2634	ctg7180001638727_1744_SGT	25665264
25_2635	ctg7180001629360_3057_SAG	25685385
25_2636	ctg7180001332540_3961_SGT	25697390
25_2637	ctg7180001881502_7331_SAG	25754715
25_2638	ctg7180001809438_604_SCT	25797455
25_2639	ctg7180001809438_3281_SGT	25800132
25_2640	ctg7180001288827_3963_SAC	25823484
25_2641	ctg7180001818863_6445_SAC	25830711
25_2642	ctg7180001818863_5906_SCT	25831250
25_2643	ctg7180001818863_5850_SAG	25831306
25_2644	ctg7180001818863_4780_SCT	25832376
25_2645	ctg7180001818863_4604_SCT	25832552
25_2646	ctg7180001909574_1332_SAG	25844611
25_2647	ctg7180001920579_17760_SCT	25903811
25_2648	ctg7180001920579_15383_SCT	25906188
25_2649	ctg7180001810860_3563_SCT	25919162
25_2650	ctg7180001810446_15256_SAC	26007254
25_2651	ctg7180001815625_5541_SAC	26010115
25_2652	ctg7180001847724_5692_SCT	26035258
25_2653	ctg7180001869377_2533_SAG	26065458
25_2654	ctg7180001915904_5521_SCT	26090974
25_2655	ctg7180001485809_5173_SGT	26105559
25_2656	ctg7180001485809_5337_SGT	26105723

25_2657	ctg7180001667431_8638_SAC	26122734
25_2658	ctg7180001750134_1191_SAC	26143498
25_2659	ctg7180001799374_9041_SCT	26153198
25_2660	ctg7180001926764_1332_SAG	26223673
25_2661	ctg7180001926764_5000_SGT	26227341
25_2662	ctg7180001926764_5084_SCT	26227425
25_2663	ctg7180001926764_7995_SCT	26230336
25_2664	ctg7180001926764_16522_SAG	26238863
25_2665	ctg7180001926764_16755_SCT	26239096
25_2666	ctg7180001926764_17932_SAC	26240273
25_2667	ctg7180001926764_25794_SCT	26248135
25_2668	ctg7180001556422_1684_SAC	26260899
25_2669	ctg7180001556422_2690_SCT	26261905
25_2670	ctg7180001556422_4022_SCT	26263237
25_2671	ctg7180001556422_4971_SCT	26264186
25_2672	ctg7180001926765_62_SAG	26275012
25_2673	ctg7180001572310_383_SAG	26298683
25_2674	ctg7180001796150_634_SGT	26338577
25_2675	ctg7180001552604_2542_SAG	26363391
25_2676	ctg7180001552604_1618_SAC	26364315
25_2677	ctg7180001843752_2846_SAC	26378978
25_2678	ctg7180001848002_1763_SAG	26401513
25_2679	ctg7180001848002_2126_SAG	26401876
25_2680	ctg7180001848002_3045_SGT	26402804
25_2681	ctg7180001909149_1310_SCT	26404476
25_2682	ctg7180001909149_2700_SCT	26405866
25_2683	ctg7180001909149_5314_SAG	26408480
25_2684	ctg7180001909149_5744_SAC	26408910
25_2685	ctg7180001909149_6270_SCG	26409436
25_2686	ctg7180001909149_10850_SGT	26414193
25_2687	ctg7180001840150_6727_SCT	26425690
25_2688	ctg7180001179486_1317_SCT	26434112
25_2689	ctg7180001740781_746_SGT	26448490
25_2690	ctg7180001740781_555_SAC	26448681
25_2691	ctg7180001371990_5192_SCT	26451132

25_2692	ctg7180001248702_3880_SCT	26497967
25_2693	ctg7180001248702_1721_SAC	26500126
25_2694	ctg7180001571548_1236_SCT	26503272
25_2695	ctg7180001909883_13063_SAG	26512184
25_2696	ctg7180001909883_11784_SGT	26513463
25_2697	ctg7180001909883_4360_SCT	26520887
25_2698	ctg7180001909883_3834_SCT	26521413
25_2699	ctg7180001909883_3422_SCT	26521825
25_2700	ctg7180001645641_4184_SAG	26534461
25_2701	ctg7180001645641_3874_SAG	26534771
25_2702	ctg7180001910271_11497_SAC	26541046
25_2703	ctg7180001910271_4553_SCT	26547990
25_2704	ctg7180001864701_272_SAG	26568573
25_2705	ctg7180001906163_14150_SCT	26599564
25_2706	ctg7180001906163_14052_SAG	26599662
25_2707	ctg7180001906163_13281_SGT	26600433
25_2708	ctg7180001906163_11354_SAG	26602360
25_2709	ctg7180001906163_6106_SGT	26607608
25_2710	ctg7180001906163_5153_SAC	26608561
25_2711	ctg7180001906163_3784_SAC	26609930
25_2712	ctg7180001814335_3074_SGT	26643301
25_2713	ctg7180001651744_5438_SGT	26654623
25_2714	ctg7180001841417_3732_SAG	26678726
25_2715	ctg7180001841417_3420_SAG	26679038
25_2716	ctg7180001863254_2710_SAC	26695129
25_2717	ctg7180001245222_328_SCT	26703084
25_2718	ctg7180001543199_1403_SAG	26708776
25_2719	ctg7180001892535_1487_SCT	26726038
25_2720	ctg7180001805422_72_SGT	26727690
25_2721	ctg7180001805422_2701_SCT	26730319
25_2722	ctg7180001759139_75_SAC	26755928
25_2723	ctg7180001238763_4003_SAG	26778862
25_2724	ctg7180001832421_1387_SAC	26833368
25_2725	ctg7180001673163_1290_SAC	26857896
25_2726	ctg7180001908182_462_SCT	26865493

25_2727	ctg7180001908182_2500_SGT	26867531
25_2728	ctg7180001908182_3287_SCT	26868318
25_2729	ctg7180001908183_22553_SAC	26893211
25_2730	ctg7180001908183_28047_SCT	26898705
25_2731	ctg7180001881155_4952_SAG	26915013
25_2732	ctg7180001881155_3712_SAC	26916253
25_2733	ctg7180001881155_3402_SGT	26916563
25_2734	ctg7180001881155_2980_SAC	26916985
25_2735	ctg7180001881155_9052_SCT	26920116
25_2736	ctg7180001858811_994_SGT	26928936
25_2737	ctg7180001529109_398_SAG	26979347
25_2738	ctg7180001793736_5914_SAC	27028965
25_2739	ctg7180001793736_5658_SGT	27029221
25_2740	ctg7180001793736_5570_SGT	27029309
25_2741	ctg7180001793736_5451_SGT	27029428
25_2742	GCR_cBin47052_Ctg1_234	27032128
25_2743	ctg7180001292259_587_SAG	27076886
25_2744	ctg7180001813894_2633_SCT	27152882
25_2745	ctg7180001725541_1818_SGT	27174373
25_2746	ctg7180001881503_15830_SCT	27204585
25_2747	ctg7180001881503_24961_SGT	27213715
25_2748	ctg7180001896200_3571_SAG	27221901
25_2749	ctg7180001896200_9297_SAC	27227627
25_2750	ctg7180001797414_7278_SAG	27325964
25_2751	ctg7180001797414_6168_SCT	27327074
25_2752	ctg7180001886067_8522_SAG	27349737
25_2753	ctg7180001677000_945_SAG	27406364
25_2754	ctg7180001872241_9060_SAG	27423764
25_2755	ctg7180001872241_9100_SGT	27423804
25_2756	ctg7180001355208_954_SAC	27427683
25_2757	ctg7180001802525_3389_SAG	27490816
25_2758	ctg7180001802525_3445_SGT	27490872
25_2759	ctg7180001802525_4387_SCT	27491814
25_2760	ctg7180001802525_4543_SAC	27491970
25_2761	ctg7180001807588_3663_SAC	27496674

25_2762	ctg7180001807588_5508_SAG	27498519
25_2763	ctg7180001807588_6113_SAC	27499124
25_2764	ctg7180001807588_7224_SAC	27500235
25_2765	ctg7180001807588_8233_SAC	27501244
25_2766	ctg7180001778678_2801_SCT	27542432
25_2767	ctg7180001720772_483_SAC	27549801
25_2768	ctg7180001817064_13105_SAC	27567664
25_2769	ctg7180001817064_12034_SAG	27568735
25_2770	ctg7180001817064_3865_SAG	27576904
25_2771	ctg7180001915338_4230_SAC	27582050
25_2772	ctg7180001915338_3917_SCT	27582363
25_2773	ctg7180001682865_3897_SGT	27589118
25_2774	ctg7180001682865_2723_SAG	27590292
25_2775	ctg7180001682865_844_SAG	27592171
25_2776	ctg7180001682865_539_SGT	27592476
25_2777	ctg7180001895961_1220_SAG	27594608
25_2778	ctg7180001895961_1823_SGT	27595211
25_2779	ctg7180001895961_7438_SAG	27600826
25_2780	ctg7180001895961_18190_SAG	27611578
25_2781	ctg7180001895961_23865_SAG	27617253
25_2782	ctg7180001895961_23990_SGT	27617378
25_2783	ctg7180001299944_220_SGT	27722326
25_2784	ctg7180001892186_3744_SAG	27738756
25_2785	ctg7180001892186_7016_SCT	27742028
25_2786	ctg7180001890747_5388_SGT	27749268
25_2787	ctg7180001616982_866_SAG	27755437
25_2788	ctg7180001616982_3976 27758582	
25_2789	ctg7180001711354_1240_SAG	27782296
25_2790	ctg7180001711354_2064_SGT	27783120
25_2791	ctg7180001850807_4763_SCT	27803001
25_2792	ctg7180001812413_3913_SGT	27810215
25_2793	ctg7180001829588_2800_SAC	27814838
25_2794	ctg7180001829588_1980_SGT	27815658
25_2795	ctg7180001898325_3997_SGT	27831650
25_2796	ctg7180001898325_3546_SAG	27832101

25_2797	ctg7180001804408_2270_SAG	27857394
25_2798	ctg7180001659224_5777_SGT	27861558
25_2799	ctg7180001659224_2633_SGT	27864702
25_2800	ctg7180001659224_2623_SCT	27864712
25_2801	ctg7180001854008_612_SGT	27873001
25_2802	ctg7180001854008_11471_SAG	27883859
25_2803	ctg7180001854008_11656_SAC	27884044
25_2804	ctg7180001854008_11986_SGT	27884374
25_2805	ctg7180001810525_4823_SAG	27894985
25_2806	ctg7180001810525_4303_SGT	27895505
25_2807	ctg7180001810525_4252_SAG	27895556
25_2808	ctg7180001810525_3429_SAG	27896379
25_2809	ctg7180001936957_1093_SAG	27920979
25_2810	ctg7180001936957_11464_SAG	27931350
25_2811	ctg7180001837052_6107_SAC	27938312
25_2812	ctg7180001837052_6186_SGT	27938391
25_2813	ctg7180001805906_271_SGT	27941180
25_2814	ctg7180001739004_612_SAG	27950589
25_2815	ctg7180001884023_13858_SAG	27965662
25_2816	ctg7180001884023_15006_SGT	27966810
25_2817	ctg7180001347888_38_SAG	27995264
25_2818	ctg7180001803568_9462_SCT	27996451
25_2819	ctg7180001803568_9420_SAG	27996493
25_2820	ctg7180001734714_331_SCT	28027109
25_2821	ctg7180001798042_1011_SAG	28028647
25_2822	ctg7180001355291_1188_SAC	28038772
25_2823	ctg7180001880165_23989_SAG	28040975
25_2824	ctg7180001880165_23979_SAG	28040985
25_2825	ctg7180001880165_23719_SAG	28041245
25_2826	ctg7180001880165_23032_SGT	28041932
25_2827	ctg7180001880165_19433_SGT	28045531
25_2828	ctg7180001880165_12731_SCT	28052233
25_2829	ctg7180001880165_5795_SAC	28059169
25_2830	ctg7180001880165_3669_SGT	28061295
25_2831	ctg7180001880165_1540_SAG	28063424

25_2832	ctg7180001310771_6085_SAG	28070904
25_2833	ctg7180001310771_4053_SGT	28072936
25_2834	ctg7180001930952_1880_SGT	28092242
25_2835	ctg7180001884987_2250_SCT	28113549
25_2836	ctg7180001318378_2081_SAG	28118559
25_2837	ctg7180001820033_94_SAG	28213026
25_2838	ctg7180001890139_977_SAC	28250639
25_2839	ctg7180001858643_2408_SGT	28286301
25_2840	ctg7180001858643_3159_SCT	28287053
25_2841	ctg7180001744667_582_SAG	28292829
25_2842	ctg7180001726697_1384_SAC	28329580
25_2843	ctg7180001542157_896_SGT	28342845
25_2844	ctg7180001542157_1539_SAC	28343488
25_2845	ctg7180001203108_914_SAG	28347797
25_2846	ctg7180001920240_1767_SGT	28372407
25_2847	ctg7180001934610_16099_SCT	28434156
25_2848	ctg7180001250661_1339_SGT	28450187
25_2849	ctg7180001250661_954_SAC	28450573
25_2850	ctg7180001583344_596_SCT	28509665
25_2851	ctg7180001486290_2233_SCT	28530559
25_2852	ctg7180001486290_6549_SCT	28534875
25_2853	ctg7180001531535_539_SGT	28540775
25_2854	ctg7180001793658_11980_SCT	28560742
25_2855	ctg7180001814248_9914_SAC	28576661
25_2856	ctg7180001814248_10634_SAC	28577381
25_2857	ctg7180001794462_640_SAG	28596657
25_2858	ctg7180001220062_4416_SAC	28615893
25_2859	ctg7180001811241_779_SAG	28621093
25_2860	ctg7180001850052_9238_SAG	28653832
25_2861	ctg7180001850052_1764_SAC	28654448
25_2862	ctg7180001252965_260_SAC	28663684
25_2863	ctg7180001252965_684_SAC	28664108
25_2864	ctg7180001252965_713_SAG	28664137
25_2865	ctg7180001252965_1441_SAC	28664865
25_2866	ctg7180001203353_845_SGT	28700414

25_2867	ctg7180001203353_446_SAC	28700813
25_2868	ctg7180001670768_361_SAC	28701586
25_2869	ctg7180001670768_1323_SAG	28702548
25_2870	ctg7180001864496_5497_SAG	28718746
25_2871	ctg7180001803002_2834_SCT	28729553
25_2872	ctg7180001803002_5554_SAG	28732273
25_2873	ctg7180001803002_8562_SCT	28735281
25_2874	ctg7180001803002_9832_SAG	28736551
25_2875	ctg7180001615289_1171_SAG	28764051
25_2876	ctg7180001615289_5952_SAG	28768832
25_2877	ctg7180001890621_2618_SAG	28773411
25_2878	ctg7180001666522_817_SCT	28818010
25_2879	ctg7180001293477_2088_SAG	28846264
25_2880	ctg7180001217206_1077_SCT	28907969
25_2881	ctg7180001530469_5173_SGT	28932316
25_2882	ctg7180001530469_4083_SAG	28933406
25_2883	ctg7180001933657_1850_SGT	28957398
25_2884	ctg7180001598061_1328_SAC	28990635
25_2885	ctg7180001598061_1544_SAG	28990851
25_2886	ctg7180001598061_2099_SGT	28991582
25_2887	ctg7180001598061_2370_SAG	28991856
25_2888	ctg7180001598061_3248_SGT	28992731
25_2889	ctg7180001856106_82_SAG	29002599
25_2890	ctg7180001302654_903_SGT	29014598
25_2891	ctg7180001244069_1355_SAG	29027850
25_2892	ctg7180001290321_1250_SCT	29064200
25_2893	ctg7180001824172_39_SAG	29065676
25_2894	ctg7180001824172_1209_SCT	29066846
25_2895	ctg7180001824172_6445_SCT	29072084
25_2896	ctg7180001824172_9302_SAG	29074941
25_2897	ctg7180001824172_11405_SAG	29077044
25_2898	ctg7180001824172_11430_SAC	29077069
25_2899	ctg7180001824172_14303_SGT	29079942
25_2900	ctg7180001824172_15048_SAG	29080687
25_2901	ctg7180001262024_1217_SAC	29086467

25_2902	ctg7180001897260_6908_SGT	29262924
25_2903	ctg7180001897260_4875_SGT	29264957
25_2904	ctg7180001897260_4837_SCT	29264995
25_2905	ctg7180001878766_3030_SCT	29280416
25_2906	ctg7180001878766_16713_SAG	29280485
25_2907	ctg7180001878766_10495_SAC	29286703
25_2908	ctg7180001878766_7706_SAG	29289318
25_2909	ctg7180001878766_7677_SGT	29289347
25_2910	ctg7180001878766_6197_SAG	29290827
25_2911	ctg7180001878766_3023_SCT	29294001
25_2912	ctg7180001878766_64_SAG	29296960
25_2913	ctg7180001928907_3979_SGT	29320669
25_2914	ctg7180001850326_3551_SCT	29331467
25_2915	ctg7180001867907_971_SAG	29342221
25_2916	ctg7180001318755_3540_SAG	29363276
25_2917	ctg7180001912739_11460_SAG	29371511
25_2918	ctg7180001912739_4845_SGT	29378126
25_2919	ctg7180001897261_4133_SAG	29449426
25_2920	ctg7180001897260_11690_SCT	29465290
25_2921	ctg7180001912739_973_SCT	29533194
25_2922	ctg7180001461624_781_SAC	29542902
25_2923	ctg7180001382838_1556_SAG	29562175
25_2924	ctg7180001866658_2239_SAG	29565256
25_2925	ctg7180001672640_171_SGT	29573435
25_2926	ctg7180001398019_426_SCT	29582983
25_2927	ctg7180001398019_581_SCT	29583138
25_2928	ctg7180001398019_989_SAG	29583546
25_2929	ctg7180001454085_593_SAC	29584493
25_2930	ctg7180001836683_2507_SAC	29638741
25_2931	ctg7180001209113_3557_SAC	29656054
25_2932	ctg7180001865551_2323_SAG	29681138
25_2933	ctg7180001845249_1659_SAC	29727703
25_2934	ctg7180001845249_1554_SCT	29727808
25_2935	ctg7180001845249_1137_SAG	29728225
25_2936	ctg7180001216953_316_SGT	29729971

25_2937	ctg7180001802915_4814_SCT	29768019
25_2938	ctg7180001802915_4775_SCT	29768058
25_2939	ctg7180001917706_9062_SAC	29802562
25_2940	ctg7180001917706_14380_SCT	29807880
25_2941	ctg7180001917706_14401_SCT	29807901
25_2942	ctg7180001330474_865_SGT	29814320
25_2943	ctg7180001330474_677_SCT	29814508
25_2944	ctg7180001644326_6134_SGT	29825686
25_2945	ctg7180001908924_3468_SAC	29837192
25_2946	ctg7180001692110_3411_SAG	29856728
25_2947	ctg7180001692110_4035_SAG	29857352
25_2948	ctg7180001582221_149_SCT	29862664
25_2949	ctg7180001582221_154_SCT	29862669
25_2950	ctg7180001582221_2315_SCT	29864830
25_2951	ctg7180001582221_5031_SCT	29867546
25_2952	ctg7180001315442_979_SAC	29869279
25_2953	ctg7180001870117_1584_SAG	29903855
25_2954	ctg7180001870117_2029_SAG	29904300
25_2955	ctg7180001870117_2231_SAG	29904502
25_2956	ctg7180001234393_221_SAC	29918754
25_2957	ctg7180001234393_440_SCT	29919008
25_2958	ctg7180001693420_986_SCT	29927097
25_2959	ctg7180001693420_1418_SCT	29927529
25_2960	ctg7180001807319_7852_SAC	29965133
25_2961	ctg7180001807319_8421_SAC	29965702
25_2962	ctg7180001818793_1837_SAG	29988930
25_2963	ctg7180001188116_941_SAC	30008231
25_2964	ctg7180001464935_878_SGT	30064528
25_2965	ctg7180001253623_1164_SCT	30068923
25_2966	ctg7180001864314_2792_SAG	30086971
25_2967	ctg7180001822007_6977_SAC	30169092
25_2968	ctg7180001822007_13358_SAC	30175393
25_2969	ctg7180001822007_13624_SCT	30175659
25_2970	ctg7180001681060_902_SAG	30187943
25_2971	ctg7180001934474_2121_SCT	30229233

25_2972	ctg7180001239531_859_SGT	30335953
25_2973	ctg7180001821583_23119_SAC	30401143
25_2974	ctg7180001821583_13353_SAG	30410909
25_2975	ctg7180001821583_6282_SAG	30417980
25_2976	ctg7180001821583_6232_SAG	30418030
25_2977	ctg7180001808371_7464_SCT	30430661
25_2978	ctg7180001880994_5033_SAG	30476638
25_2979	ctg7180001880994_5886_SCT	30477491
25_2980	ctg7180001920420_1660_SAC	30527509
25_2981	ctg7180001920420_1843_SCT	30527692
25_2982	ctg7180001920420_3854_SGT	30529703
25_2983	ctg7180001822359_4933_SAG	30547796
25_2984	ctg7180001854820_2321_SAG	30606194
25_2985	ctg7180001820500_7963_SAC	30622992
25_2986	ctg7180001474541_359_SAG	30636448
25_2987	ctg7180001640803_4260_SAC	30655844
25_2988	ctg7180001640803_1702_SCT	30658402
25_2989	ctg7180001188067_3351_SAG	30665200
25_2990	ctg7180001787598_1259_SAC	30673005
25_2991	ctg7180001787598_5028_SGT	30676774
25_2992	ctg7180001787598_6250_SAC	30677996
25_2993	ctg7180001787598_13490_SAC	30685236
25_2994	ctg7180001787598_13920_SAG	30685666
25_2995	ctg7180001787598_14144_SAG	30685890
25_2996	ctg7180001847831_4122_SAC	30693397
25_2997	ctg7180001888865_4372_SGT	30703186
25_2998	ctg7180001888865_5730_SAC	30704544
25_2999	ctg7180001888865_5755_SGT	30704569
25_3000	ctg7180001888865_6345_SAC	30705159
25_3001	ctg7180001794835_3991_SAC	30730961
25_3002	ctg7180001794835_4242_SCT	30731212
25_3003	ctg7180001794835_4356_SCT	30731326
25_3004	ctg7180001794835_7755_SCT	30734725
25_3005	ctg7180001809944_5435_SAG	30744692
25_3006	ctg7180001910103_7836_SAC	30757256

25_3007	ctg7180001903518_7316_SAG	30773063
25_3008	ctg7180001903518_12733_SAC	30778480
25_3009	ctg7180001903518_20315_SGT	30786062
25_3010	ctg7180001903518_22122_SAC	30787869
25_3011	ctg7180001857757_998_SAC	30820728
25_3012	ctg7180001796120_1899	30836678
25_3013	ctg7180001796120_18104_SAG	30841972
25_3014	ctg7180001796120_3316_SAC	30856760
25_3015	ctg7180001796120_2902_SCT	30857174
25_3016	ctg7180001796120_426_SAC	30859650
25_3017	ctg7180001250872_2298_SCT	30863673
25_3018	ctg7180001825589_4249_SAG	30892182
25_3019	ctg7180001793469_18317_SAG	30974121
25_3020	ctg7180001793469_18194_SCT	30974244
25_3021	ctg7180001793469_16187_SAG	30976251
25_3022	ctg7180001793469_13086_SGT	30979352
25_3023	ctg7180001793469_13080_SAG	30979358
25_3024	ctg7180001793469_5889_SAG	30986549
25_3025	ctg7180001819488_6968_SAG	31003051
25_3026	ctg7180001819488_7105_SAC	31003188
25_3027	ctg7180001819488_10387_SCT	31006470
25_3028	ctg7180001532820_439_SGT	31009122
25_3029	ctg7180001222260_5699_SAC	31017140
25_3030	ctg7180001356832_397_SCT	31018315
25_3031	ctg7180001810039_9643_SAG	31030551
25_3032	ctg7180001907873_21668_SCT	31033299
25_3033	ctg7180001907873_16812_SCT	31038155
25_3034	ctg7180001907873_16695_SCT	31038272
25_3035	ctg7180001930608_21460_SAG	31072404
25_3036	ctg7180001218565_1058_SGT	31101923
25_3037	ctg7180001674656_106_SCT	31103681
25_3038	ctg7180001674656_1232_SGT	31104350
25_3039	ctg7180001845433_1994_SCT	31107713
25_3040	ctg7180001758036_989_SGT	31140493
25_3041	ctg7180001881120_4108_SCT	31154347

25_3042	ctg7180001900213_1150_SAC	31200584
25_3043	ctg7180001550080_527_SGT	31217273
25_3044	ctg7180001469039_1206_SAG	31219402
25_3045	ctg7180001706295_6195_SCT	31242161
25_3046	ctg7180001450249_2064_SAC	31288044
25_3047	ctg7180001864724_23471_SAC	31294987
25_3048	ctg7180001864724_23044_SCT	31295414
25_3049	ctg7180001864724_22766_SAC	31295692
25_3050	ctg7180001864724_18036_SAG	31299930
25_3051	ctg7180001284260_3410_SAG	31323750
25_3052	ctg7180001366305_426_SAC	31328084
25_3053	ctg7180001366305_453_SAG	31328111
25_3054	ctg7180001206932_5119_SCT	31337107
25_3055	ctg7180001933217_793_SAG	31360827
25_3056	ctg7180001473356_6167_SCT	31394327
25_3057	ctg7180001284983_1329_SAG	31397973
25_3058	ctg7180001851551_2190_SAG	31404191
25_3059	ctg7180001873737_5462_SCT	31414929
25_3060	ctg7180001568276_1979_SAG	31463004
25_3061	ctg7180001712263_1256_SAG	31473813
25_3062	ctg7180001835968_12285_SCT	31489084
25_3063	ctg7180001732846_971_SCT	31512399
25_3064	ctg7180001855505_2421_SGT	31519712
25_3065	ctg7180001855504_8387_SAG	31524108
25_3066	ctg7180001855504_7857_SAG	31524638
25_3067	ctg7180001855504_7775_SAG	31524720
25_3068	ctg7180001855504_2065_SAG	31530430
25_3069	ctg7180001440821_354_SCT	31548232
25_3070	ctg7180001873209_5951_SGT	31568821
25_3071	ctg7180001832172_5110_SGT	31580310
25_3072	ctg7180001832172_5429_SAG	31580591
25_3073	ctg7180001202253_310_SCT	31636951
25_3074	ctg7180001535055_2196_SAG	31639571
25_3075	ctg7180001535055_6818_SCT	31644193
25_3076	ctg7180001814690_2905_SGT	31662031

25_3077	ctg7180001814690_1468_SAC	31663468
25_3078	ctg7180001814690_1417_SAC	31663519
25_3079	ctg7180001455661_1257_SAG	31666678
25_3080	ctg7180001455661_1731_SAG	31667152
25_3081	ctg7180001867595_6553_SAC	31688505
25_3082	ctg7180001297570_5662_SGT	31694374
25_3083	ctg7180001297570_5118_SGT	31694918
25_3084	ctg7180001297570_4448_SCT	31695588
25_3085	ctg7180001687140_4460_SAG	31715411
25_3086	ctg7180001895160_82_SAC	31726004
25_3087	ctg7180001920906_2968_SAG	31745840
25_3088	ctg7180001924897_13573_SCT	31776815
25_3089	ctg7180001930595_4244_SAG	31810955
25_3090	ctg7180001925718_5517_SAC	31835804
25_3091	ctg7180001894543_427_SGT	31841458
25_3092	ctg7180001894543_641_SAC	31841672
25_3093	ctg7180001894543_1471_SGT	31842501
25_3094	ctg7180001935817_636_SGT	31871802
25_3095	ctg7180001935817_4926_SCT	31876092
25_3096	ctg7180001554597_528_SGT	31907778
25_3097	ctg7180001797056_401_SAG	31924850
25_3098	ctg7180001324517_1256_SCT	31980156
25_3099	ctg7180001864784_1232_SCT	31988929
25_3100	ctg7180001864784_3801_SAG	31991533
25_3101	ctg7180001864784_4906_SAG	31992603
25_3102	ctg7180001856387_7909_SGT	32010907
25_3103	GCR_cBin6868_Ctg1_157	32041804
25_3104	ctg7180001363169_185_SAG	32073454
25_3105	ctg7180001621626_175_SAG	32136343
25_3106	ctg7180001798961_2910_SCT	32156414
25_3107	ctg7180001821405_2974_SCT	32242252
25_3108	ctg7180001248768_13681_SGT	32266869
25_3109	ctg7180001870040_5138_SGT	32305013
25_3110	ctg7180001870040_6219_SCT	32306094
25_3111	ctg7180001870040_8892_SAC	32308767

25_3112	ctg7180001267213_2822_SAG	32316665
25_3113	ctg7180001852059_5165_SAG	32323895
25_3114	ctg7180001852059_7123_SCT	32325853
25_3115	ctg7180001852059_18722_SCT	32337450
25_3116	ctg7180001291594_8405_SCT	32352882
25_3117	ctg7180001291594_10255_SCT	32354732
25_3118	ctg7180001291594_10383_SGT	32354860
25_3119	ctg7180001734517_847_SCT	32362494
25_3120	ctg7180001674143_3046_SAG	32367191
25_3121	ctg7180001822641_7324_SAG	32377444
25_3122	ctg7180001822641_6991_SGT	32377777
25_3123	ctg7180001822641_6767_SAG	32378001
25_3124	ctg7180001846648_4396_SCT	32394899
25_3125	ctg7180001591013_558_SCT	32412774
25_3126	ctg7180001591013_1006_SCT	32413222
25_3127	ctg7180001591013_3058_SCT	32415274
25_3128	ctg7180001591013_3129_SCT	32415345
25_3129	ctg7180001591013_3482_SAC	32415698
25_3130	ctg7180001591013_4779_SGT	32416995
25_3131	ctg7180001912785_9151_SGT	32430695
25_3132	ctg7180001833998_2955_SCT	32455194
25_3133	GCR_cBin19498_Ctg1_386	32455279
25_3134	ctg7180001833998_3647_SCT	32455886
25_3135	ctg7180001833998_3662_SGT	32455901
25_3136	ctg7180001833998_4676_SAG	32456915
25_3137	ctg7180001833998_8380_SCT	32460619
25_3138	ctg7180001700374_2288_SAG	32467087
25_3139	ctg7180001467679_1404_SAG	32485332
25_3140	ctg7180001764529_420_SCT	32506208
25_3141	ctg7180001764529_973_SCT	32506761
25_3142	ctg7180001243965_1793_SGT	32539846
25_3143	ctg7180001898673_3011_SAC	32610836
25_3144	ctg7180001332357_534_SGT	32626052
25_3145	ctg7180001602686_775_SAG	32670512
25_3146	ctg7180001907984_3797_SAG	32740562

25_3147	ctg7180001907984_1576_SAG	32742783
25_3148	ctg7180001265063_4186_SAG	32756024
25_3149	ctg7180001233918_2980_SGT	32767884
25_3150	ctg7180001233918_1788_SAG	32769076
25_3151	ctg7180001920407_7504_SAC	32773199
25_3152	ctg7180001920407_5688_SAC	32774793
25_3153	ctg7180001246949_2200_SAG	32843888
25_3154	ctg7180001870135_4457_SAG	32846575
25_3155	ctg7180001870135_4153_SCT	32846879
25_3156	ctg7180001483421_2193_SCT	32871023
25_3157	ctg7180001528125_453_SGT	32874022
25_3158	ctg7180001842975_12262_SAC	32919784
25_3159	ctg7180001842975_11184_SCT	32920281
25_3160	ctg7180001842975_2529_SAG	32928936
25_3161	ctg7180001819701_5646_SAC	32932773
25_3162	ctg7180001819701_3950_SCT	32934467
25_3163	ctg7180001819701_3890_SCT	32934527
25_3164	ctg7180001819701_3320_SCT	32935097
25_3165	ctg7180001819701_3274_SAG	32935143
25_3166	ctg7180001669479_3572_SAG	32942015
25_3167	ctg7180001669479_2310_SCT	32943277
25_3168	ctg7180001817619_1901_SAC	32961845
25_3169	ctg7180001928497_2262_SAC	32983986
25_3170	ctg7180001799909_4277_SAG	32997943
25_3171	ctg7180001806381_4507_SCT	33006940
25_3172	ctg7180001806381_4998_SGT	33007431
25_3173	ctg7180001806381_5492_SCG	33007925
25_3174	ctg7180001806381_5605_SAC	33008038
25_3175	ctg7180001806381_6679_SGT	33009112
25_3176	ctg7180001806381_7084_SAG	33009517
25_3177	ctg7180001806381_7201_SCT	33009634
25_3178	ctg7180001806381_8342_SAC	33010775
25_3179	ctg7180001806381_8480_SAG	33010913
25_3180	ctg7180001651443_74_SGT	33028036
25_3181	ctg7180001620438_14440_SAG	33037897

25_3182	ctg7180001620438_14271_SAG	33038066
25_3183	ctg7180001620438_7885_SCT	33044451
25_3184	ctg7180001812132_2218_SCT	33072515
25_3185	ctg7180001819404_3678_SCT	33084163
25_3186	ctg7180001871769_1650_SCT	33125431
25_3187	ctg7180001925758_4175_SCT	33181842
25_3188	ctg7180001557598_10692_SCT	33189200
25_3189	ctg7180001557598_4374_SGT	33195518
25_3190	ctg7180001557598_3625_SAG	33196267
25_3191	ctg7180001557598_3542_SAG	33196350
25_3192	ctg7180001557598_10431	33199084
25_3193	ctg7180001869671_8558_SAG	33223462
25_3194	ctg7180001869671_4273_SGT	33227798
25_3195	ctg7180001869671_3245_SAC	33228826
25_3196	ctg7180001869671_3148_SAC	33228923
25_3197	ctg7180001743389_1159_SAG	33243925
25_3198	ctg7180001458542_726_SGT	33265403
25_3199	ctg7180001458542_2466_SAC	33267143
25_3200	ctg7180001592017_482_SCT	33288134
25_3201	ctg7180001894174_6017_SGT	33319444
25_3202	ctg7180001812300_4924_SAC	33328955
25_3203	ctg7180001753439_3487_SCT	33337827
25_3204	ctg7180001826602_9447_SAG	33387033
25_3205	ctg7180001826602_9631_SCT	33387217
25_3206	ctg7180001826602_11089_SCT	33388675
25_3207	ctg7180001826602_12859_SAG	33390445
25_3208	ctg7180001810632_934_SAG	33465856
25_3209	ctg7180001849173_3997_SCT	33470283
25_3210	ctg7180001849173_3966_SAG	33470314
25_3211	ctg7180001849173_3891_SGT	33470389
25_3212	ctg7180001849173_2524_SGT	33471756
25_3213	ctg7180001798421_12294_SGT	33480385
25_3214	ctg7180001798421_12206_SCT	33480473
25_3215	ctg7180001798421_6585_SAG	33486094
25_3216	ctg7180001518282_65_SAG	33516306

25_3217	ctg7180001826346_9514_SAG	33518674
25_3218	ctg7180001826346_495_SAG	33518811
25_3219	ctg7180001826346_1217_SGT	33526971
25_3220	ctg7180001857598_3494_SAG	33540549
25_3221	ctg7180001848631_1468_SCG	33544383
25_3222	ctg7180001837274_2716_SAG	33582357
25_3223	ctg7180001858026_12856_SCT	33594801
25_3224	ctg7180001858026_6623_SAG	33601034
25_3225	GCR_cBin26296_Ctg1_29	33607227
25_3226	ctg7180001843146_1701	33620773
25_3227	ctg7180001936873_3630_SAG	33729208
25_3228	ctg7180001918356_5540_SAC	33811705
25_3229	ctg7180001883697_10553_SAC	33877163
25_3230	ctg7180001897895_5470_SGT	33901338
25_3231	ctg7180001897895_252_SCT	33907838
25_3232	ctg7180001911769_3849_SGT	33947072
25_3233	ctg7180001800060_13983_SCT	33982647
25_3234	ctg7180001798368_5287_SAG	34027620
25_3235	ctg7180001806662_4169_SAT	34038338
25_3236	ctg7180001829397_632_SAC	34043968
25_3237	ctg7180001917367_4246_SAG	34064949
25_3238	ctg7180001917367_4203_SGT	34064992
25_3239	ctg7180001917367_3229_SAG	34065966
25_3240	ctg7180001917367_2961_SGT	34066234
25_3241	ctg7180001825712_4229_SAG	34073790
25_3242	ctg7180001825712_9431_SCT	34078992
25_3243	ctg7180001825712_22802_SCT	34092363
25_3244	ctg7180001825712_24943_SAC	34094504
25_3245	ctg7180001697421_1479_SGT	34108800
25_3246	ctg7180001697421_751_SAG	34109528
25_3247	ctg7180001895945_1185_SAG	34129027
25_3248	ctg7180001446879_1992_SAC	34146394
25_3249	ctg7180001446879_1451_SGT	34146935
25_3250	ctg7180001682841_2977_SCT	34234390
25_3251	ctg7180001842668_3905_SAG	34252576

25_3252	ctg7180001905078_9228_SAG	34280169
25_3253	ctg7180001905078_12061_SAG	34283002
25_3254	ctg7180001796351_106_SAG	34309677
25_3255	ctg7180001871390_5585_SAG	34334659
25_3256	ctg7180001835062_1081_SCT	34360452
25_3257	ctg7180001271576_1586_SAG	34364814
25_3258	ctg7180001514338_2289_SGT	34394023
25_3259	ctg7180001928849_960_SGT	34403807
25_3260	ctg7180001440741_3495_SCT	34405814
25_3261	ctg7180001815861_2328_SAG	34420409
25_3262	ctg7180001792972_7815_SCT	34432503
25_3263	ctg7180001792972_11387_SAG	34436075
25_3264	ctg7180001792972_13794_SAC	34438482
25_3265	ctg7180001895759_5407_SCT	34462413
25_3266	ctg7180001895759_5446_SGT	34462452
25_3267	ctg7180001195115_336_SGT	34476866
25_3268	ctg7180001195115_140_SCT	34477062
25_3269	ctg7180001819610_1037_SCT	34480533
25_3270	ctg7180001819610_1092_SCT	34480588
25_3271	ctg7180001820342_11103_SGT	34503304
25_3272	ctg7180001860624_345_SAG	34543256
25_3273	ctg7180001603393_1400_SCT	34605894
25_3274	ctg7180001880823_384_SAG	34617061
25_3275	ctg7180001906056_111_SCT	34716290
25_3276	ctg7180001906054_6895_SAG	34733895
25_3277	ctg7180001854470_3239_SGT	34743750
25_3278	ctg7180001798478_3205_SGT	34890271
25_3279	ctg7180001855610_3963_SAC	34901107
25_3280	ctg7180001855610_6778_SAG	34903922
25_3281	ctg7180001553822_361_SCT	34909576
25_3282	ctg7180001553822_737_SGT	34909952
25_3283	ctg7180001553822_849_SGT	34910064
25_3284	ctg7180001469611_68_SAC	35011018
25_3285	ctg7180001480439_1057_SAG	35015309
25_3286	ctg7180001446145_1118_SGT	35024620

25_3287	ctg7180001821014_1580_SCT	35049116
25_3288	ctg7180001821014_1394_SGT	35049302
25_3289	ctg7180001573080_1930_SAG	35055731
25_3290	ctg7180001474425_1255_SAG	35090997
25_3291	ctg7180001474425_1327_SAC	35091034
25_3292	ctg7180001474425_1731_SCT	35091438
25_3293	ctg7180001666935_647_SAG	35116630
25_3294	ctg7180001820518_7450_SAG	35156938
25_3295	ctg7180001820518_3027_SCT	35161361
25_3296	ctg7180001820518_1545_SCT	35162843
25_3297	ctg7180001290019_1071_SCT	35255524
25_3298	ctg7180001290019_3316_SGT	35257769
25_3299	ctg7180001825547_2673_SGT	35306795
25_3300	ctg7180001572779_431_SAG	35332638
25_3301	ctg7180001932625_965_SCT	35334947
25_3302	ctg7180001886705_9693_SCT	35351743
25_3303	ctg7180001725696_1197_SAG	35378955
25_3304	ctg7180001497252_329_SCT	35381055
25_3305	ctg7180001815958_8569_SAG	35394622
25_3306	ctg7180001815958_1167_SAC	35404984
25_3307	ctg7180001815958_626_SCT	35405525
25_3308	ctg7180001815958_98_SCT	35406053
25_3309	ctg7180001900828_2672_SAC	35409725
25_3310	ctg7180001803075_1535_SAC	35441935
25_3311	ctg7180001857680_5268_SAG	35476535
25_3312	ctg7180001614018_5633_SCT	35544134
25_3313	ctg7180001253261_13703_SAG	35560184
25_3314	ctg7180001887854_15788_SAG	35591622
25_3315	ctg7180001887854_15708_SAG	35591702
25_3316	ctg7180001887854_15453_SAG	35591957
25_3317	ctg7180001887854_14925_SAG	35592485
25_3318	ctg7180001887854_11736_SAG	35595674
25_3319	ctg7180001887853_6538_SAC	35608431
25_3320	ctg7180001682858_932_SAG	35615983
25_3321	ctg7180001682858_1027_SAC	35616078

25_3322	ctg7180001808019_4084_SAC	35625619
25_3323	ctg7180001808019_4556_SGT	35626091
25_3324	ctg7180001808019_4802_SGT	35626337
25_3325	ctg7180001808019_8231_SAG	35629766
25_3326	ctg7180001808019_11817_SAG	35633352
25_3327	ctg7180001808019_11911_SAC	35633446
25_3328	ctg7180001808019_12194_SGT	35633729
25_3329	ctg7180001808019_17061_SGT	35638596
25_3330	ctg7180001663464_3981_SCT	35646335
25_3331	ctg7180001817497_3157_SCT	35671527
25_3332	ctg7180001338613_504_SAG	35697013
25_3333	ctg7180001822705_2298_SAG	35702146
25_3334	ctg7180001559379_1894_SAG	35717450
25_3335	ctg7180001208791_3624_SAG	35723975
25_3336	ctg7180001208791_4888_SCT	35725239
25_3337	ctg7180001365528_584_SCT	35748216
25_3338	ctg7180001864821_2983	35786944
25_3339	ctg7180001864821_14937_SAG	35798864
25_3340	ESTNV_29854_323	35831634
25_3341	ctg7180001934334_7741_SAC	35844773
25_3342	ctg7180001872619_4808_SCT	35863935
25_3343	ctg7180001937288_17023_SAC	35881515
25_3344	ctg7180001816502_3612_SAG	35890858
25_3345	ctg7180001332636_1889_SGT	35911981
25_3346	ctg7180001332636_2691_SAC	35912783
25_3347	ctg7180001332636_4818_SCT	35914910
25_3348	ctg7180001516401_414_SAG	35916252
25_3349	ctg7180001891828_1754_SGT	35952429
25_3350	ctg7180001931701_2438_SAC	35974411
25_3351	ctg7180001931701_2655_SAG	35974628
25_3352	ctg7180001931701_2850_SAG	35974823
25_3353	ctg7180001897479_4396_SCT	35986204
25_3354	ctg7180001930856_215_SCT	36012305
25_3355	ctg7180001902356_4052_SAG	36014836
25_3356	ctg7180001904926_4123_SAG	36040571

25_3357	ctg7180001904926_5253_SCT	36041701
25_3358	ctg7180001904926_9625_SAG	36046073
25_3359	ctg7180001904926_9773_SCT	36046221
25_3360	ctg7180001904926_10804_SCT	36047252
25_3361	ctg7180001904926_12451_SCT	36048899
25_3362	ctg7180001584091_868_SGT	36061344
25_3363	ctg7180001902761_6083_SAG	36069057
25_3364	ctg7180001902761_5333_SAC	36069807
25_3365	ctg7180001793180_11163_SAC	36076383
25_3366	ctg7180001793180_11085_SCT	36076461
25_3367	ctg7180001793180_8400_SAG	36079146
25_3368	ctg7180001793180_3785_SCT	36083467
25_3369	ctg7180001873070_9956_SAG	36088229
25_3370	ctg7180001873070_9410_SCT	36088775
25_3371	ctg7180001873070_8381_SAC	36089804
25_3372	ctg7180001873070_8357_SAC	36089828
25_3373	ctg7180001873070_1312_SAG	36096873
25_3374	ctg7180001201619_346_SCT	36101797
25_3375	ctg7180001247823_4760_SAG	36107849
25_3376	ctg7180001549079_1450_SAG	36123413
25_3377	ctg7180001909657_4919_SAG	36137488
25_3378	ctg7180001909657_418_SAG	36141990
25_3379	ctg7180001899531_11381_SCT	36198897
25_3380	ctg7180001899531_7766_SCT	36202512
25_3381	ctg7180001375123_307_SAC	36274327
25_3382	ctg7180001520488_372_SAG	36276763
25_3383	ctg7180001804695_3909_SCT	36278985
25_3384	ctg7180001923608_4682_SCT	36390386
25_3385	ctg7180001307068_395_SGT	36401273
25_3386	ctg7180001937291_552_SAG	36406913
25_3387	ctg7180001937290_956_SAG	36410741
25_3388	ctg7180001937290_760_SAC	36410937
25_3389	ctg7180001937289_5559_SCT	36420720
25_3390	ctg7180001937289_4979_SGT	36421300
25_3391	ctg7180001937289_3726_SAG	36422553

25_3392	ctg7180001937288_17583_SAC	36428073
25_3393	ctg7180001764527_596_SCT	36556781
25_3394	ctg7180001212467_1000_SCT	36558829
25_3395	ctg7180001212467_4008_SAC	36561837
25_3396	ctg7180001212467_6125_SAG	36563954
25_3397	ctg7180001806523_6657_SGT	36575172
25_3398	ctg7180001293729_272_SAG	36606011
25_3399	ctg7180001293729_3636_SAG	36609269
25_3400	ctg7180001838165_746_SCT	36624882
25_3401	ctg7180001838165_2267_SAC	36626450
25_3402	ctg7180001715466_1358_SAG	36633390
25_3403	ctg7180001573410_1656_SAC	36652276
25_3404	ctg7180001807835_8487_SAC	36656876
25_3405	ctg7180001599714_1090_SAG	36668474
25_3406	ctg7180001607206_128_SCT	36721777
25_3407	ctg7180001825719_397_SAG	36733478
25_3408	ctg7180001825719_3082_SCT	36736163
25_3409	ctg7180001930715_5561_SAG	36754629
25_3410	ctg7180001930715_4383_SAG	36755807
25_3411	ctg7180001930714_7521_SAG	36763488
25_3412	ctg7180001930714_2707_SGT	36768302
25_3413	ctg7180001721688_1665_SGT	36780885
25_3414	ctg7180001721688_2286_SCT	36781506
25_3415	ctg7180001721688_2552_SCT	36781772
25_3416	ctg7180001696623_295_SCT	36789875
25_3417	ctg7180001650838_980_SCT	36812581
25_3418	ctg7180001806111_71_SAG	36829405
25_3419	ctg7180001858753_3939_SGT	36904829
25_3420	ctg7180001320911_7072_SAC	36945600
25_3421	ctg7180001819681_1884_SGT	36962301
25_3422	ctg7180001819681_1896_SAG	36962313
25_3423	ctg7180001819681_2253_SAC	36962670
25_3424	ctg7180001525239_656_SCT	36979392
25_3425	ctg7180001388417_7472_SGT	36996346
25_3426	ctg7180001388417_129_SAG	37003689

25_3427	ctg7180001439357_1654_SGT	37139927
25_3428	ctg7180001934712_4017_SCT	37412613
25_3429	ctg7180001934712_4362_SCT	37412958
25_3430	ctg7180001706189_3532_SCT	37492465
25_3431	ctg7180001930498_2449_SCT	37512218
25_3432	ctg7180001909991_3884_SAG	37556315
25_3433	ctg7180001895983_2152_SAG	37629643
25_3434	ctg7180001932640_4780_SCT	37708543
25_3435	ctg7180001353466_43_SGT	37726448
25_3436	ctg7180001866202_6865_SCT	37830395
25_3437	ctg7180001866202_6903_SAG	37830433
25_3438	ctg7180001896928_1065_SCT	37861829
25_3439	ctg7180001896928_2562_SAG	37863326
25_3440	ctg7180001872472_5043_SAG	37877752
25_3441	ctg7180001925328_4264_SCT	37959549
25_3442	ctg7180001815765_5067_SCT	37969164
25_3443	ctg7180001611248_4628_SAC	37986766
25_3444	ctg7180001812093_5207_SAG	38009333
25_3445	ctg7180001807566_5772_SAG	38075175
25_3446	ctg7180001807566_1924_SGT	38079023
25_3447	ctg7180001663648_2145_SGT	38095088
25_3448	ctg7180001663648_1609_SCT	38095624
25_3449	ctg7180001906327_153_SAG	38112187
25_3450	ctg7180001906327_3334_SCT	38115368
25_3451	ctg7180001906327_3825_SCT	38115859
25_3452	ctg7180001906327_3861_SAC	38115895
25_3453	ctg7180001927394_688_SCT	38134248
25_3454	ctg7180001927394_537_SAG	38134399
25_3455	ctg7180001198593_563_SAG	38136620
25_3456	ctg7180001912953_3694_SAC	38162140
25_3457	ctg7180001912953_2045_SAC	38163789
25_3458	ctg7180001931306_1494_SAC	38168238
25_3459	ctg7180001931306_3187_SGT	38169931
25_3460	ctg7180001638564_2598_SAG	38263129
25_3461	ctg7180001638564_2335_SAG	38263392

25_3462	ctg7180001931306_7821_SAC	38324251
25_3463	ctg7180001797798_4356_SGT	38341974
25_3464	ctg7180001824716_1912_SAG	38351462
25_3465	ctg7180001893219_6092_SAG	38366648
25_3466	ctg7180001928137_1783_SGT	38388092
25_3467	ctg7180001919430_2034_SGT	38404366
25_3468	ctg7180001545874_1473_SCT	38428117
25_3469	ctg7180001545874_143_SCT	38429447
25_3470	ctg7180001477159_1384_SAC	38484454
25_3471	ctg7180001493382_283_SAC	38568680
25_3472	ctg7180001808239_338_SAC	38603108
25_3473	ctg7180001914491_3042_SAG	38630322
25_3474	ctg7180001786945_4526_SCT	38700671
25_3475	ctg7180001888094_7733_SGT	38702289
25_3476	ctg7180001888094_7481_SGT	38702540
25_3477	ctg7180001194303_2755_SAG	38724812
25_3478	ctg7180001799136_6079_SCT	38740569
25_3479	ctg7180001856618_14144_SGT	38757909
25_3480	ctg7180001811800_9159_SGT	38776331
25_3481	ctg7180001829102_1110_SAC	38790999
25_3482	ctg7180001594350_5475_SAG	38802784
25_3483	ctg7180001921959_4145_SAG	38826440
25_3484	ctg7180001489762_1111_SAG	38834091
25_3485	ctg7180001489762_2993_SAC	38835973
25_3486	ESTNV_35075_2399	38879342
25_3487	ESTNV_35075_1491	38881616
25_3488	ctg7180001430596_1883_SAG	38927996
25_3489	ctg7180001797847_2547_SAG	38942993
25_3490	ctg7180001317288_8994_SCT	38946947
25_3491	ctg7180001317288_8799_SAG	38947142
25_3492	ctg7180001317288_1869_SCT	38954072
25_3493	ctg7180001317288_464_SAC	38955477
25_3494	ctg7180001797942_2235_SCT	38964253
25_3495	ctg7180001790982_2267_SAC	38983026
25_3496	ctg7180001929120_2599_SAG	38992903

25_3497	ctg7180001929120_2683_SGT	38992987
25_3498	ctg7180001929120_3152_SAG	38993456
25_3499	ctg7180001929120_3342_SCT	38993646
25_3500	ctg7180001929120_3984_SAG	38994288
25_3501	ctg7180001929120_7422_SAC	38997726
25_3502	ctg7180001929120_8591_SAC	38998895
25_3503	ctg7180001373157_928_SAC	39014684
25_3504	ctg7180001373157_1473_SGT	39015229
25_3505	ctg7180001909236_8465_SAC	39063133
25_3506	ctg7180001691013_2136_SCT	39097601
25_3507	ctg7180001691013_4173_SAC	39099850
25_3508	ctg7180001877675_7697_SAG	39107761
25_3509	ctg7180001866903_6237_SAC	39155393
25_3510	ctg7180001278478_108_SAG	39185014
25_3511	ctg7180001802356_8460_SAG	39193642
25_3512	ctg7180001802356_6277_SAC	39195825
25_3513	ctg7180001806522_179_SCT	39216005
25_3514	ctg7180001825157_1489_SGT	39265012
25_3515	ctg7180001871759_1824_SGT	39286386
25_3516	ctg7180001871759_1370_SCT	39286840
25_3517	ctg7180001871759_1337_SAC	39286873
25_3518	ctg7180001836980_9709_SCT	39294302
25_3519	ctg7180001836980_5122_SAG	39298889
25_3520	ctg7180001821878_4759_SGT	39311560
25_3521	ctg7180001850082_922_SCT	39347032
25_3522	ctg7180001850082_14187_SGT	39360298
25_3523	ctg7180001850082_15838_SGT	39361949
25_3524	ctg7180001850082_15889_SAG	39362000
25_3525	ctg7180001850082_20542_SGT	39366592
25_3526	ctg7180001880876_5821_SAC	39384181
25_3527	ctg7180001435353_1769_SAG	39394104
25_3528	ctg7180001251528_5445_SCT	39397454
25_3529	ctg7180001251528_3866_SAG	39399033
25_3530	ctg7180001251528_1713_SGT	39401186
25_3531	ctg7180001251528_1706_SGT	39401193

25_3532	ctg7180001251528_1444_SAC	39401455
25_3533	ctg7180001880875_3959_SAC	39428074
25_3534	ctg7180001904006_8303_SAG	39444592
25_3535	ctg7180001816619_3540_SCT	39502200
25_3536	ctg7180001914412_7672_SGT	39524061
25_3537	ctg7180001914412_7846_SAG	39524235
25_3538	ctg7180001914412_7895_SCT	39524284
25_3539	ctg7180001914413_2276_SAG	39529445
25_3540	ctg7180001757141_1558_SCT	39577192
25_3541	ctg7180001733228_2112_SCT	39592553
25_3542	ctg7180001733228_1947_SCT	39592718
25_3543	ctg7180001930525_7053_SAC	39601915
25_3544	ctg7180001930525_7108_SAC	39601970
25_3545	ctg7180001930525_16587_SAG	39611449
25_3546	ctg7180001930525_20305_SCT	39615167
25_3547	ctg7180001320700_628_SCT	39632090
25_3548	ctg7180001320700_69_SCT	39632649
25_3549	ctg7180001923565_9056_SCT	39648501
25_3550	ctg7180001923565_16116_SAG	39655561
25_3551	ctg7180001923565_19494_SCT	39658939
25_3552	ctg7180001923565_19814_SGT	39659259
25_3553	ctg7180001923565_24993_SGT	39664438
25_3554	ctg7180001814067_3381_SGT	39669797
25_3555	ctg7180001814067_4352_SCT	39670768
25_3556	ctg7180001814067_7250_SAG	39673666
25_3557	ctg7180001814067_7283_SAG	39673699
25_3558	ctg7180001814067_7349_SCT	39673765
25_3559	ctg7180001897293_5388_SCT	39692417
25_3560	ctg7180001897293_4564_SAC	39693241
25_3561	ctg7180001795864_5213_SCT	39712289
25_3562	ctg7180001795864_5736_SAG	39712812
25_3563	ctg7180001515826_325_SCT	39723723
25_3564	ctg7180001515826_146_SAG	39723902
25_3565	ctg7180001489176_1740_SAC	39725453
25_3566	ctg7180001881277_2935_SAG	39729496

25_3567	ctg7180001822899_984_SAC	39741105
25_3568	ctg7180001822899_4359_SAG	39744443
25_3569	ctg7180001828845_119_SCT	39763529
25_3570	ctg7180001868174_13715_SAG	39809439
25_3571	ctg7180001868174_14185_SAC	39809909
25_3572	ctg7180001296062_1258_SGT	39825721
25_3573	ctg7180001296062_894_SAC	39826085
25_3574	ctg7180001896259_3597_SCT	39834193
25_3575	ctg7180001801880_1171_SGT	39852264
25_3576	ctg7180001801880_5064_SAG	39856158
25_3577	ctg7180001488078_1966_SGT	39866600
25_3578	ctg7180001488078_3003_SAG	39867637
25_3579	ctg7180001794010_7928_SCT	39897822
25_3580	ctg7180001426009_4985_SAC	39925406
25_3581	ctg7180001426009_5781_SGT	39926202
25_3582	ctg7180001871447_11348_SAC	39943809
25_3583	ctg7180001871447_11924_SCT	39944385
25_3584	ctg7180001594155_1032_SCT	39957821
25_3585	ctg7180001208282_6016_SAC	40010722
25_3586	ctg7180001403191_9144_SAG	40184710
25_3587	ctg7180001682434_3439_SCT	40190533
25_3588	ctg7180001925312_10654_SCT	40223821
25_3589	ctg7180001925312_10641_SCT	40223834
25_3590	ctg7180001925312_10450_SAC	40224025
25_3591	ctg7180001925312_10325_SAC	40224150
25_3592	ctg7180001925312_6732_SAC	40227743
25_3593	ctg7180001841276_1951_SGT	40241764
25_3594	ctg7180001841276_1944_SAG	40241771
25_3595	ctg7180001920350_7954_SAG	40322051
25_3596	ctg7180001638286_1162_SCT	40361457
25_3597	ctg7180001813248_4179_SCT	40458836
25_3598	ctg7180001813248_3700_SCT	40459315
25_3599	ctg7180001902705_5233_SAG	40483067
25_3600	ctg7180001902705_6184_SAG	40484018
25_3601	ctg7180001853604_245_SAG	40507254

25_3602	ctg7180001853604_1349_SAG	40508358
25_3603	ctg7180001891636_1837_SCT	40528576
25_3604	ctg7180001781568_2070_SCT	40541505
25_3605	ctg7180001799429_2412_SAG	40563190
25_3606	ctg7180001897418_1684_SCT	40607388
25_3607	ctg7180001897418_2415_SAG	40608119
25_3608	ctg7180001897419_2421_SGT	40620697
25_3609	ctg7180001858930_812_SAG	40685711
25_3610	ctg7180001919270_10331_SCT	40720733
25_3611	ctg7180001804778_4760_SAC	40742852
25_3612	ctg7180001905481_20848_SCT	40780064
25_3613	ctg7180001905481_12249_SAG	40788663
25_3614	ctg7180001905481_8936_SAG	40791976
25_3615	ctg7180001911152_7255_SAG	40822472
25_3616	ctg7180001651398_4884_SGT	40853880
25_3617	ctg7180001932749_8336_SAG	40879110
25_3618	ctg7180001872830_5110_SCT	40894071
25_3619	ctg7180001880624_4924_SCT	40925196
25_3620	ctg7180001833023_2115_SCT	40985092
25_3621	ctg7180001923559_9689_SGT	41001300
25_3622	ctg7180001275466_1089_SAC	41038984
25_3623	ctg7180001821705_5967_SAG	41053276
25_3624	ctg7180001821705_892_SAC	41058351
25_3625	ctg7180001247253_959_SGT	41087126
25_3626	ctg7180001240845_4025_SCT	41106474
25_3627	ctg7180001240845_4238_SGT	41106687
25_3628	ctg7180001545165_791_SAC	41151131
25_3629	ctg7180001318321_6644_SCT	41171273
25_3630	ctg7180001589310_976_SGT	41206293
25_3631	ctg7180001870874_1322_SGT	41286409
25_3632	ctg7180001873646_2227_SGT	41319145
25_3633	ctg7180001850299_7142_SCT	41325051
25_3634	ctg7180001850299_7079_SAG	41325114
25_3635	ctg7180001850299_5120_SCT	41327073
25_3636	ctg7180001850299_5076_SCT	41327117

25_3637	ctg7180001850299_4566_SGT	41327627
25_3638	ctg7180001872178_2728_SGT	41415375
25_3639	ctg7180001872178_1562_SCT	41416541
25_3640	ctg7180001884283_4242_SGT	41424187
25_3641	ctg7180001907756_1586_SAG	41440003
25_3642	ctg7180001835189_2287_SAC	41465129
25_3643	ctg7180001254234_1712_SAG	41485603
25_3644	ctg7180001875289_5437_SCT	41493804
25_3645	ctg7180001875290_172_SAC	41497739
25_3646	ctg7180001306227_153_SAC	41543893
25_3647	ctg7180001642110_2474_SCT	41573368
25_3648	ctg7180001327405_1208_SCT	41581174
25_3649	ctg7180001696701_890_SGT	41657095
25_3650	ctg7180001568745_57_SAG	41665699
25_3651	ctg7180001596671_1701_SCT	41692328
25_3652	ctg7180001352449_592_SAC	41712442
25_3653	ctg7180001471130_6094_SGT	41722421
25_3654	ctg7180001342354_1779_SCT	41735509
25_3655	ctg7180001826029_2717_SAG	41740687
25_3656	ctg7180001826029_3256_SCT	41741226
25_3657	ctg7180001826029_4235_SAG	41742205
25_3658	ctg7180001826029_5000_SCT	41742970
25_3659	ctg7180001826029_10984_SGT	41748954
25_3660	ctg7180001656076_6249_SGT	41775109
25_3661	ctg7180001876905_16212_SAG	41820629
25_3662	ctg7180001935544_15997_SCT	41852437
25_3663	ctg7180001935544_11892_SAC	41856542
25_3664	ctg7180001620626_8398_SAC	41895194
25_3665	ctg7180001812941_2831_SGT	41937602
25_3666	ctg7180001867454_11336_SAC	41950208
25_3667	ctg7180001856031_1360_SAG	41970334
25_3668	ctg7180001856031_2233_SCT	41971171
25_3669	ctg7180001902401_1458_SCT	41976666
25_3670	ctg7180001841217_6231_SCT	41996702
25_3671	ctg7180001799947_7339_SAG	42209217

25_3672	ctg7180001500755_561_SAC	42217259
25_3673	ctg7180001739963_199_SGT	42220494
25_3674	ctg7180001897158_2075_SAG	42237600
25_3675	ctg7180001609406_4203_SCT	42277707
25_3676	ctg7180001609406_3965_SAC	42277945
25_3677	ctg7180001609406_3244_SAG	42278666
25_3678	ctg7180001929658_8998_SGT	42301362
25_3679	ctg7180001929658_10448_SAG	42302812
25_3680	ctg7180001929658_12842_SAG	42305206
25_3681	ctg7180001819946_4375_SCT	42312385
25_3682	ctg7180001286348_1764_SCT	42361504
25_3683	ctg7180001240142_1942_SAG	42380131
25_3684	ctg7180001240142_2450_SGT	42380604
25_3685	ctg7180001931392_1596_SGT	42387697
25_3686	ctg7180001805727_7788_SAC	42443870
25_3687	ctg7180001696277_10111_SCT	42482405
25_3688	ctg7180001696277_10303_SAG	42482597
25_3689	ctg7180001871904_8023_SAG	42525971
25_3690	ctg7180001726564_4001_SAG	42547888
25_3691	ctg7180001654764_1375_SGT	42556175
25_3692	ctg7180001654764_981_SCT	42556569
25_3693	ctg7180001850459_3700_SCT	42565854
25_3694	ctg7180001850459_5900_SAG	42568053
25_3695	ctg7180001773466_993_SAG	42598278
25_3696	ctg7180001773466_1135_SAG	42598420
25_3697	ctg7180001912641_218_SAG	42601820
25_3698	ctg7180001912641_2690_SCT	42604292
25_3699	ctg7180001912641_2881_SGT	42604483
25_3700	ctg7180001912641_2976_SAC	42604578
25_3701	ctg7180001879326_9740_SGT	42625163
25_3702	ctg7180001879326_9498_SGT	42625405
25_3703	ctg7180001515882_5961_SAG	42647526
25_3704	ctg7180001797588_1455_SAC	42673865
25_3705	ctg7180001797588_712_SAC	42674608
25_3706	ctg7180001797588_49_SGT	42675271

25_3707	ctg7180001578987_1479_SAG	42683877
25_3708	ctg7180001836276_5084_SAG	42693544
25_3709	ctg7180001836276_6423_SCT	42694883
25_3710	ctg7180001836276_6536_SAG	42694996
25_3711	ctg7180001853248_1848_SAG	42706392
25_3712	ctg7180001868826_1179_SAC	42719115
25_3713	ctg7180001868826_1254_SAC	42720608
25_3714	ctg7180001401372_970_SAG	42723688
25_3715	ctg7180001401372_1132_SCT	42723850
25_3716	ctg7180001788005_4822_SAC	42815340
25_3717	ctg7180001450836_1070_SGT	42892406
25_3718	ctg7180001784652_3824_SAC	42921201
25_3719	ctg7180001740045_1424_SCT	42945895
25_3720	ctg7180001233515_8417_SCT	42952436
25_3721	ctg7180001233515_7328_SAG	42953525
25_3722	ctg7180001233515_7168_SCT	42953685
25_3723	ctg7180001233515_4431_SAG	42956422
25_3724	ctg7180001538255_551_SAC	43047414
25_3725	ctg7180001824035_6369_SCT	43050074
25_3726	ctg7180001336314_1109_SAG	43072907
25_3727	ctg7180001553506_246_SAG	43079895
25_3728	ctg7180001796379_8739_SGT	43124980
25_3729	ctg7180001808073_3948_SAC	43158227
25_3730	ctg7180001336310_2006_SCT	43167371
25_3731	ctg7180001336310_2384_SGT	43167784
25_3732	ctg7180001491895_1989_SCT	43192936
25_3733	ctg7180001608778_1966_SAC	43195490
25_3734	ctg7180001351933_1569_SAC	43204623
25_3735	ctg7180001351933_1648_SAG	43204702
25_3736	ctg7180001351933_2444_SAC	43205498
25_3737	ctg7180001351933_2717_SAG	43205771
25_3738	ctg7180001908277_8211_SCT	43221605
25_3739	ctg7180001908277_12454_SGT	43225848
25_3740	ctg7180001908277_12682_SAC	43226076
25_3741	ctg7180001908277_16874_SAG	43230268

25_3742	ctg7180001908277_18503_SCT	43231888
25_3743	ctg7180001908277_18805_SGT	43232190
25_3744	ctg7180001908277_18964_SGT	43232349
25_3745	ctg7180001908277_20222_SCT	43233607
25_3746	ctg7180001843602_1101_SCT	43260396
25_3747	ctg7180001883143_3121_SAG	43276534
25_3748	ctg7180001693530_1923_SCT	43278615
25_3749	ctg7180001693530_2807_SGT	43279499
25_3750	ctg7180001425622_278_SGT	43286432
25_3751	ctg7180001432015_488_SAG	43289649
25_3752	ctg7180001692307_5823_SAC	43341709
25_3753	ctg7180001427839_1398_SAG	43373203
25_3754	ctg7180001427839_1616_SGT	43373421
25_3755	ctg7180001889616_5095_SAG	43413016
25_3756	ctg7180001860070_2960_SAC	43425570
25_3757	ctg7180001860070_2330_SAC	43426198
25_3758	ctg7180001930377_6537_SCT	43436889
25_3759	ctg7180001930377_13521_SAG	43443873
25_3760	ctg7180001930377_14766_SAG	43445118
25_3761	ctg7180001852725_3123_SAC	43456940
25_3762	ctg7180001813999_3647_SAG	43472299
25_3763	ctg7180001520493_200	43474015
25_3764	ctg7180001520493_1122_SCT	43479465
25_3765	ctg7180001520493_924_SCT	43479663
25_3766	ctg7180001231556_1466_SAG	43483729
25_3767	ctg7180001894549_948_SAG	43500819
25_3768	ctg7180001894549_3007_SAG	43502913
25_3769	ctg7180001894549_3124_SGT	43502995
25_3770	ctg7180001888237_1542_SAG	43506033
25_3771	ctg7180001707543_7969_SGT	43518218
25_3772	ctg7180001463430_1296_SAC	43526533
25_3773	ctg7180001696778_2762_SCT	43529917
25_3774	ctg7180001696778_2752_SCT	43529927
25_3775	ctg7180001696778_1385_SGT	43531294
25_3776	ctg7180001804953_6991_SAC	43536974

25_3777	ctg7180001804953_6700_SAC	43537265
25_3778	ctg7180001804953_740_SAG	43543171
25_3779	ctg7180001919151_4221_SGT	43560058
25_3780	ctg7180001919151_1820_SAC	43562459
25_3781	ctg7180001919151_711_SCT	43563568
25_3782	ctg7180001358007_2364_SAG	43574154
25_3783	ctg7180001358007_679_SCT	43575839
25_3784	ctg7180001593404_1147_SCT	43594225
25_3785	ctg7180001863775_5130_SAG	43600726
25_3786	ctg7180001863775_7925_SCT	43603522
25_3787	ctg7180001478419_693_SAC	43608320
25_3788	ctg7180001855273_7371_SAG	43701502
25_3789	ctg7180001855273_5156_SAG	43704627
25_3790	ctg7180001850582_4700_SCT	43737890
25_3791	ctg7180001850582_4762_SAC	43737952
25_3792	ctg7180001915846_5740_SGT	43755945
25_3793	ctg7180001783416_1513_SAG	43762302
25_3794	ctg7180001783416_1312_SCT	43762503
25_3795	ctg7180001783416_211_SAG	43763604
25_3796	ctg7180001810851_4190_SCT	43795007
25_3797	ctg7180001810851_4664_SCT	43795481
25_3798	ctg7180001810851_4812_SAC	43795629
25_3799	ctg7180001810851_5321_SGT	43796138
25_3800	ctg7180001810851_5465_SAG	43796282
25_3801	ctg7180001810851_5600_SGT	43796417
25_3802	ctg7180001835087_15459_SAG	43820803
25_3803	ctg7180001835087_16838_SCT	43822182
25_3804	ctg7180001835087_20319_SGT	43825663
25_3805	ctg7180001352971_3941_SAG	43842782
25_3806	ctg7180001352971_1862_SAG	43844861
25_3807	ctg7180001794549_1606_SGT	43857493
25_3808	ctg7180001915705_8467_SGT	43931416
25_3809	ctg7180001557868_115_SAG	43940856
25_3810	ctg7180001295015_2729_SCT	43985496
25_3811	ctg7180001295015_2180_SCT	43986045

25_3812	ctg7180001439131_1750_SCT	44012509
25_3813	ctg7180001439131_2077_SGT	44012836
25_3814	ctg7180001826020_3843_SCT	44054975
25_3815	ctg7180001826020_13310_SCT	44064442
25_3816	ctg7180001569088_942_SAC	44099574
25_3817	ctg7180001691820_3284_SAG	44228853
25_3818	ctg7180001458584_1304_SCT	44234375
25_3819	ctg7180001499576_1224_SGT	44271568
25_3820	ctg7180001626292_1520_SAG	44278108
25_3821	ctg7180001605858_1387_SAG	44296472
25_3822	ctg7180001271079_4684_SAG	44309552
25_3823	ctg7180001797990_4902_SAG	44426070
25_3824	ctg7180001748718_1051_SGT	44427192
25_3825	ctg7180001818321_3341_SAG	44435750
25_3826	ctg7180001266183_1852_SGT	44450943
25_3827	ctg7180001491589_2156_SAC	44476474
25_3828	ctg7180001703092_946_SAG	44494153
25_3829	ctg7180001703092_910_SCT	44494189
25_3830	ctg7180001794026_2249_SAG	44512073
25_3831	ctg7180001795145_5835_SCT	44544881
25_3832	ctg7180001930194_10805_SGT	44580831
25_3833	ctg7180001593900_1570_SCT	44595220
25_3834	ctg7180001222093_767_SCT	44617688
25_3835	ctg7180001932490_1923_SAG	44748906
25_3836	ctg7180001823664_4028_SAC	44759420
25_3837	ctg7180001872208_6542_SCT	44797952
25_3838	ctg7180001594003_97_SAG	44803594
25_3839	ctg7180001803278_514_SCT	44870888
25_3840	ctg7180001807475_2946_SAC	44918052
25_3841	ctg7180001807475_3507_SCT	44918613
25_3842	ctg7180001217047_123_SCT	44937905
25_3843	ctg7180001863577_454_SAG	44953455
25_3844	ctg7180001863577_361_SAG	44953548
25_3845	ctg7180001791271_5157_SCT	44960029
25_3846	ctg7180001902060_7697_SAC	45018529

25_3847	ctg7180001653430_999_SAG	45024575
25_3848	ctg7180001800907_81_SGT	45084534
25_3849	ctg7180001910701_7414_SAG	45092422
25_3850	ctg7180001920546_4771_SCT	45108215
25_3851	ctg7180001920546_6024_SCT	45109468
25_3852	ctg7180001920546_6758_SAG	45110202
25_3853	ctg7180001710343_214_SAG	45130885
25_3854	ctg7180001226839_1082_SAG	45138380
25_3855	ctg7180001866719_1068_SAG	45158912
25_3856	ctg7180001566902_345_SAG	45160744
25_3857	ctg7180001383469_1162_SAG	45162887
25_3858	ctg7180001932718_5944_SGT	45172092
25_3859	ctg7180001932718_5266_SGT	45172404
25_3860	ctg7180001932718_5100_SAG	45172570
25_3861	ctg7180001932718_4995_SCT	45172675
25_3862	ctg7180001932718_4971_SAC	45172699
25_3863	ctg7180001932718_2925_SAG	45174745
25_3864	ctg7180001932718_1866_SAG	45175705
25_3865	ctg7180001557995_284_SGT	45177835
25_3866	ctg7180001557995_349_SAG	45177900
25_3867	ctg7180001786677_14248_SCT	45205593
25_3868	ctg7180001786677_10893_SCT	45208948
25_3869	ctg7180001786677_7794_SAG	45212047
25_3870	ctg7180001786676_1876_SGT	45220835
25_3871	ctg7180001786676_532_SAC	45222179
25_3872	ctg7180001795427_569_SAG	45227148
25_3873	ctg7180001273465_191_SAG	45231673
25_3874	ctg7180001273465_94_SAG	45231770
25_3875	ctg7180001835519_282_SAG	45238291
25_3876	ctg7180001835519_4567_SGT	45242576
25_3877	ctg7180001469733_2114_SAG	45251429
25_3878	ctg7180001830665_1953_SCT	45289134
25_3879	ctg7180001830665_1840_SAC	45289247
25_3880	ctg7180001242649_849_SCT	45299869
25_3881	ctg7180001931998_8169_SAG	45303455

25_3882	ctg7180001931998_7885_SAC	45303739
25_3883	ctg7180001828728_4787_SAC	45317626
25_3884	ctg7180001833618_3495_SGT	45326608
25_3885	ctg7180001807318_4273_SAG	45347549
25_3886	ctg7180001807318_2793_SAC	45349029
25_3887	ctg7180001864616_1043_SCT	45383917
25_3888	ctg7180001881262_3020_SCT	45403136
25_3889	ctg7180001804882_3646_SAC	45415923
25_3890	ctg7180001804882_2181_SAG	45417528
25_3891	ctg7180001804882_140_SAG	45419569
25_3892	ctg7180001906402_9230_SGT	45457058
25_3893	ctg7180001906402_12896_SCT	45460724
25_3894	ctg7180001636845_879_SCT	45546790
25_3895	ctg7180001531947_2204_SAG	45551455
25_3896	ctg7180001531947_1738_SGT	45551921
25_3897	ctg7180001210639_1152_SAG	45590974
25_3898	ctg7180001511071_536_SAG	45653797
25_3899	ctg7180001511071_528_SCT	45653805
25_3900	ctg7180001832026_190_SCT	45654557
25_3901	GCR_cBin33290_Ctg1_231	45658966
25_3902	ctg7180001864573_1144_SAC	45672582
25_3903	ctg7180001507154_567_SGT	45703183
25_3904	ctg7180001625587_1560_SAC	45711857
25_3905	ctg7180001625587_2240_SGT	45712537
25_3906	ctg7180001625587_2310_SAG	45712607
25_3907	ctg7180001625587_2562_SAG	45712859
25_3908	ctg7180001820378_7615_SGT	45717943
25_3909	ctg7180001820378_5715_SCT	45720206
25_3910	ctg7180001820378_3890_SAG	45722031
25_3911	ctg7180001820378_3669_SAG	45722252
25_3912	ctg7180001820378_3316_SAG	45722605
25_3913	ctg7180001820378_2496_SGT	45723425
25_3914	ctg7180001817424_2064_SCT	45726582
25_3915	ctg7180001917641_1376_SAG	45735491
25_3916	ctg7180001874082_5542_SCT	45762224

25_3917	ctg7180001874082_5064_SAG	45762702
25_3918	ctg7180001874082_1241_SAC	45766525
25_3919	ctg7180001338679_690_SGT	45803731
25_3920	ctg7180001338679_604_SCT	45803817
25_3921	ctg7180001290344_743_SCT	45805659
25_3922	ctg7180001290344_819_SGT	45805735
25_3923	ctg7180001290344_1137_SGT	45806053
25_3924	ctg7180001912023_6766_SCT	45814819
25_3925	ctg7180001605481_1437_SGT	45848581
25_3926	ctg7180001859373_1448_SGT	45905427
25_3927	ctg7180001859373_1378_SAC	45905497
25_3928	ctg7180001344465_1116_SAC	45908988
25_3929	ctg7180001885099_11372_SAG	45914897
25_3930	ctg7180001885099_10950_SGT	45915319
25_3931	ctg7180001885099_10656_SAG	45915613
25_3932	ctg7180001885099_5645_SGT	45920624
25_3933	ctg7180001373892_523_SAG	45946280
25_3934	ctg7180001800385_2330_SAC	45953124
25_3935	ctg7180001859290_5392_SCT	45963226
25_3936	ctg7180001873919_15292_SCT	45984811
25_3937	ctg7180001873919_16710_SAG	45986229
25_3938	ctg7180001873919_19426_SAC	45988584
25_3939	ctg7180001873919_19853_SCT	45989011
25_3940	ctg7180001677004_346_SCT	46001007
25_3941	ctg7180001921387_13849_SAC	46015011
25_3942	ctg7180001921387_19184_SCT	46023801
25_3943	ctg7180001921387_20257_SGT	46024874
25_3944	ctg7180001921387_25833_SAC	46030450
25_3945	ctg7180001921387_26261_SCT	46031087
25_3946	ctg7180001921387_27296_SAG	46032122
25_3947	ctg7180001611913_279_SCT	46059824
25_3948	ctg7180001815098_1511_SAC	46068509
25_3949	ctg7180001815098_15482_SAC	46082480
25_3950	ctg7180001815098_15692_SCT	46082690
25_3951	ctg7180001815098_16862_SAG	46083860

25_3952	ctg7180001816567_606_SAG	46089714
25_3953	ctg7180001659051_1259_SAG	46106875
25_3954	ctg7180001659082_51_SAG	46109267
25_3955	ctg7180001377232_1396_SCT	46127467
25_3956	ctg7180001476285_5756_SAG	46141238
25_3957	ctg7180001837002_8727_SCT	46162754
25_3958	ctg7180001837002_6631_SAC	46164849
25_3959	ctg7180001837002_6010_SAC	46165470
25_3960	ctg7180001838839_1722_SAC	46278710
25_3961	ctg7180001838839_1858_SAG	46278846
25_3962	ctg7180001838839_1995_SCT	46278983
25_3963	ctg7180001905612_2912_SAC	46316295
25_3964	ctg7180001804873_3040_SAG	46363906
25_3965	ctg7180001856452_8840_SAC	46427195
25_3966	ctg7180001868898_5995_SAC	46466534
25_3967	ctg7180001902652_12606_SAC	46493997
25_3968	ctg7180001896899_4452_SCT	46633989
25_3969	ctg7180001695843_2129_SAG	46641526
25_3970	ctg7180001872779_10369_SGT	46667468
25_3971	ctg7180001872779_8765_SAG	46669072
25_3972	ctg7180001857011_10580_SGT	46683375
25_3973	ctg7180001522043_1607_SGT	46709044
25_3974	ctg7180001903013_195_SAG	46788218
25_3975	ctg7180001555365_700_SAG	46899326
25_3976	ctg7180001862096_6664_SAC	46938944
25_3977	ctg7180001884823_1256_SAC	46945117
25_3978	ctg7180001289516_1491_SCT	46960320
25_3979	ctg7180001831177_10555_SAG	46993832
25_3980	ctg7180001820515_6114_SCT	47015810
25_3981	ctg7180001820515_781_SCT	47021143
25_3982	ctg7180001800894_11262_SCT	47057550
25_3983	ctg7180001827948_2173_SAG	47106498
25_3984	ctg7180001803475_718_SCT	47131498
25_3985	ctg7180001803476_527_SGT	47140901
25_3986	ctg7180001927070_3681_SAG	47219643

25_3987	ctg7180001927071_15737_SCT	47239847
25_3988	ctg7180001900949_5066_SAG	47316990
25_3989	ctg7180001900951_1499_SCT	47344628
25_3990	ctg7180001900951_4417_SCT	47347267
25_3991	ctg7180001804843_357_SAC	47366300
25_3992	ctg7180001548284_512_SAC	47388827
25_3993	ctg7180001876994_10088_SAG	47408456
25_3994	ctg7180001876994_8266_SAG	47410278
25_3995	ctg7180001798113_1637_SAG	47449414
25_3996	ctg7180001798113_7942_SGT	47455716
25_3997	ctg7180001847992_5063_SAG	47479990
25_3998	ctg7180001542577_921_SAG	47514079
25_3999	ctg7180001896686_3281_SAG	47565013
25_4000	ctg7180001929944_9248_SGT	47571595
25_4001	ctg7180001879178_3430_SAG	47589667
25_4002	ctg7180001879178_3466_SAG	47589703
25_4003	ctg7180001879178_5555_SCT	47591792
25_4004	ctg7180001879178_6702_SGT	47592939
25_4005	ctg7180001796110_2919_SGT	47620553
25_4006	ctg7180001814628_1587_SAG	47687058
25_4007	ctg7180001814628_1040_SCT	47687605
25_4008	ctg7180001814628_879_SAG	47687766
25_4009	ctg7180001274958_340_SAG	47742491
25_4010	ctg7180001683243_879_SCT	47753438
25_4011	ctg7180001683243_1130_SAG	47753689
25_4012	ctg7180001683243_1274_SCT	47753833
25_4013	ctg7180001898454_8737_SAG	47783212
25_4014	ctg7180001926259_8222_SAG	47796213
25_4015	ctg7180001834024_2117_SAG	47819167
25_4016	ctg7180001834024_3005_SCT	47820294
25_4017	ctg7180001837343_2117_SCT	47847958
25_4018	ctg7180001837343_1138_SGT	47848937
25_4019	ctg7180001837343_1005_SCT	47849070
25_4020	ctg7180001906082_3253_SAG	47863722
25_4021	ctg7180001934857_1497_SAC	47898653

25_4022	ctg7180001281887_344_SGT	47927180
25_4023	ctg7180001281887_635_SGT	47927471
25_4024	ctg7180001557269_358_SAC	47938952
25_4025	ctg7180001557269_636_SCT	47939230
25_4026	ctg7180001557269_639_SAG	47939233
25_4027	ctg7180001218407_2858_SAG	47951465
25_4028	ctg7180001218407_3133_SCT	47951740
25_4029	ctg7180001936474_3894_SGT	47955856
25_4030	ctg7180001428309_626_SAC	47971904
25_4031	ctg7180001821429_1432_SCT	47978691
25_4032	ctg7180001821429_1594_SGT	47978853
25_4033	ctg7180001821429_4467_SAG	47981726
25_4034	ctg7180001880109_855_SAG	47992790
25_4035	ctg7180001880109_1129_SCT	47993064
25_4036	ctg7180001889357_1493_SAG	48011162
25_4037	ctg7180001889357_1782_SAG	48011451
25_4038	ctg7180001889357_2309_SGT	48011978
25_4039	ctg7180001884800_3113_SAC	48055539
25_4040	ctg7180001933090_7619_SAG	48070507
25_4041	ctg7180001911153_6813_SAG	48109987
25_4042	ctg7180001929107_3654_SAG	48119288
25_4043	ctg7180001929107_3823_SCT	48119457
25_4044	ctg7180001929107_5129_SCT	48120763
25_4045	ctg7180001789397_2856_SCT	48158993
25_4046	ctg7180001789397_739_SAC	48161367
25_4047	ctg7180001926452_9216_SAG	48165821
25_4048	ctg7180001848176_10854_SAG	48207997
25_4049	ctg7180001848176_11355_SAC	48208498
25_4050	ctg7180001848176_14848_SAG	48211991
25_4051	ctg7180001848176_15474_SCT	48212617
25_4052	ctg7180001821557_3212_SCT	48216525
25_4053	ctg7180001828941_531_SGT	48218754
25_4054	ctg7180001828941_2486_SAC	48220709
25_4055	ctg7180001911153_8859_SAC	48239651
25_4056	ctg7180001935672_99_SCT	48260597

25_4057	ctg7180001935672_2364_SCT	48262862
25_4058	ctg7180001935672_6901_SAC	48267433
25_4059	ctg7180001935672_7761_SAG	48268109
25_4060	ctg7180001935672_8590_SAC	48268938
25_4061	ctg7180001935672_9507_SAG	48269856
25_4062	ctg7180001925244_3881_SCT	48329876
25_4063	ctg7180001903278_7952_SAC	48370076
25_4064	ctg7180001903278_8656_SAG	48370780
25_4065	ctg7180001571150_1260_SAC	48390617
25_4066	ctg7180001897585_12510_SGT	48391932
25_4067	ctg7180001897585_6036_SAC	48398406
25_4068	ctg7180001897585_5632_SAG	48398810
25_4069	ctg7180001897585_5558_SAG	48398884
25_4070	ctg7180001805087_10243_SGT	48423056
25_4071	ctg7180001805087_10152_SGT	48423147
25_4072	ctg7180001805087_10098_SAC	48423201
25_4073	ctg7180001805087_5731_SCT	48427568
25_4074	ctg7180001866595_4403_SCT	48470837
25_4075	ctg7180001866595_1774_SAG	48473466
25_4076	ctg7180001866595_1610_SCT	48473630
25_4077	ctg7180001831774_8277_SGT	48494305
25_4078	ctg7180001920138_1781_SGT	48508296
25_4079	ctg7180001932904_246_SCT	48520114
25_4080	ctg7180001826421_6469_SAG	48531819
25_4081	ctg7180001831776_2166_SAG	48541196
25_4082	ctg7180001250564_447_SCT	48576350
25_4083	ctg7180001190430_644_SAG	48577411
25_4084	ctg7180001190430_1597_SCT	48578364
25_4085	ctg7180001190430_2109_SAC	48578876
25_4086	ctg7180001724564_1287_SAC	48583305
25_4087	ctg7180001724564_999_SCT	48583593
25_4088	ctg7180001724564_913_SAG	48583679
25_4089	ctg7180001808294_60_SGT	48611937
25_4090	ctg7180001669420_2227_SAC	48701892
25_4091	ctg7180001858702_4466_SAG	48756341

25_4092	ctg7180001934498_6284_SGT	48767123
25_4093	ctg7180001503621_1427_SAG	48799110
25_4094	ctg7180001824958_164_SCT	48811321
25_4095	ctg7180001824958_211_SCT	48811368
25_4096	ctg7180001931435_11163_SAG	48834017
25_4097	ctg7180001822193_4306_SGT	48871255
25_4098	ctg7180001828332_1568_SAC	48901613
25_4099	ctg7180001929991_4587_SGT	48915286
25_4100	ctg7180001929991_2627_SAG	48917246
25_4101	ctg7180001934616_1269_SCT	48959285
25_4102	ctg7180001823536_3031_SAC	49007619
25_4103	ctg7180001823536_2888_SAC	49007762
25_4104	ctg7180001485330_1017_SCT	49013919
25_4105	ctg7180001685596_1323_SAG	49022393
25_4106	ctg7180001911228_5661_SAG	49084843
25_4107	ctg7180001250508_1011_SAC	49099068
25_4108	ctg7180001296149_1291_SGT	49188267
25_4109	ctg7180001368370_1892_SAC	49195880
25_4110	ctg7180001716897_943_SAG	49215148
25_4111	ctg7180001811615_5463_SAG	49226655
25_4112	ctg7180001811615_3300_SAG	49228818
25_4113	ctg7180001214739_215_SAG	49285585
25_4114	ctg7180001623302_1695_SAC	49293072
25_4115	ctg7180001623302_1291_SCT	49293476
25_4116	ctg7180001623302_78_SCT	49294689
25_4117	ctg7180001525824_1233_SAG	49310856
25_4118	ctg7180001827443_962_SCT	49332481
25_4119	ctg7180001341866_170_SAC	49355879
25_4120	ctg7180001843336_6324_SCT	49363904
25_4121	ctg7180001843336_6051_SAC	49364177
25_4122	ctg7180001819588_4042_SCT	49394933
25_4123	ctg7180001884889_1043_SAG	49423815
25_4124	ctg7180001712405_1795_SCT	49449260
25_4125	ctg7180001841245_3610_SAC	49467038
25_4126	ctg7180001825280_1428_SAG	49471584

25_4127	ctg7180001626876_2452_SCT	49500268
25_4128	ctg7180001891134_1034_SAC	49527617
25_4129	ctg7180001366224_973_SAG	49538958
25_4130	ctg7180001917264_14363_SAG	49560287
25_4131	ctg7180001917264_14500_SGT	49560424
25_4132	ctg7180001814595_506_SGT	49565640
25_4133	ctg7180001814595_2045_SAT	49567179
25_4134	ctg7180001814595_2066_SCT	49567200
25_4135	ctg7180001814595_2525_SCT	49567661
25_4136	ctg7180001814595_2986_SAG	49568120
25_4137	ctg7180001814595_5888_SAC	49570935
25_4138	ctg7180001814595_5899_SGT	49570946
25_4139	ctg7180001814595_5998_SAG	49571045
25_4140	ctg7180001814595_6958_SAG	49572005
25_4141	ctg7180001491120_2156_SAG	49578527
25_4142	ctg7180001491120_7824_SGT	49584195
25_4143	ctg7180001491120_9110_SGT	49585481
25_4144	ctg7180001491120_10291_SAG	49586662
25_4145	ctg7180001269258_2007_SGT	49589296
25_4146	ctg7180001811388_2581_SAC	49629403
25_4147	ctg7180001868124_713_SGT	49638873
25_4148	ctg7180001685805_289_SCT	49667892
25_4149	ctg7180001361709_951_SAG	49698931
25_4150	ctg7180001911886_2474_SAG	49708078
25_4151	ctg7180001915126_1285_SAG	49787367
25_4152	ctg7180001878294_520_SAG	49794951
25_4153	ctg7180001487266_255_SCT	49797979
25_4154	ctg7180001842376_1175_SAG	49831828
25_4155	ctg7180001842376_622_SAG	49832381
25_4156	ctg7180001842376_382_SAG	49832621
25_4157	ctg7180001270103_322_SAG	49879734
25_4158	ctg7180001801173_950_SAC	49883141
25_4159	ctg7180001838469_4267_SAC	49981559
25_4160	ctg7180001838469_5161_SGT	49982453
25_4161	ctg7180001825251_3007_SCT	50026867

25_4162	ctg7180001889743_2295_SCT	50135597
25_4163	ctg7180001896375_1326_SAG	50179106
25_4164	ctg7180001728229_239_SAC	50231415
25_4165	ctg7180001381762_254_SGT	50236469
25_4166	ctg7180001836461_6587_SAG	50262191
25_4167	ctg7180001836461_4213_SGT	50264565
25_4168	ctg7180001906699_3412_SAG	50304322
25_4169	ctg7180001921136_7797_SCT	50352356
25_4170	ctg7180001277566_179_SAC	50368376
25_4171	ctg7180001856283_1341_SAG	50396144
25_4172	ctg7180001858674_5334_SCT	50455036
25_4173	ctg7180001203968_852_SAG	50504436
25_4174	ctg7180001929167_4678_SAC	50542855
25_4175	ctg7180001904375_1222_SAG	50562122
25_4176	ctg7180001929116_553_SGT	50567164
25_4177	ctg7180001929116_611_SCT	50567222
25_4178	ctg7180001906729_2356_SCT	50591708
25_4179	ctg7180001920247_3175_SAC	50623278
25_4180	ctg7180001903104_6211_SAG	50638861
25_4181	ctg7180001884028_2884_SAG	50725536
25_4182	ctg7180001933753_748_SGT	50739941
25_4183	ctg7180001532938_952_SCT	50743170
25_4184	ctg7180001267313_1354_SGT	50777157
25_4185	ctg7180001267313_1276_SCT	50777235
25_4186	ctg7180001267313_1269_SAG	50777242
25_4187	ctg7180001453709_1157_SGT	50841185
25_4188	ctg7180001453709_1044_SCT	50841298
25_4189	ctg7180001600782_509_SAC	50871474
25_4190	ctg7180001600782_618_SAG	50871583
25_4191	ctg7180001922367_3207_SAC	50889163
25_4192	ctg7180001376918_1787_SCT	50904786
25_4193	ctg7180001877963_2464_SAC	50916084
25_4194	ctg7180001282041_58_SAG	50922856
25_4195	ctg7180001282041_111_SCT	50922909
25_4196	ctg7180001900824_130_SAG	50932193

25_4197	ctg7180001854194_3183_SGT	50962078
25_4198	ctg7180001894459_2585_SAG	50973310
25_4199	ctg7180001905834_497_SGT	50985614
25_4200	ctg7180001810098_4621_SCT	50989159
25_4201	ctg7180001906048_2041_SAG	51014461
25_4202	ctg7180001906048_3492_SAG	51015912
25_4203	ctg7180001697667_1101_SCT	51061714
25_4204	ctg7180001340116_83_SGT	51075638
25_4205	ctg7180001816334_3883_SGT	51108559
25_4206	ctg7180001401133_271_SGT	51121409
25_4207	ctg7180001921810_6909_SAC	51204262
25_4208	ctg7180001921810_6746_SAG	51204425
25_4209	ctg7180001921810_6714_SAG	51204457
25_4210	ctg7180001921810_5051_SAC	51207162
25_4211	ctg7180001823555_414_SAG	51245590
25_4212	ctg7180001902995_176_SAG	51300446
25_4213	ctg7180001822285_5090_SCT	51306624
25_4214	ctg7180001892442_4529_SAC	51365809
25_4215	ctg7180001826865_2141_SAC	51387502
25_4216	ctg7180001662629_4067_SGT	51402760
25_4217	ctg7180001806503_8245_SAC	51413910
